# Competing Demographic Drivers of Hospital Expenditures: Coexistence of the Red Herring and the Steepening Effects

**DOI:** 10.1002/hec.70092

**Published:** 2026-03-07

**Authors:** Malene Kallestrup‐Lamb, Alexander O. K. Marin, Jes Søgaard

**Affiliations:** ^1^ Aarhus University and PeRCent Aarhus Denmark; ^2^ University of Southern Denmark, CPop Odense Denmark

**Keywords:** expenditure decomposition, hospital expenditures, population aging, Red Herring, Steepening

## Abstract

The fiscal sustainability of healthcare systems is increasingly strained by aging populations with two competing hypotheses dominating the literature. The Red Herring Hypothesis suggests that healthcare expenditures are driven more by proximity to death than by chronological age, while the Steepening Hypothesis examines whether expenditures increase faster for older individuals over time. Nevertheless, these two frameworks have traditionally been studied independently rather than in conjunction. This paper offers a unified econometric model, allowing for a rigorous assessment of their concurrent validity and interaction. Using comprehensive register‐based monthly somatic hospital expenditure data for the entire Danish population from 2002 to 2017, we provide robust evidence that both Red Herring and Steepening effects operate simultaneously. Although Red Herring effects modestly mitigate the expenditure burden of an increasingly older population, they are substantially outweighed by pronounced Steepening effects, which account for nearly 60% of hospital expenditure growth during the observation period. Through a novel decomposition method, we identify a previously unrecognized interaction between these phenomena, a Red Herring Steepening effect, which accelerates expenditure growth in the final years of life for older individuals. Our findings suggest that healthcare systems face considerably greater fiscal challenges from population aging than previously recognized under the Red Herring Hypothesis alone.

## 1 Introduction

Healthcare expenditures constitute a significant portion of public and private spending in high‐income countries. In 2019, healthcare accounted for 16.8% of the U.S. gross domestic product (GDP), compared to 10.2% in the Euro area (WHO 2024). A major challenge facing healthcare systems is the increasing number of older individuals (K. Christensen et al. 2009). As the large baby boomer generation enters old age, the demand for healthcare services grows (Ricketts 2011), and concerns over escalating healthcare expenditures intensify.

Two major hypotheses have emerged in the literature, to address the relationship between the population age structure and healthcare expenditures. The Red Herring Hypothesis posits that time‐to‐death, rather than age, is the primary determinant of healthcare expenditures (Zweifel et al. 1999), a claim repeatedly supported in the context of hospital expenditures (Seshamani and Gray 2004a; Kallestrup‐Lamb et al. 2024). Empirical analyses show that accounting for time‐to‐death substantially reduces projected hospital expenditures relative to a Naïve scenario that excludes it (Polder et al. 2006; Bjørner and Arnberg 2012; Geue et al. 2014). Thus, simply having an older population does not automatically mean proportionally higher expenditures, although the impact of population aging on healthcare expenditures is more nuanced (Breyer and Lorenz 2021). The Steepening Hypothesis argues that the age‐expenditure profile becomes steeper over time (Buchner and Wasem 2006), meaning that healthcare expenditures for older individuals rise more than those for younger individuals, typically considered in absolute terms. The literature remains divided over the existence of Steepening effects. Felder and Werblow (2008) reject the hypothesis in the context of Switzerland, whereas more recent empirical evidence supports the presence of Steepening effects for hospital expenditures in Norway (Gregersen 2014), and for hospital expenditures plus physician care in Denmark (Kollerup et al. 2022). Unlike the Red Herring Hypothesis, the Steepening Hypothesis presents a more concerning outlook, suggesting that an increasing number of older individuals will drive significant increases in healthcare expenditures due to expenditure growth at older ages.

A critical point is that the Red Herring and Steepening Hypotheses are independent mechanisms. The Red Herring Hypothesis states that time‐to‐death, not age, drives healthcare expenditures. It predicts that individuals close to death incur high expenditures regardless of their age, while survivors of the same age incur low expenditures. The Steepening Hypothesis states that the age‐expenditure gradient steepens over calendar time. It predicts that expenditures grow faster for older age groups than for younger age groups across successive years. These are distinct concepts: The Red Herring Hypothesis concerns the cross‐sectional relationship between age, time‐to‐death, and expenditures in a given year. The Steepening Hypothesis concerns temporal changes in how expenditures vary by age. As one hypothesis addresses cross‐sectional variation while the other addresses temporal variation, both can be true simultaneously. Thus, rejecting the effects associated with one hypothesis does not imply rejecting those of the other hypothesis (Gregersen 2014). Meanwhile, the effects of these two hypotheses have not been formally tested within a unified statistical framework.

This paper presents the first nested investigation of the Red Herring and Steepening Hypotheses using detailed register data for the entire Danish population aged 30 and above, focusing on monthly somatic hospital expenditures from 2002 to 2017. We introduce a novel parametrization that allows for rigorous hypothesis testing, filling a notable gap in the literature, which has seen limited application of formal hypothesis testing. We propose using the Delta Method (Dorfman 1938) to estimate marginal nominal effects and use the Wald (1943) test to assess whether the marginal expenditure effects support the effects of the two hypotheses. Additionally, we introduce a decomposition approach that is new to this literature, demonstrating how changes in hospital expenditures can be attributed to each hypothesis.

Empirically, we present robust evidence supporting the coexistence of the effects of both hypotheses, with their relative influence varying by age and time‐to‐death. We perform sex‐disaggregated analyses across subsamples of the population, offering a more flexible framework for rigorous analysis and hypothesis testing within a demographic subgroup. Results uncover how age effects, time‐to‐death effects, and Steepening effects differ by sex, age, and time‐to‐death, confirmed through Welch tests. Specifically, while both the Red Herring and Steepening effects are present across all groups, time‐to‐death effects increase in age. Steepening is particularly strong for ages 50 to 80, while the age‐specific growth in somatic hospital expenditures among those 80+ years old is lower. Tests including a Bayesian hypothesis testing approach (Kass and Raftery 1995) and an expanded model parametrization further validate these findings. Applying our decomposition framework, we find that the Steepening‐related effect is the primary driver of hospital expenditure growth, accounting for 60% of the increase, while the Red Herring effect mitigates only 19% of the expenditure growth. These estimates reflect the temporal changes in average hospital expenditures, including underlying technology, and demographic shifts during 2002–2017, and should not be interpreted as universal constants. Additionally, we identify a novel interaction between the two hypotheses, demonstrating that the mitigating effect posited by the Red Herring Hypothesis can exhibit an age‐specific growth pattern, characteristic of the Steepening Hypothesis. This interaction term accounts for 19% of the change in hospital expenditures and precisely offsets the Red Herring mitigation in the decomposition.

The remainder of this paper is structured as follows: Section 2 outlines the model, estimation approach, hypothesis tests, and decomposition method. Section 3 describes the dataset, while Section 4 presents the empirical findings. Section 5 discusses the implications of our results, and Section 6 concludes.

## 2 Methodology

To analyze the relationship between hospital expenditures and the demographic drivers related to population aging, we employ the widely used two‐part model (Cragg 1971; Seshamani and Gray 2004a), a framework predominantly used in existing literature (Kallestrup‐Lamb et al. 2024). This model is particularly well‐suited for capturing the distinctive distribution of hospital expenditures, which is characterized by a large number of zero observations and a heavy right tail for nonzero expenditures; see Deb and Norton (2018) for a comprehensive overview of this approach. The two‐part model is defined by the following probability density function

(1) $$\begin{array}{c} \psi \left(H_{i}\right)=F\left(X_{i}^{\prime }\delta \right)g\left(X_{i}^{\prime }\gamma \right). \end{array}$$

The first component captures the probability that an individual incurs positive hospital expenditures, $H_{i}>0$, conditional on $\left(k\times 1\right)$ explanatory variables, $X_{i}$, and their associated parameters, δ. Specifically, this probability is expressed as $\mathrm{Pr}\left(H_{i}>0|X_{i}\right)=F\left(X_{i}^{\prime }\delta \right)$, where F is a cumulative density function. We refer to the probability of incurring hospital expenditures as the *extensive margin*. The second component models the distribution of positive hospital expenditures, $H_{i}|X_{i},H_{i}>0$, as $g\left(X_{i}^{\prime }\gamma \right)$, where g is a density function and γ a k‐dimensional parameter vector. This component provides insights into the *intensive margin*, describing the extent of resource utilization once individuals enter the hospital system. When the realized value from the first part is zero, individual i incurs no hospital expenditures. Conversely, if the realized value is one, hospital expenditures are drawn randomly from the second part, $g\left(\cdot \right)$, yielding a positive amount.

To estimate the parameters of our model in Equation (1) we use quasi maximum likelihood estimation, following existing literature (Atella and Conti 2014; Moore et al. 2017).^1^ The first part of the model utilizes a Probit specification, similar to that used by Seshamani and Gray (2004b), Atella and Conti (2014), and Moore et al. (2017). The second part, $g\left(\cdot \right)$, is modeled using a Poisson‐distributed generalized linear model with a log‐link, as suggested by (Carreras et al. 2018; von Wyl 2019). This two‐part model is further supported by Kollerup et al. (2022), who identify the Poisson‐distributed generalized linear model with a log‐link as the most appropriate for analyzing Danish hospital expenditure data using the modified Park's test (Manning and Mullahy 2001). Additional details on the estimation of two‐part models can be found in Belotti et al. (2015).

The estimated parameters, δ and γ, enter the model through nonlinear Probit and Poisson functions, respectively, and lack a straightforward interpretation. However, understanding marginal effects is essential for evaluating the Red Herring and Steepening hypotheses, which focus on the direction and magnitude of partial derivatives rather than on the coefficients themselves. For example, the Red Herring Hypothesis states that hospital expenditures increase substantially as time‐to‐death shortens, while the Steepening Hypothesis posits a positive second derivative of expenditures with respect to age and time. These effects cannot be directly assessed from parameter estimates, fitted values, or conditional means, which do not convey the uncertainty needed for hypothesis testing. To address this, we apply the Delta Method (Dorfman 1938) to estimate unbiased marginal effects for key variables such as age, time‐to‐death, and their interaction, complete with asymptotic properties. Although fully parameterized models are often evaluated via predicted outcomes (see, e.g., Seshamani and Gray (2004b) and Kollerup et al. (2022)), such predictions should not be mistaken for marginal effects. Our use of the Delta Method represents a methodological advance in testing the Red Herring and Steepening Hypotheses by enabling formal inference. Specifically, we report the sample average of individual marginal effects, rather than marginal effects at the sample mean.^2^ ^,^ ^3^

### 2.1 Linear Parametrization

This section provides a detailed examination of the linear arguments, $X_{i}^{\prime }\delta$ and $X_{i}^{\prime }\gamma$, in Equation (1). Specifically, it explores how these terms capture the relationship between demographic drivers and hospital expenditures, highlighting key variations in expenditure dynamics across time and age. In research on hospital expenditures and the demographic composition of a population, individual age plays a crucial role, as expenditures typically increase with advancing age (Howdon and Rice 2018; Maynou et al. 2023; Laudicella et al. 2022). The assumption that age, A, is the primary determinant of an individual's, i, hospital expenditures, is commonly referred to as the *Naïve* model (van Baal and Wong 2012). In Equation (2), age serves as the primary variable of interest, while additional control variables, $\overset{ˇ}{X}$, are incorporated to account for other influencing factors.

(2) $$Na\ddot{i}ve:\begin{array}{cc} X_{i}^{\prime }\delta^{N} & =A_{i}\delta_{A}^{N}+{\overset{ˇ}{X}}_{i}^{\prime }\delta_{\overset{ˇ}{X}}^{N}\\ X_{i}^{\prime }\gamma^{N} & =A_{i}\gamma_{A}^{N}+{\overset{ˇ}{X}}_{i}^{\prime }\gamma_{\overset{ˇ}{X}}^{N}. \end{array}$$

In their seminal work, Zweifel et al. (1999) argue that once an individual's proximity to death is accounted for, age has a limited effect on hospital expenditures. Consequently, while age appears to be a highly significant predictor in the Naïve model, this significance primarily reflects omitted variable bias due to the exclusion of time‐to‐death, TTD, as a regressor. Equation (3) considers a parametrization of the Red Herring model by introducing the time‐to‐death regressor and associated effects to the Naïve model in Equation (2).^4^ Following Moore et al. (2017), we include a binary indicator, ${\overset{ˇ}{X}}^{*}$, which equals one if time‐to‐death is censored,^5^ indicating that the individual is still alive

(3) $$\mathrm{Red}\;\mathrm{Herring}:\begin{array}{cc} X_{i}^{\prime }\delta^{RH} & =TTD_{i}\delta_{TTD}^{RH}+A_{i}\delta_{A}^{RH}+{\overset{ˇ}{X}}_{i}^{\prime }\delta_{\overset{ˇ}{X}}^{RH}+{\overset{ˇ}{X}}_{i}^{*}\delta_{{\overset{ˇ}{X}}^{*}}^{RH}\\ X_{i}^{\prime }\gamma^{RH} & =TTD_{i}\gamma_{TTD}^{RH}+A_{i}\gamma_{A}^{RH}+{\overset{ˇ}{X}}_{i}^{\prime }\gamma_{\overset{ˇ}{X}}^{RH}+{\overset{ˇ}{X}}_{i}^{*}\gamma_{{\overset{ˇ}{X}}^{*}}^{RH}. \end{array}$$

Buchner and Wasem (2006) proposed a supplementary hypothesis on the relationship between hospital expenditures and the population age structure, known as the Steepening Hypothesis. As defined by Felder and Werblow (2008), “Steepening … means that the growth rate of healthcare expenditures rises with an increasing age” which implies that the hospital expenditures of older individuals grow at a faster rate compared to younger individuals. In this paper, we consider growth in absolute terms following most analyses in existing literature (Buchner and Wasem 2006; Felder and Werblow 2008; Gregersen 2014) and focuses on individual level expenditures like Kollerup et al. (2022).^6^ Gregersen (2014) proposes a parametrization of a hospital expenditure equation suitable for testing the existence of a Steepening effect. This approach includes an interaction between age and calendar time, A⋅T, where T represents calendar time. If the coefficient estimate on the age‐time interaction is positive, the age profile of hospital expenditures steepens over time for a linear model. Meanwhile, for nonlinear specifications of hospital expenditures, such as Equation (1), Steepening effects can occur despite a null age‐time interaction, which we provide a general theoretical example of in Supporting Information S1: Online Appendix B.1. We address how to assess Steepening effects for such models in the following section. We formulate Equation (4), which incorporates the Steepening Hypothesis by extending the Naïve model in Equation (2) with time and an age‐time interaction term.^7^ Crucially, the Steepening Hypothesis disregards any influence of time‐to‐death as argued under the Red Herring Hypothesis.

(4) $$Steepening:\begin{array}{cc} X_{i}^{\prime }\delta^{S} & =A_{i}\delta_{A}^{S}+T_{i}\delta_{T}^{S}+A_{i}T_{i}\delta_{A\cdot T}^{S}+{\overset{ˇ}{X}}_{i}^{\prime }\delta_{\overset{ˇ}{X}}^{S}\\ X_{i}^{\prime }\gamma^{S} & =A_{i}\gamma_{A}^{S}+T_{i}\gamma_{T}^{S}+A_{i}T_{i}\gamma_{A\cdot T}^{S}+{\overset{ˇ}{X}}_{i}^{\prime }\gamma_{\overset{ˇ}{X}}^{S}. \end{array}$$

To develop a comprehensive framework that unifies the Red Herring and Steepening Hypotheses, we introduce a novel parameterization that seamlessly integrates both perspectives. This approach enables more precise estimation of how hospital expenditures evolve with individual aging, capturing both time‐to‐death effects and age‐related expenditure dynamics within a single, cohesive model. While Gregersen (2014) demonstrates that the Red Herring and Steepening Hypotheses can both be valid, a formal model that integrates the two hypotheses has yet to be developed. In this paper, we address this gap by proposing a novel parameterization that enables such testing. In Equation (5), we extend the Naïve model by incorporating the time‐to‐death effects from the Red Herring Hypothesis, along with calendar time and the age‐time interaction from the Steepening Hypothesis.

(5) $$\mathrm{Joint}\;\mathrm{Model}:\begin{array}{cc} X_{i}^{\prime }\delta & =TTD_{i}\delta_{TTD}+A_{i}\delta_{A}+T_{i}\delta_{T}+A_{i}T_{i}\delta_{A\cdot T}+{\overset{ˇ}{X}}_{i}^{\prime }\delta_{\overset{ˇ}{X}}+{\overset{ˇ}{X}}_{i}^{*}\delta_{{\overset{ˇ}{X}}^{*}}\\ X_{i}^{\prime }\gamma & =TTD_{i}\gamma_{TTD}+A_{i}\gamma_{A}+T_{i}\gamma_{T}+A_{i}T_{i}\gamma_{A\cdot T}+{\overset{ˇ}{X}}_{i}^{\prime }\gamma_{\overset{ˇ}{X}}+{\overset{ˇ}{X}}_{i}^{*}\gamma_{{\overset{ˇ}{X}}^{*}}. \end{array}$$

Related studies, such as von Wyl (2019) and Kollerup et al. (2022), run regressions including Steepening effects alongside indicators of being in the last year of life.^8^ von Wyl (2019) also includes an indicator of whether an individual dies in the next calendar year. Although these variables serve as crude proxies for time‐to‐death, our parametrization in Equation (5) is the first expression that allows for a rigorous statistical test of the joint validity of both hypotheses.

Our linear specifications in Equations ((2), (3), (4), (5)) are parsimonious, focusing solely on age, time‐to‐death, time, and the age‐time interaction, capturing the effects in a few key parameters which are easy to interpret. In contrast, existing literature often applies an expanded parametrization seeking to capture age, sex, and time‐to‐death patterns in a single regression (Atella and Conti 2014; von Wyl 2019; Kollerup et al. 2022) which can be challenging to analyze and interpret due to the high dimensionality of its inputs. To explore heterogeneous patterns across subgroups, our sparse parametrization enables analyses of subgroups of individuals across age, sex, and time‐to‐death, where a parsimonious model likely offers a better fit. Meanwhile, we also conduct a robustness check with an expanded parametrization, as discussed in Supporting Information S1: Online Appendix C.1. Regressors such as health and income have also been considered (Carreras et al. 2018; Werblow et al. 2007; Kollerup et al. 2022; Gregersen 2014), yet, as these factors are beyond the scope of the Red Herring and Steepening Hypotheses as defined in the literature (Zweifel et al. 1999; Buchner and Wasem 2006) we exclude them from our analysis although we acknowledge their potential impact on the aging‐hospital expenditure relationship. Meanwhile, before an assessment of the potentially mediating effects of these factors can be valued, it is imperative to first establish a comprehensive understanding of the Red Herring and the Steepening effects.

### 2.2 Parameter Hypotheses

A key feature of our parameterization in Equation (5) is its ability to perform rigorous statistical testing on whether the effects of neither hypothesis is valid, only one holds, or the effects are simultaneously valid. Specifically, Equation (6) defines three hypotheses on the parameters associated with age, time‐to‐death, and the age‐time interaction. The first null hypothesis, $H_{0}^{1}$, asserts that the Naïve model in Equation (2) is correct, implying parameters on time‐to‐death, time, and the age‐time interaction are set to zero. The second null hypothesis, $H_{0}^{2}$, upholds the Red Herring equation, setting all parameters related to age and the Steepening Hypothesis to zero. Lastly, the third null hypothesis, $H_{0}^{3}$, assumes the Steepening specification in Equation (5) is correct, setting the time‐to‐death parameters to zero. If all three null hypotheses are rejected, we accept the alternative hypothesis that all parameters are nonzero, indicating that the joint model with both Red Herring and Steepening effects is preferred.

(6) $$\begin{array}{cc} H_{0}^{1}:\; & \delta_{TTD}=\gamma_{TTD}=\delta_{T}=\gamma_{T}=\delta_{A\cdot T}=\gamma_{A\cdot T}=0,\\ H_{0}^{2}:\; & \delta_{A}=\gamma_{A}=\delta_{T}=\gamma_{T}=\delta_{A\cdot T}=\gamma_{A\cdot T}=0,\\ H_{0}^{3}:\; & \delta_{TTD}=\gamma_{TTD}=0. \end{array}$$

Since our parameter hypotheses relate to multiple parameters, standard *t*‐tests are not applicable. Instead, we apply the Wald test, which allows us to test multi‐parameter hypotheses. The Wald test has previously been used for statistical tests of the Red Herring Hypothesis (Zweifel et al. 1999).^9^ In conducting the Wald test, it is essential to account for the joint uncertainty of the parameters, δ and γ, arising from both parts of the model. To address this, we estimate the variance of ${\left(\delta^{\prime },\gamma^{\prime }\right)}^{\prime }$ and apply a cluster‐robust variance estimator (Liang and Zeger 1986), with clustering at the individual level, to properly capture the dependencies in the data.

Although the hypothesis tests in Equation (6) provide important insights into how a model can be parameterized to encompass regressors related to both Red Herring and Steepening effects, the parametrization tests do not necessarily guarantee that the effects of the Red Herring and Steepening Hypotheses are nonzero. Specifically, Steepening effects can still be observed without an age‐time interaction, since the second‐order derivative of F, g, or the two part model, ψ, with respect to age and time can still be significant, especially for nonlinear functions (see Supporting Information S1: Online Appendix B.1 for derivations). To assess whether the average marginal effects for the Red Herring or Steepening effects are nonzero, as a supplement, we set up equivalent tests to those in Equation (6) but do so with respect to the average marginal effects. Specifically, we test

(7) $$\begin{array}{cc} H_{0}^{me,1}:\; & E\left[\begin{array}{ccc} \frac{\partial f\left(H_{i},\theta \right)}{\partial TTD} & \frac{\partial f\left(H_{i},\theta \right)}{\partial T} & \frac{\partial f\left(H_{i},\theta \right)}{\partial A\cdot T} \end{array}\right]=\left[\begin{array}{ccc} 0 & 0 & 0 \end{array}\right],\\ H_{0}^{me,2}:\; & E\left[\begin{array}{ccc} \frac{\partial f\left(H_{i},\theta \right)}{\partial A} & \frac{\partial f\left(H_{i},\theta \right)}{\partial T} & \frac{\partial f\left(H_{i},\theta \right)}{\partial A\cdot T} \end{array}\right]=\left[\begin{array}{ccc} 0 & 0 & 0 \end{array}\right],\\ H_{0}^{me,3}:\; & E\left[\begin{array}{c} \frac{\partial f\left(H_{i},\theta \right)}{\partial TTD} \end{array}\right]=\left[\begin{array}{c} 0 \end{array}\right], \end{array}$$

for the full two‐part model where f=ψ, at the extensive margin with f=F, and at the intensive margin as f=g. These hypotheses can be implemented with Wald tests on the marginal effects as calculated with the Delta Method as discussed in Supporting Information S1: Online Appendix B.2. A rejection of all three null hypotheses, $H_{0}^{me,1}$ to $H_{0}^{me,3}$, leads us to accept the alternative hypotheses of coexistent Red Herring and Steepening effects, for a given function, f.

To address potential limitations of classical hypothesis testing with large samples,^10^ we supplement Wald tests the Bayesian hypothesis test of Kass and Raftery (1995). Unlike Wald tests that evaluate the probability of observing data D given the null, $\mathrm{Pr}\left(D|H_{0}\right)$, Bayesian *p*‐values assess $\mathrm{Pr}\left(H_{0}|D\right)$, that is, the probability of the null hypothesis given data. From Bayes's theorem

(8) $$\mathrm{Pr}\left(H_{0}|D\right)=\frac{\text{Bayes}\;\text{factor}\times \text{prior}\;\text{odds}}{1+\text{Bayes}\;\text{factor}\times \text{prior}\;\text{odds}}.$$

This approach evaluates relative model evidence rather than absolute rejection thresholds, making it less prone to rejecting null hypotheses for trivially small departures when samples are very large.^11^ See Supporting Information S1: Online Appendix B.3 for mathematical details.

Importantly, the hypotheses tests in Equations (6) and (7) assess specific parameters and marginal associations, rather than establishing mutual exclusivity between effects. Rejecting the null hypothesis that time‐to‐death parameters $\left(H_{0}^{3}\right)$ or effects $\left(H_{0}^{me,3}\right)$ equal zero does not automatically confirm that only Red Herring effects matter; it simply indicates that a model excluding time‐to‐death is misspecified. Similarly, rejecting $H_{0}^{1}$ or $H_{0}^{me,1}$ (the Naïve model) does not determine whether Red Herring or Steepening mechanisms dominates, as both could operate simultaneously (Gregersen 2014). To assess whether both effects coexist, our strategy is to employ rigorous testing. The convergence of results across these varied specifications and testing frameworks strengthens our inference that both Red Herring and Steepening effects contribute meaningfully to explaining hospital expenditure patterns, and that omitting either results in model misspecification.

### 2.3 Decomposing Hospital Expenditure Changes

To assess the relative contributions of the Red Herring and Steepening Hypotheses to rising hospital expenditures, we introduce a new decomposition approach, novel to this literature, which shares similarities with the Kitagawa (1955) and the Oaxaca (1973)‐Blinder (1973) decompositions. This framework quantifies how changes in population structure and hospital spending patterns drive aggregate hospital expenditures between two periods, $t_{1}$ and $t_{2}$.

The decomposition is based on a hospital expenditure model, similar to van Baal and Wong (2012), that facilitates the attribution of expenditure changes to the different model concepts discussed in Equations ((2), (3), (4), (5)). Besides the concepts, this decomposition presents a different analytical concept from the regression analysis discussed above. We define total annual somatic hospital expenditures, $H_{T}$, as the sum of expenditures across individuals grouped by age, A, and time‐to‐death, TTD, in period, T,

(9) $$H_{T}:=\sum_{A}\sum_{TTD}N_{T,A,TTD}{\bar{H}}_{T,A,TTD},$$

where $N_{T,A,TTD}$ is the number of individuals in each group, and ${\bar{H}}_{T,A,TTD}$ is their average hospital expenditure. The total change in expenditures, $H_{t_{1}}-H_{t_{2}}$, can be decomposed into two broad components,

(10) $$\underset{\text{Population}\;\text{Change}}{\underbrace{\sum_{A}\sum_{TTD}\left[N_{t_{1},A,TTD}-N_{t_{2},A,TTD}\right]{\bar{H}}_{t_{1},A,TTD}}}+\underset{\text{Expenditure}\;\text{Change}}{\underbrace{\sum_{A}\sum_{TTD}\left[{\bar{H}}_{t_{1},A,TTD}-{\bar{H}}_{t_{2},A,TTD}\right]N_{t_{2},A,TTD},}}$$

relating to the change in the population size and the change in mean hospital expenditures, respectively.

A more detailed four‐part decomposition, derived from Equation (10) in Appendix A, further isolates key drivers

(11) $$\begin{array}{cc} & \underset{\text{Naïve}\;}{\underbrace{\sum_{A}\left[N_{t_{1},A}-N_{t_{2},A}\right]{\bar{H}}_{t_{1},A}}}+\underset{\text{Red}\;\text{Herring}\;\text{Error}}{\underbrace{\sum_{A}\sum_{TTD}\left[N_{t_{1},A,TTD}-N_{t_{2},A,TTD}\right]\left[{\bar{H}}_{t_{1},A,TTD}-{\bar{H}}_{t_{1},A}\right]}}+\\ & \underset{\text{Steepening}\;\text{effect}}{\underbrace{\sum_{A}\left[{\bar{H}}_{t_{1},A}-{\bar{H}}_{t_{2},A}\right]N_{t_{2},A}}}+\underset{\text{Red}\;\text{Herring}‐\text{Steepening}\;}{\underbrace{\sum_{A}\sum_{TTD}\left[\left({\bar{H}}_{t_{1},A,TTD}-{\bar{H}}_{t_{1},A}\right)-\left({\bar{H}}_{t_{2},A,TTD}-{\bar{H}}_{t_{2},A}\right)\right]N_{t_{2},A,TTD}}}. \end{array}$$

The *Naïve* term captures changes in aggregated hospital expenditures attributable to population shifts, $N_{t_{1},A}-N_{t_{2},A}$, assuming time‐invariant mean hospital expenditures, ${\bar{H}}_{t_{1},A}$, while ignoring time‐to‐death effects.^12^ Despite its simplicity, the Naïve term incorporates considerable detail by accounting for age‐structure in both population demographics and hospital expenditures through summation across age groups, A. The *Red Herring Error* term quantifies the systematic underestimation of near‐death expenditures, ${\bar{H}}_{t_{1},A,TTD}-{\bar{H}}_{t_{1},A}$, that occurs when age‐based projections exclude time‐to‐death information. The underestimation term remains time‐invariant at the $t_{1}$ level signifying that the Red Herring hypothesis addresses the relationship between age and time‐to‐death without making statements about intertemporal changes.

The third term captures age‐specific temporal changes in average hospital expenditures, ${\bar{H}}_{t_{1},A}-{\bar{H}}_{t_{2},A}$, closely resembling the conceptualization of the Steepening Hypothesis by Gregersen (2014) as an interaction between age, time, and healthcare expenditures. The term captures the age‐specific temporal changes, making the term more comprehensive than the pure age‐time interactions considered in the parametrization in Section 2.1 as broader time‐trends are also captured in this term. The final term in Equation (11) introduces temporal changes in the Red Herring error between periods $t_{1}$ and $t_{2}$ as $\left({\bar{H}}_{t_{1},A,TTD}-{\bar{H}}_{t_{1},A}\right)-\left({\bar{H}}_{t_{2},A,TTD}-{\bar{H}}_{t_{2},A}\right)$. We label this component the *Red Herring‐Steepening* term since it captures not only the time‐to‐death related expenditures addressed by the Red Herring Hypothesis but also the age‐specific time changes considered by the Steepening Hypothesis. Although this interaction of the two hypotheses is not explicitly parameterized in our main linear parametrization in Equation (5), subgroup analyses of the Steepening effect by time‐to‐death can yield insights into its presence within the regression framework.

An interesting result arises from the decomposition: the sum of the Red Herring Error term and the Red Herring‐Steepening term in Equation (11) must be zero (see Appendix A.1 for a proof). This implies that the Red Herring Error term is exactly equal in magnitude but opposite in sign to the Red Herring‐Steepening term. While the Red Herring Error term is often interpreted as a mitigating part of the expenditure increases associated with demographic changes, its influence is fully counterbalanced by growth rates of end‐of‐life expenditures, captured by the Red Herring‐Steepening term. As a result, although growth in expenditures during the final years of life has received comparatively limited attention in the literature, it plays a role equally important to the Red Herring Hypothesis itself in shaping long‐run trends in aggregate hospital expenditure.

## 3 Data

This paper leverages comprehensive Danish register data from Statistics Denmark (2023), tracked at the individual level, covering the period from 2002 to 2022. We include all individuals and, in line with existing literature, specifically focus on those aged 30 and older (Carreras et al. 2018; Felder et al. 2010; Werblow et al. 2007; Buchner and Wasem 2006). This selection includes the age groups that incur the highest hospital expenditures, as well as the ages at which hospital expenditures begin to rise. Our primary focus is on expenditures related to somatic hospital care. All hospital visits are documented in medical records and accessible to researchers, including the government‐insured hospital expenditures at both private and public hospitals. Government insurance covers nearly all hospital procedures in Denmark, with out‐of‐pocket expenses accounting for 4.6% of total hospital expenditures, while private healthcare insurance covers 0.9% of hospital expenditures (Insurance and Pension (FP) 2023). Somatic hospital expenditures represent a well‐defined and significant share of healthcare expenditures in Denmark amounting to 47% of total publicly funded healthcare expenditures in Denmark in 2017 (Statistics Denmark 2025).

Each interaction with a hospital is associated with a pseudonymized person identifier, allowing hospital services to be attributed to individual patients. Patients are admitted based on a referral diagnosis and receive either inpatient or outpatient care. The Danish Health Data Authority calculates Diagnosis‐Related Grouping (DRG) tariffs for hospital services at a highly detailed procedure‐specific level, as described in Statens Serum Institut (2014). These tariffs account for variable costs associated with performed procedures such as medical staff time, medical equipment, and most specialized pharmaceuticals, while attributing fixed costs to individual patients is generally considered impractical. If a patient remains hospitalized beyond the standard treatment duration for a specific procedure, additional costs for the extended stay are included. DRG‐based hospital expenditures are widely used in existing literature on Danish healthcare data (B. J. Christensen et al. 2016; Kollerup et al. 2022). For the hospital expenditure data, we consider the observation period from 2002 to 2017, during which we recorded 109,381,459 individual hospital interactions. Due to substantial changes in the hospital registers in 2018 and the absence of an established method to ensure cross‐year comparability, we restrict our analysis of hospital services to the period prior to 2018.

For our analysis, we aggregate hospital expenditures into monthly amounts, capturing all expenditures incurred from the first to the last day of each month.^13^ This allocation is possible due to detailed admission and discharge dates in the DRG registers. The use of monthly data strengthens our study by capturing the progressive increase in hospital expenditures as individuals near the end of life (B. J. Christensen et al. 2016). Additionally, this approach helps prevent the underestimation of hospital expenditures for individuals with less than a year to live, a limitation often associated with annual data. To ensure the accuracy of our hospital expenditure data, we confirm that the aggregate annual expenditures in our dataset matches the raw DRG register data and the age‐specific averages of hospital expenditures in B. J. Christensen et al. (2016) and Kollerup et al. (2022).^14^

From the population register (BEF) (Statistics Denmark 2024), we obtain demographic information, including sex and age. The time‐to‐death of deceased individuals is determined using the date of birth from the population register (BEF) and the date of death from the deaths register (DODAASG) (Danish Health Data Authority 2024), which covers data through the end of 2022. This approach allows us to precisely determine the time‐to‐death for all individuals within the 4 years preceding their passing, offering a detailed and comprehensive perspective on hospital utilization during the final stages of life. Seshamani and Gray (2004b) find that time‐to‐death effects on hospital expenditures become negligible beyond 4 years, and our dataset spans a sufficient duration to capture these effects.^15^ Both age and time‐to‐death are measured in years, with decimal values representing additional days. We measure calendar time in years at a monthly resolution as $\left(year-2002\right)+\left(month-1\right)/12$, setting January 1, 2002, as the base date. After combining the registers, we are left with 638,184,716 monthly observations, corresponding to 3,323,879 person‐years annually.^16^

### 3.1 Descriptive Statistics

**FIGURE 1:**
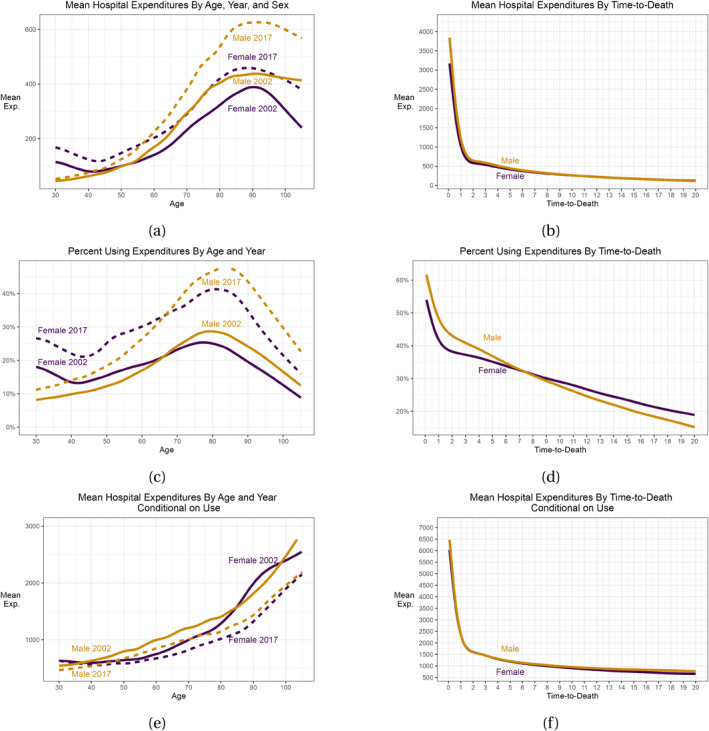
Summary statistics by age, time‐to‐death, year, and sex. (a) Mean monthly hospital expenditures by age for all individuals; (b) mean monthly hospital expenditures by time‐to‐death for all individuals; (c) percent using hospital expenditures within a month by age for all individuals; (d) percent using hospital expenditures within a month by time‐to‐death for all individuals; (e) mean monthly hospital expenditures by age for individuals with positive hospital expenditures; and (f) mean monthly hospital expenditures by time‐to‐death for individuals with positive hospital expenditures. All amounts are expressed in 2020 U.S. dollars.

To gain an initial understanding of how hospital expenditures correlate with key variables, we present mean hospital expenditures in Figure 1, stratified by age, sex, time‐to‐death, and time period.

Mean hospital expenditures by age inform about the Naïve model of Equation (2). In Figure 1a, mean hospital expenditures are shown by age, with a solid purple line representing females in 2002 and a solid orange line representing males in 2002. The dashed lines show their 2017 equivalents. We observe that average hospital expenditures generally increase with age, peaking around age 90, after which they level off. An exception occurs for females in their 30s and early 40s, where expenditures are higher due to reproductive health demands. This pattern is mirrored on the extensive margin in 1c, which illustrates the outcome of the first part of Equation (1), depicting the percentage of observations with any hospital expenditure by age. Notably, these probabilities peak slightly earlier, around age 80. In contrast, the intensive margin—the second part of Equation (1), shown in 1e—reveals a different trend. Among individuals with positive hospital expenditures, mean expenditures rise consistently with age, indicating that older individuals incur higher expenditures in months with admissions.

We also examine the relationship between hospital expenditures and time‐to‐death for deceased individuals, which is a key focus of the Red Herring hypothesis. Figure 1b demonstrates that mean hospital expenditures peak within a month of death and then decline sharply until approximately 1.5 years prior to death. After this point, expenditures decrease more gradually in an almost linear fashion. This trend holds true for both sexes and aligns with findings from previous studies, such as French et al. (2017). For the first stage of the two‐part model in Equation (1), the probability of incurring hospital expenditures, Figure 1d shows that this probability is highest close to death, with many individuals receiving hospital care during their final months. The trend in Figure 1d mirrors that in Figure 1b, with an initial steep decline followed by a more gradual reduction, albeit with a different outcome measure. Notably, more than 50% of females and over 60% of males use hospital care in their last month of life. A pronounced sex disparity is observed, with males exhibiting a higher likelihood of hospitalization up to approximately 7.5 years prior to death compared to females. Finally, Figure 1f presents mean hospital expenditures by time‐to‐death for the subsample of individuals who use hospital care, corresponding to the second part of the two‐part model in Equation (1). This plot exhibits a similar rapid decline in expenditures until 1.5 years prior to death, but with larger mean values compared to Figure 1b, as individuals with zero hospital expenditures are excluded.

By comparing age‐specific hospital expenditures from 2002 to the averages in 2017, we derive descriptive insights into the Steepening Hypothesis for hospital expenditures in absolute terms. Comparing the solid line for 2002 data with the dashed line for 2017 in Figure 1a, we observe a widening gap in expenditures for males aged 30 to 90. At older ages, however, this difference narrows. For females, the trend is less pronounced, suggesting potential differences in Steepening patterns by age and sex. A similar pattern emerges in the proportion of observations utilizing hospital care, as shown in Figure 1c, which aligns with the first component of Equation (1). However, when examining mean expenditures among individuals with positive hospital care expenditures conditional on use, Figure 1e reveals a contrasting trend: for both sexes, mean hospital expenditures decreased between 2002 and 2017. This finding challenges conventional expectations about hospital spending trends and suggests that the Steepening Hypothesis may not consistently hold if hospital expenditures decrease in age and time. Specifically, in the second component of the two‐part model, average hospital expenditures per user have declined over time across most age groups for both sexes.

## 4 Results

This section presents the findings of our study for the full population of individuals. Table 1 displays the regression results derived from the model specified in Equation (1), utilizing four distinct parameterizations of the Probit and Poisson models, as outlined in Equations ((2), (3), (4), (5)). Additionally, we provide estimates of the nominal marginal association, $E_{\psi }$, for the two‐part model, computed using the Delta Method, as detailed in Section 2.2. Our computational resources enable the estimation of our models for a random subsample of 10,000,000 observations.

**TABLE 1:** GLM model estimates.

Model:	Naïve			Red Herring			Steepening			Joint Model		
	Probit	Poisson	$E_{\psi }$	Probit	Poisson	$E_{\psi }$	Probit	Poisson	$E_{\psi }$	Probit	Poisson	$E_{\psi }$
Age	0.014	0.017	6.940	0.012	0.003	3.43	0.011	0.018	6.880	0.008	0.003	3.320
	(0.004)	(0.000)	(1.890)	(0.003)	(0.000)	(0.517)	(0.003)	(0.000)	(1.896)	(0.002)	(0.000)	(0.504)
	[0.000]	[0.000]	[0.000]	[0.000]	[0.000]	[0.000]	[0.000]	[0.000]	[0.000]	[0.000]	[0.000]	[0.000]
	{3.93}	{223.61}	{8.82}	{3.91}	{114.25}	{8.82}	{3.66}	{134.62}	{8.93}	{3.51}	{55.99}	{8.95}
TTD	—	—	—	−0.101	−0.470	−117.00	—	—	—	−0.103	−0.470	−117.410
	(−)	(−)	(−)	(0.019)	(0.002)	(42.194)	(−)	(−)	(−)	(0.020)	(0.002)	(41.915)
	[−]	[−]	[−]	[0.000]	[0.000]	[0.006]	[−]	[−]	[−]	[0.000]	[0.000]	[0.005]
	{−}	{−}	{−}	{−5.34}	{−193.20}	{−5.50}	{−}	{−}	{−}	{−5.25}	{−195.15}	{−5.61}
Age · Time	—	—	—	—	—	—	0.033	−0.011	11.440	0.037	0.003	9.150
	(−)	(−)	(−)	(−)	(−)	(−)	(0.006)	(0.001)	(1.659)	(0.006)	(0.000)	(0.785)
	[−]	[−]	[−]	[−]	[−]	[−]	[0.000]	[0.000]	[0.000]	[0.000]	[0.000]	[0.000]
	{−}	{−}	{−}	{−}	{−}	{−}	{5.98}	{−14.63}	{9.89}	{5.99}	{7.42}	{9.92}
Controls	Yes			Yes			Yes			Yes		
DoF	9,999,972			9,999,968			9,999,968			9,999,964		
Log lik.	−3,196,833,760			−2,906,760,856			−3,194,966,130			−2,906,648,274		

*Note:* Dependent variable is monthly somatic hospital expenditures in 2020 USD. The Probit and Poisson columns report parameter estimates from the two‐part model in Equation (1). $E_{\psi }$ reports the average marginal effect on expected monthly hospital expenditures from the full two‐part model. Standard errors in round parentheses (); *p*‐values in square brackets [ ]; Z‐scores in curly brackets { }. TTD is short for time‐to‐death. To ensure convergence in the numerical optimization (Greene 2017), Age⋅Time is divided by 100 in the Probit and Poisson regressions but not in $E_{\psi }$, which is the average marginal effect with respect to age and time. Controls refer to an intercept, month dummies, and a male dummy. The Red Herring and Joint Model also include ${\overset{ˇ}{X}}^{*}$ as a control while time is also included in the Steepening and Joint Model. DoF is shorthand for degrees of freedom. Models are estimated with 10,000,000 observations and 2,191,490 observations in the Poisson part.

The first three columns report estimates from the Naïve specification, which remains agnostic to the parameters of the Red Herring and Steepening Hypotheses by excluding time‐to‐death, age interacted with time, and time itself, incorporating only age and other control variables as regressors. We find that age is associated with an increase in the mean probability of incurring any hospital expenditure in the Probit model, as indicated by a positive parameter estimate of 0.014. Moreover, the *p*‐value reported in the squared brackets indicates that this parameter estimate is statistically significant at the 5% level. Similarly, the Poisson estimates reveal a positive relationship with age, yielding a parameter estimate of 0.017. This translates into a statistically significantly higher mean monthly hospital expenditure of $6.94 per year of age. Comparing these estimates to other studies is inherently challenging due to differences in econometric specifications, healthcare systems, the scope of healthcare expenditures considered, and reporting methods. Nevertheless, our findings closely align with Seshamani and Gray (2004b), who estimate that hospital expenditures in England increase by approximately $6.88 per month between ages 65 and 70.^17^ Similarly, Hyun et al. (2016) report a monthly increase of $7.08 in inpatient hospital expenditures for individuals aged 60–64 compared with those aged 65–70.^18^

In columns four to six, the Red Herring parameterization introduces time‐to‐death (TTD) as a regressor. Consistent with the existing literature (Seshamani and Gray 2004b), TTD exhibits large, negative parameter estimates in both the Probit and Poisson models. Consequently, in the full two‐part model, each additional year further from death is associated with a $177.00 reduction in average monthly hospital expenditures. The age coefficients decrease across all three Red Herring columns, corroborating the findings of Zweifel et al. (1999) that the age effect diminishes upon accounting for time‐to‐death. The age parameter estimates, although reduced, remain statistically significant, similar to existing literature using larger datasets (Seshamani and Gray 2004a; Atella and Conti 2014). Additionally, the introduction of time‐to‐death as a regressor increases the log‐likelihood function, indicating improved model fit.

The Steepening parameterization in columns seven through nine incorporates time and the age‐time interaction into the Naïve specification. Notably, the coefficient for the age‐time interaction, estimated at 0.033 in the Probit model, suggests that the probability of incurring hospital expenditures increases more rapidly with age in later calendar years than in earlier years. Conversely, in the Poisson model, the negative coefficient of −0.011 reflects a counter‐Steepening effect, indicating that among those who do incur hospital expenditures within a month, expenditure growth decreases over time for older individuals, a pattern observed in Figure 1e. Meanwhile, simply interpreting the parameter estimates can be misleading, especially for nonlinear functions and interaction effects, thus marginal associations are preferred for the direction and size of effects (see Supporting Information S1: Online Appendix B.1 for the calculation of marginal associations). The marginal association estimate, accounting for the full set of the Probit and Poisson coefficients, results in a net increase of $11.44 in expenditures. Thus, each additional year of age amplifies the increase in hospital spending over time by $11.44 on average. Gregersen (2014) report a comparable figure for Norwegians aged 50–54 in 2014, amounting to $11.05.^19^ The inclusion of Steepening parameters increases the log‐likelihood function, though to a lesser extent than time‐to‐death.

In the final three columns of Table 1, we present our primary results, examining the Red Herring Hypothesis and the Steepening Hypothesis simultaneously using the new parameterization specified in Equation (5), which incorporates time‐to‐death, time, and age interacted with time as regressors. Compared to the estimates under the Red Herring parameterization, the inclusion of the Steepening parameters (time and the age‐time interaction) produces minimal changes in the estimates for age and time‐to‐death. The age effects remain small, while the time‐to‐death effects are substantial. When comparing the Steepening parameterization estimates to those from the Joint Model, we observe that the introduction of time‐to‐death effects greatly influences the parameter estimates. Notably, the interaction effect between age and time in the Poisson component changes from a negative to a positive value of 0.003, suggesting a Steepening effect where mean hospital expenditures increase more rapidly for older individuals. The overall effect in the Joint Model, $E_{\psi }$, is slightly reduced to $9.15.^20^ Incorporating both time‐to‐death from the Red Herring Hypothesis and the Steepening parameters yields the highest log‐likelihood value, indicating the best fit to data. A complete regression table, including additional controls and marginal effects in the Probit and Poisson components, is provided in Supporting Information S1: Online Appendix C.2. We analyze the impact of the Red Herring and the Steepening Hypotheses on aggregate hospital expenditures in Section 4.3.

**Hypotheses Tests**

In Table 1, the full‐population parameter estimates are all highly statistically significant at the 5% level, as shown by the *p*‐values in square brackets.^21^ Large Z‐scores in curly brackets confirm precise parameter estimates, especially in the Poisson model. For each single parameter, a test of a zero‐effect null hypotheses on time‐to‐death, and the interaction of age with time, can all be rejected irrespective of the model specification. Meanwhile, we are also interested in testing multiple parameter simultaneously, that is, in both parts of our two‐part model as well as the joint test of both the Red Herring and the Steepening parametrizations. To test whether multiple parameters are simultaneously zero in the full two‐part model in Equation (1), Table 2 tests the three hypotheses on the parameter estimates from Equation (6) using the Wald test and the Bayes *p*‐values.

**TABLE 2:** Hypothesis test of parameters.

		Wald test		Bayesian	
	#Res.	Test statistic	*p*‐value	Bayes factor	*p*‐value
$H_{0}^{1}$	6	106,217	0.000	−84,883,437	0.000
$H_{0}^{2}$	6	20,762	0.000	−2,349,166	0.000
$H_{0}^{3}$	2	39,247	0.000	−84,613,510	0.000

*Note:* Hypothesis tests and *p*‐values. #Res. refers to the number of parameter restrictions.

The first hypothesis, $H_{0}^{1}$, maintains the Naïve model as the true specification; the second hypothesis, $H_{0}^{2}$, proposes a strict Red Herring setup with only time‐to‐death as regressor; and, lastly, $H_{0}^{3}$ posits the Steepening specification, excluding time‐to‐death as a regressor, as discussed in Section 2.2. Each Wald‐test and Bayesian assessment returns a *p*‐value of zero, providing strong evidence against $H_{0}^{1}$, $H_{0}^{2}$, and $H_{0}^{3}$, all of which are rejected. Thus, we fail to reject the alternative hypothesis that all parameters are nonzero. This leaves the Joint Model as the preferred specification. We draw similar conclusions for Wald tests of the marginal effects (Supporting Information S1: Online Appendix B.2) and when analyzing the female and male populations separately, as reported in Supporting Information S1: Online Appendix C.3.

### 4.1 Heterogeneity by Age and Sex

The impact of a changing population composition on hospital expenditures depends on which age and sex groups experience changes in size. This variation is particularly relevant when effect sizes differ across subpopulations, thus necessitating a precise understanding of these disparities. Existing studies provide valuable insights into age‐related differences: Seshamani and Gray (2004a) and Atella and Conti (2014) explore the Red Herring Hypothesis, while Kollerup et al. (2022) and Gregersen (2014) examine age differences in the context of the Steepening Hypothesis. However, these analyses are limited, providing only partial evidence, as they consider each hypothesis in isolation, thus overlooking their potential coexistence. Formal statistical tests remain absent from the literature for coexistence as well as group‐differences. Finally, heterogeneity by sex has received minimal attention in both strands of literature (Kallestrup‐Lamb et al. 2024).

In this section, we present and implement a method to address these limitations. Traditionally, the effects of age‐groups and sex are modeled using binary indicators, leading to complex, high‐dimensional models that are difficult to interpret. We take a more parsimonious approach by analyzing each group separately, which allows for greater flexibility as each group has distinct parameter estimates for controls and key variables. Additionally, estimating separate parameters enables the use of Welch tests (Welch 1947) to assess whether aging patterns—measured by age, time‐to‐death, and Steepening—significantly differ across age and sex subgroups. While the Steepening Hypothesis is classically formulated as a global statement, that is, that the growth rate of healthcare expenditures increases across the entire age range, we argue that estimating age‐specific patterns can provide more nuanced insight into how and where Steepening occurs. By restricting estimation to narrower age bands, we allow for the possibility that the rate of expenditure growth is not uniform over the age‐distribution. From a policy perspective, these insights helps identify which age‐sex subpopulations may exhibit the strongest hospital expenditure acceleration under Red Herring and Steepening effects.

Table 3 presents these estimates. We consider sex‐specific estimates from our Joint Model across 10‐year age groups, including those aged 90 and above. We report the full marginal effects of the two‐part model, $E_{\psi }$, accounting for the competing contributions of the Probit and Poisson components via the Delta Method.^22^ Our estimates confirm findings from the literature that consider each hypothesis separately for hospital expenditures as the outcome.^23^ The time‐to‐death (TTD) effects generally increase in absolute magnitude across age groups, indicating that end‐of‐life hospital expenditures are highest for older individuals (Seshamani and Gray 2004b). Additionally, Steepening effects (Age · Time) are statistically significant for all age groups and increase with age (Gregersen 2014). Our approach, however, also provides several new insights, made possible by accounting for coexistent hypotheses and subgroup analyses by age and sex. First, we find simultaneous Red Herring and Steepening effects for all age and sex groups, as shown in Table 3 and confirmed by Wald tests and Bayesian *p*‐values (see Supporting Information S1: Table A13, Online Appendix C.4), suggesting that evaluating each hypothesis in isolation may be misleading. Second, time‐to‐death and Steepening effects are generally statistically different across age groups, as implied by Welch tests (Supporting Information S1: Table A15, Online Appendix C.4), demonstrating that population change affects each subgroup differently. Third, the magnitude of the effects differ by sex and are statistically significantly different from each other (Supporting Information S1: Table A16, Online Appendix C.4), with males generally exhibiting higher time‐to‐death and Steepening effects than females. Fourth, not all age groups experience Steepening effects. Among females aged 30–40 and individuals aged 90 and above, the Steepening coefficient is negative, suggesting that hospital expenditure growth is lower among older individuals in these groups. Instead, Steepening effects are most pronounced between ages 50 and 80, indicating a steepening of the age‐profile in this interval.

**TABLE 3:** Marginal association, $E_{\psi }$, by age groups.

Age group	[30; 40)		[40; 50)		[50; 60)		[60; 70)		[70; 80)		[80; 90)		90+	
Sex	F	M	F	M	F	M	F	M	F	M	F	M	F	M
Age	−5.34	1.71	1.92	3.72	2.79	5.76	4.98	9.23	3.15	−0.76	−6.33	−17.29	−18.02	−26.66
	(0.560)	(0.296)	(0.215)	(0.621)	(0.386)	(0.895)	(0.407)	(1.046)	(0.443)	(0.378)	(0.264)	(1.355)	(0.587)	(0.808)
	[0.000]	[0.000]	[0.000]	[0.000]	[0.000]	[0.000]	[0.000]	[0.000]	[0.000]	[0.044]	[0.000]	[0.000]	[0.000]	[0.000]
	{−10.56}	{7.08}	{7.84}	{7.85}	{8.78}	{9.10}	{10.49}	{10.81}	{10.88}	{14.26}	{−22.05}	{−29.85}	{−43.74}	{−73.03}
TTD	−92.48	−34.00	−91.99	−61.06	−124.18	−117.10	−163.51	−193.40	−217.17	−278.30	−215.68	−305.32	−176.62	−304.04
	(8.377)	(4.461)	(8.985)	(9.203)	(13.674)	(19.229)	(22.332)	(32.393)	(32.398)	(39.701)	(30.377)	(32.730)	(15.762)	(13.984)
	[0.000]	[0.000]	[0.000]	[0.000]	[0.000]	[0.000]	[0.000]	[0.000]	[0.000]	[0.000]	[0.000]	[0.000]	[0.000]	[0.000]
	{−11.29}	{−9.48}	{−10.05}	{−9.22}	{−11.02}	{−10.37}	{−12.59}	{−12.20}	{−15.48}	{−17.44}	{−20.07}	{−27.79}	{−40.44}	{−70.48}
Age⋅Time	−1.92	2.55	5.49	5.88	8.23	10.11	11.37	17.90	12.80	11.54	5.77	1.23	−7.65	−8.56
	(0.704)	(0.070)	(0.351)	(0.607)	(0.624)	(1.166)	(0.706)	(1.494)	(1.161)	(0.489)	(1.002)	(0.460)	(0.164)	(0.495)
	[0.006]	[0.000]	[0.000]	[0.000]	[0.000]	[0.000]	[0.000]	[0.000]	[0.000]	[0.000]	[0.000]	[0.007]	[0.000]	[0.000]
	{3.25}	{8.14}	{8.72}	{8.16}	{9.79}	{9.29}	{11.20}	{11.18}	{14.49}	{16.63}	{16.50}	{25.39}	{−51.07}	{−9.11}
Controls	Yes	Yes	Yes	Yes	Yes	Yes	Yes	Yes	Yes	Yes	Yes	Yes	Yes	Yes
Num. Obs.	1,036,274	1,056,546	1,107,349	1,133,078	1,041,301	1,049,897	912,058	883,170	610,754	520,022	337,275	204,593	80,406	27,277

*Note:* Dependent variable is monthly somatic hospital expenditures in 2020 USD, estimated separately by age‐sex group. The table reports the average marginal effect on expected monthly hospital expenditures from the full two‐part model. Standard errors in round parentheses (); *p*‐values in square brackets [ ]; Z‐scores in curly brackets { }. TTD is short for time‐to‐death. Controls refer to an intercept, month dummies, time, and ${\overset{ˇ}{X}}^{*}$.

To further disentangle the relative contributions of the Probit and Poisson components, we replicate the analysis from Table 3 separately for each margin in Supporting Information S1: Online Appendix C.4, Table A12. Time‐to‐death coefficients are negative at both margins, confirming that hospital utilization probability and expenditure amounts decline with distance from death, though magnitudes vary by age group. For Steepening effects, the extensive margin shows positive effects across nearly all groups (except females 90+), strongest at ages 50–80. The intensive margin reveals a more complex pattern: expenditures grow more slowly for ages 30–40 and 80+, particularly for males 90+ (−$26.41), while remaining positive between ages 40–80 for females and 40–70 for males. This suggests that while Steepening drives expenditure growth among hospitalized individuals in middle‐to‐older age ranges, the effect is contained or reversed at the oldest ages, partially mitigating fiscal pressures from population aging.

### 4.2 Heterogeneity by Time‐To‐Death and Sex

The decomposition proposed in Section 2.3 suggests, mathematically, that a new interaction effect combining the Red Herring and the Steepening Hypotheses might exist. To assess the empirical standpoint on this, we estimate our Joint Model for three distinct time‐to‐death groups. If the Steepening effect differs across time‐to‐death groups, there is evidence favoring this new Red Herring‐Steepening interaction. The decomposition does not address differences by sex, but since Section 4.1 found marked differences in Steepening effects by sex, we conduct this analysis for each sex separately. The three time‐to‐death groups are: less than 1.5 years to death (around the kink in the time‐to‐death plot in Figure 1b), between 1.5 and 4 years, and more than 4 years to death, respectively.

Table 4 presents the marginal associations of the two‐part model, $E_{\psi }$. Comparing our estimates of time‐to‐death effects with the existing literature that focuses exclusively on the Red Herring hypothesis without addressing the Steepening Hypothesis, our findings corroborate those of previous studies (Seshamani and Gray 2004a). Specifically, the time‐to‐death effect exhibits a nonlinear relationship with proximity to death, characterized by escalating expenditures as death approaches. For individuals closest to death, our time‐to‐death effects are substantial: −$2254.46 for females and −$2928.96 for males. These effects diminish considerably to −$113.99 and −$146.07, respectively, for those with 1.5–4 years remaining until death. For individuals with more than 4 years to death, the time‐to‐death parameter cannot be estimated due to insufficient variation in time‐to‐death within this subsample.

**TABLE 4:** Marginal association, $E_{\psi }$, by time‐to‐death groups.

Sex	TTD∈[0; 1.5)		TTD∈[1.5; 4)		TTD > 4	
	F	M	F	M	F	M
Age	−59.40	−36.57	−14.93	−7.68	2.87	4.97
	(4.623)	(2.682)	(1.313)	(1.000)	(1.014)	(1.490)
	[0.000]	[0.000]	[0.000]	[0.000]	[0.005]	[0.001]
	{−38.23}	{−39.87}	{−28.54}	{29.44}	{4.69}	{4.59}
TTD	−2254.46	−2928.96	−113.99	−146.07	—	—
	(192.133)	(241.446)	(13.018)	(15.171)	(−)	(−)
	[0.000]	[0.000]	[0.000]	[0.000]	[−]	[−]
	{−38.48}	{−43.76}	{−27.03}	{−29.70}	{−}	{−}
Age ⋅ Time	−5.84	40.06	3.88	14.74	6.92	8.11
	(0.735)	(2.953)	(0.062)	(0.376)	(0.505)	(1.221)
	[0.000]	[0.000]	[0.000]	[0.000]	[0.000]	[0.000]
	{5.72}	{44.10}	{28.79}	{30.57}	{4.86}	{4.74}
Controls	Yes	Yes	Yes	Yes	Yes	Yes
Num. Obs.	115,256	110,539	189,158	185,651	4,821,003	4,578,393

*Note:* Dependent variable is monthly somatic hospital expenditures in 2020 USD, estimated separately by time‐to‐death and sex group. The table reports the average marginal effect on expected monthly hospital expenditures from the full two‐part model. Standard errors in round parentheses (); *p*‐values in square brackets [ ]; Z‐scores in curly brackets { }. TTD is short for time‐to‐death. Controls include an intercept, month dummies, time, and ${\overset{ˇ}{X}}^{*}$.

Variability in the Steepening effects among individuals with different time‐to‐death has not been previously examined in the literature. Large, positive Steepening effects are concerning for both individuals still far from death and those nearing the end of life. If healthcare expenditures steepen in the years just before death, future aggregate hospital expenditures could increase greatly, especially if the number of individuals near death increase as seen, for example, with the aging of the baby boomer cohorts. For females with less than 1.5 years to death, the negative Steepening effect of −$5.84 indicates that older individuals are experiencing flatter (or declining) spending trajectories over time, thus reducing the expenditure pressure of expanding older populations. Meanwhile, for the remaining two time‐to‐death groups, the Steepening effect is positive, with magnitudes of $3.88 and $6.92, respectively. Males, however, have exhibited a substantially larger degree of Steepening across all three time‐to‐death groups: $40.06, $14.74, and $8.11, respectively. Consequently, a surge in the older population may present a markedly greater economic concern for males than it does for females in the last years of life. Welch tests of equal Steepening parameters across age groups reject the null hypothesis of equal parameters and support the alternative that parameter estimates differ significantly for both females and males (see Supporting Information S1: Table A18, Online Appendix C.5). This heterogeneity provides empirical support for the Red Herring‐Steepening interaction. Thus, the Red Herring and Steepening Hypotheses demonstrate specific nuances not previously considered in the literature. Table 4 yields several additional findings. First, we accept the alternative hypothesis of coexistence of the Red Herring and Steepening effects within each time‐to‐death group for both sexes (Wald tests and Bayes *p*‐values, Supporting Information S1: Table A19 and Table A20, Online Appendix C.5). Second, the Steepening effects differ significantly between females and males for each time‐to‐death group (Supporting Information S1: Table A19, Online Appendix C.5). These results demonstrate that hospital expenditure patterns are substantially influenced by the sex composition of population changes.

To assess margin‐specific contributions, we estimate marginal associations for the Probit and Poisson models in Panels B and C, Supporting Information S1: Table A17, Appendix C.5. At the extensive margin, all subgroups show positive Steepening effects though the effect approaches zero for females nearest death. At the intensive margin, Steepening is negative for both sexes with 1.5–4 years to death and for females with < 1.5 years −$13.09, indicating declining monthly expenditures among those approaching death. Males with < 1.5 years to death are an exception ($14.73). Notably, groups with > 4 years to death show insignificant intensive‐margin Steepening, suggesting age‐specific expenditure growth occurs predominantly near end‐of‐life. Overall, the Steepening effects in Table 4 are primarily driven by the extensive margin.

### 4.3 Decomposition Results

In this section, we apply the decomposition method introduced in Section 2.3 to the full dataset described in Section 3, with monthly hospital expenditures aggregated to annual amounts for each individual. Table 5 presents the results of the analysis for the years $t_{1}$ = 2017 and $t_{2}$ = 2002. As shown in the first row, total hospital expenditures increased by $3594.1 million from 2002 to 2017. For the two‐component decomposition in Panel A, only 20.8% of this increase was attributed to population changes, while a substantial 79.2% resulted from intertemporal changes in mean hospital expenditures. We also observe notable differences by sex. For females, just 12.6% of the expenditure increase was attributable to population changes, whereas for males, this figure was nearly three times larger at 30.9%. Conversely, changes in mean hospital expenditures played a more significant role for females, accounting for 87.4% of the total increase.

**TABLE 5:** Decomposition of hospital care expenditure differences between 2002 and 2017.

		Panel A:				Panel B:							
		Two‐component decomposition				Four‐component decomposition							
	Total	Pop. Change		Hc. Change		Naïve		RH error		Steepening		RH steep.	
	$mio.	$mio.	%	$mio.	%	$mio.	%	$mio.	%	$mio.	%	$mio.	%
All	3594.1	747.6	20.8	2846.5	79.2	1440.6	40.1	−693.0	−19.3	2153.5	59.9	693.0	19.3
Female	1740.1	218.9	12.6	1521.2	87.4	528.1	30.4	−309.2	−17.8	1212.0	69.6	309.2	17.8
Male	1854.0	573.2	30.9	1280.8	69.1	954.0	51.5	−380.7	−20.5	900.0	48.5	380.7	20.5

*Note:* Pop. Change is the change due to population changes. Hc. Change is the change due to changes in mean somatic hospital expenditures. RH Error is the Red Herring error. RH Steep. is the change in hospital expenditures due to intertemporal changes in the Red Herring related estimation error. Estimates are based on the entire Danish population in 2002 and 2017. Amounts in 2020 USD.

Turning to the four‐component decomposition in Panel B, the Naïve component exerted an upward pressure on expenditure growth, accounting for 40.8% of the change. Meanwhile, the Red Herring error had the expected mitigating effect, reducing the increase by 19.3%, aligning closely with previous estimates that do not account for Steepening (Breyer and Lorenz 2021). While the Red Herring error exhibited little variation by sex, the Naïve age component was more pronounced among males, exceeding half of their total hospital expenditure growth from 2002 to 2017. Steepening effects, representing intertemporal changes in hospital expenditures, contributed significantly to expenditure growth, accounting for 59.9% of the total increase, three times larger than the Red Herring error in absolute terms. This effect was particularly pronounced for females, where it explained 69.6% of the change. In the final two columns of Table 5, we examine the newly identified Red Herring‐Steepening effect, derived in Section 2.3 and empirically validated in Section 4.2. This effect contributed 19.3% to the total hospital expenditure increase, roughly one‐fifth of the total growth. Its magnitude was nearly identical for both sexes, underscoring its importance in explaining rising hospital expenditures. Crucially, the Red Herring‐Steepening component was of the same absolute size as the time‐to‐death prediction error (19.3%), reinforcing the significance of this phenomenon. A robustness analysis using the years $t_{1}=2015$ and $t_{2}=2005$ confirms our main decomposition estimates presented in Table 5. The robustness estimates exhibit minimal deviation from the primary findings, as detailed in Supporting Information S1: Online Appendix C.6.

## 5 Discussion

Several potential mechanisms may underlie the observed Steepening effect and its interaction with the Red Herring Hypothesis, including technological advancements in hospital care (Chandra and Skinner 2012; Laudicella et al. 2022), and increasing multimorbidity prevalence among older individuals (Rosella et al. 2018). However, shifts in hospital utilization and prices also explain the differences in hospital expenditures (Dieleman et al. 2025). Our analysis indicates that the extensive margin—measured by the number of months with hospital care utilization—plays a key role in the Steepening pattern, as older individuals are increasingly likely to use hospital expenditures within a given month as they grow older. This suggests that both treatment practice and the demand for care may be underlying mechanisms inducing Steepening. Prior research suggests that the Red Herring effect appears to be mediated by health status (Seshamani and Gray 2004b; Polder et al. 2006; Howdon and Rice 2018; Moore et al. 2017; Maynou et al. 2023). Indeed, Carreras et al. (2018) found that time‐to‐death becomes statistically insignificant when severity‐based health status measures are incorporated into the analysis. While our findings illuminate the broader implications of traditional population aging metrics (age, time‐to‐death, and steepening), further research disentangling these health drivers of both Red Herring and Steepening effects is warranted, particularly with regard to sex‐specific patterns and differences across healthcare settings.

A longstanding concern in the Red Herring literature relates to the plausible endogeneity between hospital expenditures and time‐to‐death (Salas and Raftery 2001), which potentially influences our analysis. If hospital services successfully prolong life, then higher expenditures may be both a consequence and a cause of longer time‐to‐death. Several studies have addressed this issue partially using instrumental variable approaches (Felder et al. 2010; Karlsson and Klohn 2011; Howdon and Rice 2018), with parental age at death serving as an instrument for time‐to‐death (Kolodziejczyk 2020; Costa‐Font and Vilaplana‐Prieto 2020). Others have treated past healthcare expenditures and time‐to‐death as predetermined exogenous factors influencing current time‐to‐death (Felder et al. 2010). However, the direct causal effect of hospital expenditures on time‐to‐death remains largely unexamined in this literature. A more rigorous treatment of endogeneity therefore represents an important direction for future work on both the Red Herring and Steepening hypotheses. Promising avenues include exploiting exogenous variation in healthcare access or policy reforms, developing dynamic survival–expenditure models that explicitly reflect the temporal ordering from period‐t spending to future mortality, or leveraging quasi‐experimental designs based on unexpected deaths where timing is plausibly exogenous to recent utilization.

## 6 Conclusion

The healthcare expenditure effects of an increasing number of older individuals remain one of the most pressing challenges for healthcare systems globally (K. Christensen et al. 2009). Previous research has addressed this phenomenon through two distinct concepts: the Red Herring Hypothesis (Zweifel et al. 1999; Seshamani and Gray 2004b) and the Steepening Hypothesis (Buchner and Wasem 2006). This paper makes a significant contribution by investigating both hypotheses concurrently. Through a novel model parameterization and analysis of comprehensive register‐based data capturing monthly hospital expenditures for the entire Danish population aged 30+, we provide robust evidence for the concurrence of both mechanisms. Results from rigorous statistical testing demonstrate that neither hypothesis should be rejected independently, nor should their joint effect. We find substantial heterogeneity in the Red Herring and Steepening effects across age groups and time‐to‐death categories. As a novel contribution to this literature, our sex‐stratified analysis demonstrates that the effects of population change differ across sexes, generally being more pronounced for males than females,

When analyzed together, the magnitude of both time‐to‐death and Steepening effects diminishes compared to when each is considered in isolation, suggesting important interactions between these phenomena at the three dimensional intersection of age, time‐to‐death, and time. Nevertheless, the empirical effects attributable to each hypothesis remain substantial. A novel decomposition analysis shows that from 2002 to 2017, Steepening effects accounted for 59.9% of the total increase in hospital expenditures, three times the size of the mitigating Red Herring effect. Furthermore, the decomposition identifies a previously unrecognized Red Herring‐Steepening interaction effect, which contributed an additional 19.3% to the total expenditure increase. Overall, our findings emphasize that when the Red Herring and Steepening Hypotheses are analyzed together, their interaction reveals a more complex and concerning picture of hospital expenditure dynamics than much of the existing literature suggests.

## Conflicts of Interest

The authors declare no conflicts of interest.

## Supporting information


Supporting Information S1

## Data Availability

The data used for this analysis are available upon submission of an application to Statistics Denmark, https://www.dst.dk/en. Restrictions may apply to the availability of data. Upon request, the authors will assist in replicating study results.

## Endnotes

^1^
We employ quasi‐maximum likelihood estimation with robust standard errors, which provides consistent parameter estimates and valid inference even under distributional misspecification, requiring only correct specification of the conditional mean function. Hence, the choice of the distribution of the second part is of lesser importance (White 1982; Gourieroux et al. 1984). We conduct robustness estimates against the conditional mean function specification.

^2^
Maynou et al. (2023) also use average marginal effects, as defined by Norton and Dowd (2018), to analyze hospital expenditures. However, their model does not explicitly include time‐to‐death or Steepening variables; instead, it controls for timing via month‐of‐death dummies.

^3^
Conceptually, the two‐part model in Equation (1) aligns with the approach proposed by Heckman (1976), but it diverges significantly in its handling of zero hospital expenditures, treating them as actual zeros rather than censored observations. Moreover, Seshamani and Gray (2004a) underscore the problem of multicollinearity in the analysis of hospital expenditures arising from the inclusion of the Inverse Mills Ratio in the second stage of the Heckman (1976) model, advocating for the two‐part model as a more robust alternative for analyzing hospital expenditures.

^4^
We consider one of several types of Red Herring Hypotheses, as discussed by, e.g., Breyer and Lorenz (2021), which is suitable for our dataset and research question.

^5^
In practice, time‐to‐death is set to four and the binary indicator to one, if an individual has not died through our period of observation, described later in the data section.

^6^
Analyzing the Steepening Hypothesis in relative terms (i.e., by comparing percentage growth across age groups) could provide complementary insights. A relative perspective helps to standardize across different baseline expenditure levels, identify age groups where costs are accelerating fastest, inform targeted health policy responses, and integrate into macroeconomic models considering growth rates in, for example, GDP and other macroeconomic outputs. Meanwhile, accounting practices and healthcare budgets are typically considered in absolute terms.

^7^
Our specification of the variables related to the Steepening Hypothesis follows the most comprehensive discussion of the coexistent Red Herring and Steepening Hypotheses by Gregersen (2014), where calendar age and time are considered related to Steepening while age and time‐to‐death are the central factors for the Red Herring Hypothesis. We, however, acknowledge that many empirical analyses of the Red Herring Hypothesis and Naïve models control for calendar time in their regressions, for example, Zweifel et al. (1999) and Seshamani and Gray (2004a). Yet, instances of the Naïve model and Red Herring models without changes in hospital expenditures over time, that is, zero growth rates, have also been considered by, for example, van Baal and Wong (2012) and for projections of healthcare expenditures (Polder et al. 2006; Bjørner and Arnberg 2012; Geue et al. 2014).

^8^
The indicator accounts for the fact that people in their final year have fewer than 12 full months to consume hospital, which reduces the total expenditures in that period.

^9^
The Wald test tends to perform similarly to the Likelihood Ratio tests and the Score test in large samples, but the Wald test is often the simplest to compute and interpret (Engle 1984). A limitation of our testing procedure is that we cannot combine two‐sided and one‐sided tests. Specifically, the Steepening Hypothesis posits that the marginal effect of the age‐time interaction is positive, that is, a one‐sided hypothesis.

^10^
With our large sample (638M observations, 10M in regression subsamples), conventional significance thresholds may identify statistically significant effects that are economically negligible. The actual difference required to register as statistically significant at the 5% (or lower) level remains very small, potentially leading to over‐interpretation of parameter estimates based solely on *p*‐values. As a rescaled alternative to *p*‐values, Z‐scores quantify the precision of a single parameter estimate under the null of a zero‐parameter size.

^11^
In large samples, the exponential of the Schwarz criterion, $S={\hat{\ell }}_{H_{0}}-{\hat{\ell }}_{H_{A}}-\frac{1}{2}\left(\left|\theta_{0}|-|\theta_{A}\right|\right)\log\left(N\right)$, approximates the Bayes factor well (Kass and Raftery 1995). $\hat{\ell }$ denotes maximized log‐likelihood, |θ| the number of parameters, and N the number of observations. Prior odds, $\mathrm{Pr}\left(H_{0}\right)/\mathrm{Pr}\left(H_{A}\right)$, are set at 20%, but our results remain robust to prior beliefs ranging from $\mathrm{Pr}\left(H_{0}\right)=0.01$ to $\mathrm{Pr}\left(H_{0}\right)=0.99$.

^12^
Other Naïve models examining the impact of the Red Herring Hypothesis on aggregate expenditures also exclude time trends in expenditures (see Polder et al. (2006); Bjørner and Arnberg (2012); Geue et al. (2014)), making our approach comparable.

^13^
If a hospital stay extends across multiple months, expenditures are distributed proportionally based on the number of days spent per month. Hospital expenditures are inflated to 2020 levels in DKK using the consumer price index from Statistics Denmark (2024) and converted to 2020 USD using the 2020 exchange rate of 1/6.534 from Danmarks Nationalbank (2024).

^14^
Specifically for Kollerup et al. (2022), that is, after accounting for the inclusion of additional expenditure types in their analysis. We do not report these comparisons in this paper for brevity.

^15^
For individuals alive beyond 2021, we cannot observe their exact time‐to‐death if it exceeds four years as their death is right‐censored.

^16^
The project, no. 704863, was carried out under the Agreement 2016‐051‐000001, between The Danish Data Protection Agency and 1447 and has been approved by Statistics Denmark. There is no Danish institutional review board for studies based on standard register data at Statistics Denmark. Individual‐level information from the administrative registers is confidential to the Danish Administrative Procedures (Section 27) and the Danish Criminal Code (Section 152), and data cannot be made publicly available. The data used for this analysis are available upon submission of an application to Statistics Denmark, https://www.dst.dk/en. Restrictions may apply to the availability of data. Upon request, the authors will assist in replicating study results.

^17^
This calculation is based on a £ 250 increase over five years, converted to a monthly rate using the January 1999 exchange rate of $/£1.65 (Bank of England 2025): $6.88 per month = $/£1.65. £250/5 years/12 months per year.

^18^
This is derived from a 484,000 KRW difference in inpatient expenditures in 2012, converted to USD using the January 2012 exchange rate of 1 USD = 1140 KRW (Federal Reserve (US) 2025a) $7.08 per month = $/KRW1/1140. KRW 484,000/5 years/12 months per year.

^19^
An average annual hospital expenditure Steepening effect of €183.3 for 50–54‐year‐olds corresponds to a monthly rate of $11.05 = $€1/1.38 ⋅ 183.3€/12 months, using the exchange rate of 1.38 in January 2014 (Federal Reserve (US) 2025b).

^20^
The full marginal association of $9.15 is lower than the $11.44 observed under the Steepening parameterization, despite the positive shift in the age‐time interaction parameter in both the Probit and Poisson parts. This reduction is due to the inclusion of additional regressors and the associated changes in other regression coefficients, which affect the estimated effect.

^21^
We expect highly significant estimates due to our huge sample size.

^22^
Specifically, the change in hospital expenditures with respect to a variable, x, is the expectation of ∂ψ/∂x=∂F/∂x⋅g+F⋅∂g/∂x across individuals for x being age or time‐to‐death. For the age‐time interaction, accounting for the full influence of parameter estimates on age, time, and the age‐time interaction is critical, as derived in Supporting Information S1: Online Appendix B.1.

^23^
For long‐term care expenditures Karlsson and Klohn (2014) finds a different pattern with a few instances of lower expenditures among older individuals. Moreover, in a non‐regression based descriptive statistic, Tanuseputro et al. (2015) find hospital expenditures to be larger in the last single year of life for individuals above age 75.

## Appendix A A Model for Decomposing Changes in Aggregate Hospital Expenditures

This section outlines our approach to decomposing changes in aggregate hospital expenditures between $t_{2}=2002$ and $t_{1}=2017$ into distinct components. These include: (i) the Naïve Model, which assumes expenditures vary only by age; (ii) the Red Herring Error, capturing the role of time‐to‐death in expenditure variation; (iii) the Steepening Hypothesis, which accounts for intertemporal changes in age‐specific hospital expenditures; and (iv) a newly identified component, the Red Herring‐Steepening effect, which isolates Steepening effects specifically within the last years of life.

Following van Baal and Wong (2012), let annual aggregate hospital expenditures, H, in year, T, be expressed as the sum of mean hospital expenditures, $\bar{H}$, across groups of individuals at age, A, and time‐to‐death, TTD, categories

(A1)
$$H_{T}=\sum_{A}\sum_{TTD}N_{T,A,TTD}{\bar{H}}_{T,A,TTD},$$

where $N_{T,A,TTD}$ presents the number of individuals of age. We consider ages as integers from 30 to 110+, with time‐to‐death rounded to the first decimal. A key advantage of this formulation is its reliance on directly observable data without imposing restrictive functional assumptions on the relationships between expenditures, age, time‐to‐death, or calendar time.

The change in aggregate hospital expenditures from $t_{2}$ to $t_{1}$ can be decomposed as follows:

(A2)
$$\begin{array}{cc} & \Delta_{t_{1},t_{2}}^{H}:=H_{t_{1}}-H_{t_{2}}\\ & =\sum_{A}\sum_{TTD}N_{t_{1},A,TTD}{\bar{H}}_{t_{1},A,TTD}-N_{t_{2},A,TTD}{\bar{H}}_{t_{2},A,TTD}\\ & =\underset{\text{Population}\;\text{Change}}{\underbrace{\sum_{A}\sum_{TTD}\left[N_{t_{1},A,TTD}-N_{t_{2},A,TTD}\right]{\bar{H}}_{t_{1},A,TTD}}}+\underset{\text{Expenditure}\;\text{Change}}{\underbrace{\sum_{A}\sum_{TTD}\left[{\bar{H}}_{t_{1},A,TTD}-{\bar{H}}_{t_{2},A,TTD}\right]N_{t_{2},A,TTD}}}. \end{array}$$

The Population Change component isolates the impact of demographic shifts, $N_{t_{1},A,TTD}-N_{t_{2},A,TTD}$, while holding mean expenditures constant at their $t_{1}$ level, ${\bar{H}}_{t_{1},A,TTD}$. If expenditures were unchanged between $t_{1}$ and $t_{2}$, this term would fully explain the variation in total spending. However, when mean expenditures also evolve over time, individuals in $t_{2}$ $\left(N_{t_{2},A,TTD}\right)$ exhibit different levels of hospital consumption, generating the Expenditure Change component, which captures shifts in per capita spending, ${\bar{H}}_{t_{1},A,TTD}-{\bar{H}}_{t_{2},A,TTD}$.

While this two‐part decomposition is attractive, it does not explicitly separate the contributions of the Red Herring Hypothesis and the Steepening Hypothesis. No prior work in the literature has provided such a decomposition. The Red Herring Hypothesis posits that hospital expenditures vary not just by age but also by proximity to death. If this were ignored, aggregate projections of hospital expenditures would mistakenly attribute time‐to‐death effects to aging alone. To disentangle these effects, we expand the Population Change term using a time‐to‐death‐insensitive expenditure measure, ${\bar{H}}_{T,A}$, yielding

(A3)
$$\begin{array}{cc} & \underset{\text{Population}\;\text{Change}}{\underbrace{\sum_{A}\sum_{TTD}\left[N_{t_{1},A,TTD}-N_{t_{2},A,TTD}\right]{\bar{H}}_{t_{1},A,TTD}}}\\ & =\sum_{A}\sum_{TTD}\left[N_{t_{1},A,TTD}-N_{t_{2},A,TTD}\right]\left[{\bar{H}}_{t_{1},A,TTD}+{\bar{H}}_{t_{1},A}-{\bar{H}}_{t_{1},A}\right]\\ & =\underset{\text{Naïve}}{\underbrace{\sum_{A}\sum_{TTD}\left[N_{t_{1},A,TTD}-N_{t_{2},A,TTD}\right]{\bar{H}}_{t_{1},A}}}\\ & +\underset{\text{Red}\;\text{Herring}\;\text{Error}}{\underbrace{\sum_{A}\sum_{TTD}\left[N_{t_{1},A,TTD}-N_{t_{2},A,TTD}\right]\left[{\bar{H}}_{t_{1},A,TTD}-{\bar{H}}_{t_{1},A}\right]}}\\ & =\underset{\text{Naïve}}{\underbrace{\sum_{A}\left[N_{t_{1},A}-N_{t_{2},A}\right]{\bar{H}}_{t_{1},A}}}\\ & +\underset{\text{Red}\;\text{Herring}\;\text{Error}}{\underbrace{\sum_{A}\sum_{TTD}\left[N_{t_{1},A,TTD}-N_{t_{2},A,TTD}\right]\left[{\bar{H}}_{t_{1},A,TTD}-{\bar{H}}_{t_{1},A}\right]}} \end{array}$$

The Naïve component assumes expenditures depend solely on age, whereas the Red Herring Error quantifies the misattribution error, ${\bar{H}}_{t_{1},A,TTD}-{\bar{H}}_{t_{1},A}$, due to overlooking time‐to‐death effects. This decomposition provides a direct estimate of the impact of proximity to death on expenditure projections. Others have also quantified the Red Herring Error, but were unable to use exact decompositions of the effect, relying instead on predictions from regression models, see, for example, Geue et al. (2014).

In addition to examining the influence of the Red Herring hypothesis on aggregate hospital expenditures, we also aim to understand intertemporal changes in mean hospital expenditures over time. Specifically, we assess the role of the Steepening Hypothesis in driving aggregate expenditure changes. Since the Steepening Hypothesis pertains solely to age‐related changes in hospital expenditures over time, we isolate this effect by expanding the Expenditure Change component of equation (A2). To do so, we introduce age‐ and time‐specific hospital expenditures that are independent of time‐to‐death, denoted as ${\bar{H}}_{T,A}$

(A4)
$$\begin{array}{cc} & \underset{\text{Expenditure}\;\text{Change}}{\underbrace{\sum_{A}\sum_{TTD}\left[{\bar{H}}_{t_{1},A,TTD}-{\bar{H}}_{t_{2},A,TTD}\right]N_{t_{2},A,TTD}}}\\ & =\sum_{A}\sum_{TTD}\left[{\bar{H}}_{t_{1},A,TTD}+\left({\bar{H}}_{t_{1},A}-{\bar{H}}_{t_{1},A}\right)-{\bar{H}}_{t_{2},A,TTD}+\left({\bar{H}}_{t_{2},A}-{\bar{H}}_{t_{2},A}\right)\right]N_{t_{2},A,TTD}\\ & =\underset{\text{Steepening}\;\text{effect}}{\underbrace{\sum_{A}\sum_{TTD}\left[{\bar{H}}_{t_{1},A}-{\bar{H}}_{t_{2},A}\right]N_{t_{2},A,TTD}}}+\underset{\text{Red}\;\text{Herring}‐\text{Steepening}}{\underbrace{\sum_{A}\sum_{TTD}\left[\left({\bar{H}}_{t_{1},A,TTD}-{\bar{H}}_{t_{1},A}\right)-\left({\bar{H}}_{t_{2},A,TTD}-{\bar{H}}_{t_{2},A}\right)\right]N_{t_{2},A,TTD}}}\\ & =\underset{\text{Steepening}\;\text{effect}}{\underbrace{\sum_{A}\left[{\bar{H}}_{t_{1},A}-{\bar{H}}_{t_{2},A}\right]N_{t_{2},A}}}+\underset{\text{Red}\;\text{Herring}‐\text{Steepening}}{\underbrace{\sum_{A}\sum_{TTD}\left[\left({\bar{H}}_{t_{1},A,TTD}-{\bar{H}}_{t_{1},A}\right)-\left({\bar{H}}_{t_{2},A,TTD}-{\bar{H}}_{t_{2},A}\right)\right]N_{t_{2},A,TTD}}} \end{array}$$

The first term, $\left[{\bar{H}}_{t_{1},A}-{\bar{H}}_{t_{2},A}\right]N_{t_{2},A,TTD}$, captures Steepening‐related changes in hospital expenditures. It represents the number of individuals, N, at age A, experiencing an age‐specific hospital expenditure change of ${\bar{H}}_{t_{1},A}-{\bar{H}}_{t_{2},A}$ between $t_{1}$ and $t_{2}$.

The second component of equation (A4) introduces a new effect documented in this paper. This effect accounts for intertemporal changes in the time‐to‐death estimation error—specifically, how hospital expenditures evolve as time‐to‐death‐related expenditures develop over time. The term ${\bar{H}}_{t_{1},A,TTD}-{\bar{H}}_{t_{1},A}$ represents the estimation error at time $t_{1}$, while ${\bar{H}}_{t_{2},A,TTD}-{\bar{H}}_{t_{2},A}$ is its counterpart at $t_{2}$. The difference, $\left({\bar{H}}_{t_{1},A,TTD}-{\bar{H}}_{t_{1},A}\right)-\left({\bar{H}}_{t_{2},A,TTD}-{\bar{H}}_{t_{2},A}\right)$, captures the time‐dependent change in this error. We interpret this term as a Steepening‐related development in time‐to‐death estimation errors, affecting $N_{t_{2},A,TTD}$ individuals at age A with TTD years to death.

By integrating these components, we arrive at the final decomposition of aggregate hospital expenditure changes.

(A5)
$$\begin{array}{c} \Delta_{t_{1},t_{2}}^{H}:=H_{t_{1}}-H_{t_{2}} \end{array}$$

(A6)
$$\begin{array}{c} =\underset{\text{Population}\;\text{Change}}{\underbrace{\sum_{A}\sum_{TTD}\left[N_{t_{1},A,TTD}-N_{t_{2},A,TTD}\right]{\bar{H}}_{t_{1},A,TTD}}}+\underset{\text{Expenditure}\;\text{Change}}{\underbrace{\sum_{A}\sum_{TTD}\left[{\bar{H}}_{t_{1},A,TTD}-{\bar{H}}_{t_{2},A,TTD}\right]N_{t_{2},A,TTD}}} \end{array}$$

(A7)
$$\begin{array}{l} \begin{array}{cc} & =\underset{\text{Naïve}\;}{\underbrace{\sum_{A}\sum_{TTD}\left[N_{t_{1},A,TTD}-N_{t_{2},A,TTD}\right]{\bar{H}}_{t_{1},A}}}+\underset{\text{Red}\;\text{Herring}\;\text{Error}}{\underbrace{\sum_{A}\sum_{TTD}\left[N_{t_{1},A,TTD}-N_{t_{2},A,TTD}\right]\left[{\bar{H}}_{t_{1},A,TTD}-{\bar{H}}_{t_{1},A}\right]}}\\ & +\underset{\text{Steepening}\;\text{effect}}{\underbrace{\sum_{A}\sum_{TTD}\left[{\bar{H}}_{t_{1},A}-{\bar{H}}_{t_{2},A}\right]N_{t_{2},A,TTD}}}+\underset{\text{Red}\;\text{Herring}‐\text{Steepening}\;}{\underbrace{\sum_{A}\sum_{TTD}\left[\left({\bar{H}}_{t_{1},A,TTD}-{\bar{H}}_{t_{1},A}\right)-\left({\bar{H}}_{t_{2},A,TTD}-{\bar{H}}_{t_{2},A}\right)\right]N_{t_{2},A,TTD}}} \end{array} \end{array}$$

A key advantage of our decomposition framework is its exact breakdown of aggregate hospital expenditure changes into distinct components widely discussed in the literature on hospital expenditures and population aging. Namely, a Naïve component, the Red Herring Error, the Steepening Effects, and the new Red Herring‐Steepening effect. Notably, our approach does not rely on any assumptions regarding population sizes, N, or mean expenditures, $\bar{H}$. It remains robust even when certain age groups are sparsely populated (e.g., extreme ages), ensuring its applicability across different demographic structures.

### A.1 Proof of Equality Between the Red Herring Error and Red Herring‐Steepening Terms

We show that the Red Herring Error term is exactly equal to the negative of the Red Herring‐Steepening term in equation (A5), such that their sum is zero.

Let ${\bar{H}}_{T,A}=\frac{1}{N_{T,A}}\sum_{TTD}{\bar{H}}_{T,A,TTD}N_{T,A,TTD}$ denote the average hospital expenditure at age A in period T. By construction, this implies the identity: ${\bar{H}}_{T,A}N_{T,A}=\sum_{TTD}{\bar{H}}_{T,A,TTD}N_{T,A,TTD}$. Hence, aggregate hospital expenditures can be expressed as: $H_{T}=\sum_{A}\sum_{TTD}{\bar{H}}_{T,A,TTD}N_{T,A,TTD}=\sum_{A}{\bar{H}}_{T,A}N_{T,A}$.

The change in hospital expenditures between two periods $t_{1}$ and $t_{2}$ is then:

(A8)
$$\begin{array}{cc} \Delta_{t_{1},t_{2}}^{H}≔H_{t_{1}}-H_{t_{2}} & =\sum_{A}{\bar{H}}_{t_{1},A}N_{t_{1},A}-\sum_{A}{\bar{H}}_{t_{2},A}N_{t_{2},A}\\ & =\underset{\text{Naïve}\;}{\underbrace{\sum_{A}\left(N_{t_{1},A}-N_{t_{2},A}\right){\bar{H}}_{t_{1},A}}}+\underset{\text{Steepening}\;\text{effect}}{\underbrace{\sum_{A}\left({\bar{H}}_{t_{1},A}-{\bar{H}}_{t_{2},A}\right)N_{t_{2},A}}}. \end{array}$$

On the other hand, equation (A5) decomposes the same difference using a four‐part breakdown that explicitly includes terms associated with time‐to‐death (TTD). Since both decompositions are mathematically equivalent, any residual terms not appearing in equation (A8) must cancel. Specifically, the sum of the Red Herring Error term and the Red Herring‐Steepening term in equation (A5) must be zero. Therefore, the Red Herring Error term is equal to the negative of the Red Herring‐Steepening term. ■

## References

[hec70092-bib-0001] Atella, V. , and V. Conti . 2014. “The Effect of Age and Time to Death on Primary Care Costs: The Italian Experience.” Social Science & Medicine 114: 10–17. 10.1016/j.socscimed.2014.05.029. 24908170

[hec70092-bib-0002] Bank of England . 2025. A Millennium of Macroeconomic Data for the UK: U.S./U.K. Foreign Exchange Rate. Retrieved from FRED, Federal Reserve Bank of St. Louis. Original Data from the Bank of England’s Three Centuries of Macroeconomic Data Project.

[hec70092-bib-0003] Belotti, F. , P. Deb , W. G. Manning , and E. C. Norton . 2015. “Twopm: Two‐Part Models.” STATA Journal 15, no. 1: 3–20. 10.1177/1536867x1501500102.

[hec70092-bib-0004] Bjørner, T. B. , and S. Arnberg . 2012. “Terminal Costs, Improved Life Expectancy and Future Public Health Expenditure.” International Journal of Health Care Finance and Economics 12, no. 2: 129–143. 10.1007/s10754-012-9106-1. 22407513

[hec70092-bib-0005] Blinder, A. S. 1973. “Wage Discrimination: Reduced Form and Structural Estimates.” Journal of Human Resources 8, no. 4: 436–455. 10.2307/144855.

[hec70092-bib-0006] Breyer, F. , and N. Lorenz . 2021. “The ‘Red Herring’ After 20 Years: Ageing and Health Care Expenditures.” European Journal of Health Economics 22, no. 5: 661–667. 10.1007/s10198-020-01203-x. PMC8214577 32500244

[hec70092-bib-0007] Buchner, F. , and J. Wasem . 2006. “‘Steeping’ of Health Expenditure Profiles.” Geneva Papers on Risk and Insurance ‐ Issues and Practice 31, no. 4: 581–599. 10.1057/palgrave.gpp.2510100.

[hec70092-bib-0008] Carreras, M. , P. Ibern , and J. M. Inoriza . 2018. “Ageing and Healthcare Expenditures: Exploring the Role of Individual Health Status.” Health Economics 27, no. 5: 865–876. 10.1002/hec.3635. 29424031

[hec70092-bib-0009] Chandra, A. , and J. Skinner . 2012. “Technology Growth and Expenditure Growth in Health Care.” Journal of Economic Literature 50, no. 3: 645–680. 10.1257/jel.50.3.645.

[hec70092-bib-0010] Christensen, B. J. , M. Gørtz , and M. Kallestrup‐Lamb . 2016. “Medical Spending in Denmark.” Fiscal Studies 37, no. 3–4: 461–497. 10.1111/j.1475-5890.2016.12119.

[hec70092-bib-0011] Christensen, K. , G. Doblhammer , R. Rau , and J. W. Vaupel . 2009. “Ageing Populations: The Challenges Ahead.” Lancet 374, no. 9696: 1196–1208. 10.1016/s0140-6736(09)61460-4. 19801098 PMC2810516

[hec70092-bib-0012] Costa‐Font, J. , and C. Vilaplana‐Prieto . 2020. “‘More Than One Red Herring’? Heterogeneous Effects of Ageing on Health Care Utilisation.” Health Economics 29, no. S1: 8–29. 10.1002/hec.4035. 32677116

[hec70092-bib-0013] Cragg, J. G. 1971. “Some Statistical Models for Limited Dependent Variables With Application to the Demand for Durable Goods.” Econometrica: Journal of the Econometric Society 39, no. 5: 829–844. 10.2307/1909582.

[hec70092-bib-0014] Danish Health Data Authority . 2024. “Dødsårsagsregisteret – Rapport 2024.” Sundhedsdatastyrelsen. Datagrundlag: Dødsårsagsregisteret pr. 30, Technical report: juni 2024.

[hec70092-bib-0015] Danmarks Nationalbank . 2024. Yearly Exchange Rate. Data retrieved on 05/02/2024.

[hec70092-bib-0016] Deb, P. , and E. C. Norton . 2018. “Modeling Health Care Expenditures and Use.” Annual Review of Public Health 39, no. 1: 489–505. 10.1146/annurev-publhealth-040617-013517. 29328879

[hec70092-bib-0017] Dieleman, J. L. , M. Weil , M. Beauchamp , et al. 2025. “Drivers of Variation in Health Care Spending Across US Counties.” JAMA Health Forum 6, no. 2: e245220. 10.1001/jamahealthforum.2024.5220. 39951314 PMC11829242

[hec70092-bib-0018] Dorfman, R. 1938. “A Note on the Δ‐Method for Finding Variance Formulae.” Biometric Bulletin.

[hec70092-bib-0019] Engle, R. F. 1984. “Wald, Likelihood Ratio, and Lagrange Multiplier Tests in Econometrics.” Handbook of Econometrics 2: 775–826.

[hec70092-bib-0020] Federal Reserve (US) . 2025a. South Korean Won to U.S. Dollar Spot Exchange Rate. H.10 Foreign Exchange Rates Release. Retrieved from FRED. Federal Reserve Bank of St. Louis, March 13, 2025.

[hec70092-bib-0021] Federal Reserve (US) . 2025b. U.S. Dollars to Euro Spot Exchange Rate [dexuseu]. Retrieved from FRED, Federal Reserve Bank of St. Louis on March 13, 2025. https://fred.stlouisfed.org/series/DEXUSEU.

[hec70092-bib-0022] Felder, S. , and A. Werblow . 2008. “Does the Age Profile of Health Care Expenditure Really Steepen Over Time? New Evidence From Swiss Cantons.” Geneva Papers on Risk and Insurance ‐ Issues and Practice 33, no. 4: 710–727. 10.1057/gpp.2008.28.

[hec70092-bib-0023] Felder, S. , A. Werblow , and P. Zweifel . 2010. “Do Red Herrings Swim in Circles? Controlling for the Endogeneity of Time to Death.” Journal of Health Economics 29, no. 2: 205–212. 10.1016/j.jhealeco.2009.11.014. 20022392

[hec70092-bib-0024] French, E. B. , J. McCauley , M. Aragon , et al. 2017. “End‐of‐Life Medical Spending in Last Twelve Months of Life Is Lower Than Previously Reported.” Health Affairs 36, no. 7: 1211–1217. 10.1377/hlthaff.2017.0174. 28679807

[hec70092-bib-0025] Geue, C. , A. Briggs , J. Lewsey , and P. Lorgelly . 2014. “Population Ageing and Healthcare Expenditure Projections: New Evidence From a Time to Death Approach.” European Journal of Health Economics 15, no. 8: 885–896. 10.1007/s10198-013-0543-7. 24292437

[hec70092-bib-0026] Gourieroux, C. , A. Monfort , and A. Trognon . 1984. “Pseudo Maximum Likelihood Methods: Theory.” Econometrica 52, no. 3: 681–700. 10.2307/1913471.

[hec70092-bib-0027] Greene, W. H. 2017. Econometric Analysis. 7th ed. Pearson.

[hec70092-bib-0028] Gregersen, F. A. 2014. “The Impact of Ageing on Health Care Expenditures: A Study of Steepening.” European Journal of Health Economics 15, no. 9: 979–989. 10.1007/s10198-013-0541-9. PMC4228175 24271039

[hec70092-bib-0029] Heckman, J. J. 1976. “The Common Structure of Statistical Models of Truncation, Sample Selection and Limited Dependent Variables and a Simple Estimator for Such Models.” In Annals of Economic and Social Measurement, Vol. 5, 4: 475–492. NBER.

[hec70092-bib-0030] Howdon, D. , and N. Rice . 2018. “Health Care Expenditures, Age, Proximity to Death and Morbidity: Implications for an Ageing Population.” Journal of Health Economics 57: 60–74. 10.1016/j.jhealeco.2017.11.001. 29182935

[hec70092-bib-0031] Hyun, K.‐R. , S. Kang , and S. Lee . 2016. “Population Aging and Healthcare Expenditure in Korea.” Health Economics 25, no. 10: 1239–1251. 10.1002/hec.3209. 26085120

[hec70092-bib-0032] Insurance and Pension (FP) . 2023. Sundhedsforsikringerne finansierer hovedsageligt, hvor der er egenbetaling på sundhedsområdet. Report authored by Sophie Orebo Wenzel: Published on 29.11.2023.

[hec70092-bib-0033] Kallestrup‐Lamb, M. , A. O. Marin , S. Menon , and J. Søgaard . 2024. “Aging Populations and Expenditures on Health.” Journal of the Economics of Ageing 29: 100518. 10.1016/j.jeoa.2024.100518.

[hec70092-bib-0034] Karlsson, M. and F. Klohn . 2011. “Some Notes on How to Catch a Red Herring: Ageing, Time‐To‐Death & Care Costs for Older People in Sweden.” Technical report, Darmstadt Discussion Papers in Economics.

[hec70092-bib-0035] Karlsson, M. , and F. Klohn . 2014. “Testing the Red Herring Hypothesis on an Aggregated Level: Ageing, Time‐To‐Death and Care Costs for Older People in sweden.” European Journal of Health Economics 15, no. 5: 533–551. 10.1007/s10198-013-0493-0. 23868467

[hec70092-bib-0036] Kass, R. E. , and A. E. Raftery . 1995. “Bayes Factors.” Journal of the American Statistical Association 90, no. 430: 773–795. 10.1080/01621459.1995.10476572.

[hec70092-bib-0037] Kitagawa, E. M. 1955. “Components of a Difference Between Two Rates.” Journal of the American Statistical Association 50, no. 272: 1168–1194. 10.1080/01621459.1955.10501299.

[hec70092-bib-0038] Kollerup, A. , J. Kjellberg , and R. Ibsen . 2022. “Ageing and Health Care Expenditures: The Importance of Age Per Se, Steepening of the Individual‐Level Expenditure Curve, and the Role of Morbidity.” European Journal of Health Economics 23, no. 7: 1–29. 10.1007/s10198-021-01413-x. 35037122

[hec70092-bib-0039] Kolodziejczyk, C. 2020. “The Effect of Time to Death on Health Care Expenditures: Taking Into Account the Endogeneity and Right Censoring of Time to Death.” European Journal of Health Economics 21, no. 6: 945–962. 10.1007/s10198-020-01187-8. 32328875

[hec70092-bib-0040] Laudicella, M. , P. Li Donni , K. R. Olsen , and D. Gyrd‐Hansen . 2022. “Age, Morbidity, or Something Else? A Residual Approach Using Microdata to Measure the Impact of Technological Progress on Health Care Expenditure.” Health Economics 31, no. 6: 1184–1201. 10.1002/hec.4500. 35362244 PMC9314678

[hec70092-bib-0041] Liang, K.‐Y. , and S. L. Zeger . 1986. “Longitudinal Data Analysis Using Generalized Linear Models.” Biometrika 73, no. 1: 13–22. 10.1093/biomet/73.1.13.

[hec70092-bib-0042] Manning, W. G. , and J. Mullahy . 2001. “Estimating Log Models: To Transform or Not to Transform?” Journal of Health Economics 20, no. 4: 461–494. 10.1016/s0167-6296(01)00086-8. 11469231

[hec70092-bib-0043] Maynou, L. , A. Street , A. García , et al. 2023. “Living Longer in Declining Health: Factors Driving Healthcare Costs Among Older People.” Social Science & Medicine 327: 115955. 10.1016/j.socscimed.2023.115955. 37196394

[hec70092-bib-0044] Moore, P. V. , K. Bennett , and C. Normand . 2017. “Counting the Time Lived, the Time Left or Illness? Age, Proximity to Death, Morbidity and Prescribing Expenditures.” Social Science & Medicine 184: 1–14. 10.1016/j.socscimed.2017.04.038. 28482276

[hec70092-bib-0045] Norton, E. C. , and B. E. Dowd . 2018. “Log Odds and the Interpretation of Logit Models.” Health Services Research 53, no. 2: 859–878. 10.1111/1475-6773.12712. 28560732 PMC5867187

[hec70092-bib-0046] Oaxaca, R. 1973. “Male‐female Wage Differentials in Urban Labor Markets.” International Economic Review 14, no. 3: 693–709. 10.2307/2525981.

[hec70092-bib-0047] Polder, J. J. , J. J. Barendregt , and H. van Oers . 2006. “Health Care Costs in the Last Year of Life—The Dutch Experience.” Social Science & Medicine 63, no. 7: 1720–1731. 10.1016/j.socscimed.2006.04.018. 16781037

[hec70092-bib-0048] Ricketts, T. C. 2011. “The Health Care Workforce: Will it Be Ready as the Boomers Age? A Review of How We Can Know (Or Not Know) the Answer.” Annual Review of Public Health 32, no. 1: 417–430. 10.1146/annurev-publhealth-031210-101227. 21219159

[hec70092-bib-0049] Rosella, L. , K. Kornas , A. Huang , C. Bornbaum , D. Henry , and W. P. Wodchis . 2018. “Accumulation of Chronic Conditions at the Time of Death Increased in Ontario From 1994 to 2013.” Health Affairs 37, no. 3: 464–472. 10.1377/hlthaff.2017.1150. 29505380

[hec70092-bib-0050] Salas, C. , and J. P. Raftery . 2001. “Econometric Issues in Testing the Age Neutrality of Health Care Expenditure.” Health Economics 10, no. 7: 669–671. 10.1002/hec.638. 11747048

[hec70092-bib-0051] Seshamani, M. , and A. Gray . 2004a. “Ageing and Health‐Care Expenditure: The Red Herring Argument Revisited.” Health Economics 13, no. 4: 303–314. 10.1002/hec.826. 15067669

[hec70092-bib-0052] Seshamani, M. , and A. M. Gray . 2004b. “A Longitudinal Study of the Effects of Age and Time to Death on Hospital Costs.” Journal of Health Economics 23, no. 2: 217–235. 10.1016/j.jhealeco.2003.08.004. 15019753

[hec70092-bib-0053] Statens Serum Institut . 2014. Takstsystem Vejledning.

[hec70092-bib-0054] Statistics Denmark . 2024. “Statistikdokumentation for Befolkningen 2024.” Technical report: Danmarks Statistik, Copenhagen, Danmark.

[hec70092-bib-0055] Statistics Denmark . 2023. “Danish Register Data for Research – Access via Statistics denmark.” Accessed through Statistics Denmark’s research services. https://www.dst.dk/en/TilSalg/Forskningsservice.

[hec70092-bib-0056] Statistics Denmark . 2024. Consumer Price Index. Data retrieved on 05/02/2024.

[hec70092-bib-0057] Statistics Denmark . 2025. “SHA1: Udgifter Til Sundhed Efter Funktion, Aktør, Finansieringskilde Og Prisenhed.” Accessed 06 18, 2025. Enhed: Mio. kr. (løbende priser). https://www.statistikbanken.dk/SHA1.

[hec70092-bib-0058] Tanuseputro, P. , W. P. Wodchis , R. Fowler , et al. 2015. “The Health Care Cost of Dying: A Population‐Based Retrospective Cohort Study of the Last Year of Life in Ontario, Canada.” PLoS One 10, no. 3: e0121759. 10.1371/journal.pone.0121759. 25811195 PMC4374686

[hec70092-bib-0059] van Baal, P. H. , and A. Wong . 2012. “Time to Death and the Forecasting of Macro‐Level Health Care Expenditures: Some Further Considerations.” Journal of Health Economics 31, no. 6: 876–887. 10.1016/j.jhealeco.2012.08.003. 23000700

[hec70092-bib-0060] v o n Wyl, V. 2019. “Proximity to Death and Health Care Expenditure Increase Revisited: A 15‐Year Panel Analysis of Elderly Persons.” Health Economics Review 9, no. 1: 1–16. 10.1186/s13561-019-0224-z. 30859485 PMC6734245

[hec70092-bib-0061] Wald, A. 1943. “Tests of Statistical Hypotheses Concerning Several Parameters When the Number of Observations Is Large.” Transactions of the American Mathematical Society 54, no. 3: 426–482. 10.1090/s0002-9947-1943-0012401-3.

[hec70092-bib-0062] Welch, B. L. 1947. “The Generalization of Student’s’ Problem when Several Different Population Variances Are Involved.” Biometrika 34, no. 1/2: 28–35. 10.2307/2332510. 20287819

[hec70092-bib-0063] Werblow, A. , S. Felder , and P. Zweifel . 2007. “Population Ageing and Health Care Expenditure: A School of ‘red Herrings’.” Health Economics 16, no. 10: 1109–1126. 10.1002/hec.1213. 17311357

[hec70092-bib-0064] White, H. 1982. “Maximum Likelihood Estimation of Misspecified Models.” Econometrica 50: 1–25. 10.2307/1912526.

[hec70092-bib-0065] WHO . 2024. “Current Health Expenditure (% of GDP) ‐ United States, Euro Area.” Data retrieved from the WHO Global Health Expenditure database on April 15, 2024. apps.who.int/nha/database.

[hec70092-bib-0068] Zweifel, P. , S. Felder , and M. Meiers . 1999. “Ageing of Population and Health Care Expenditure: A Red Herring?” Health Economics 8, no. 6: 485–496. 10.1002/(sici)1099-1050(199909)8:6<485::aid-hec461>3.0.co;2-4. 10544314

