# Supplementary material for: Competing Demographic Drivers of Hospital Expenditures: Coexistence of the Red Herring and the Steepening Effects
Source: Health Econ. 2026 Mar 7;35(6):929–46. doi: 10.1002/hec.70092 (PMC13126104; doi:10.1002/hec.70092)
Supplement: Supplementary file 1 — Supporting Information S1 [file HEC-35-929-s001.pdf]

Online Appendix For: Competing Demographic Drivers of Hospital Expenditures: Coexistence of the Red Herring and the Steepening Effects*

Malene Kallestrup-Lamb
Aarhus University and PeRCent
Alexander O.K. Marin†
University of Southern Denmark, CPop
Jes Søgaard
University of Southern Denmark, CPop

February 23, 2026

*Authors listed alphabetically.

†Corresponding Author. Campusvej 55, 5230 Odense M, Denmark. Email: aokm@sam.sdu.dk

B Online Appendix: Methodology

B.1 Steepening effects Despite Null Age-Time Interactions

In this section, we show that Steepening effects may exist event though interaction effects, δ_{AT} and γ_{AT}, are zero. Consider the full model $\psi = F \cdot g$, under the joint model specification where we suppress the dependence on linear arguments, $X'\delta$ and $X'\gamma$, in the Joint Model in equation (5). The second order derivative of ψ with respect to age, A, and calendar time, T, considered the Steepening effect (Gregersen, 2014), is

$$\begin{aligned}
\partial^2\psi / \left(\partial A \partial T\right) = & F'' \cdot \left[\delta_T + A\delta_{AT}\right] \cdot \left[\delta_A + T\delta_{AT}\right] \cdot g \\
& + F' \cdot \delta_{AT} \cdot g \\
& + F' \cdot \left[\delta_A + T\delta_{AT}\right] \cdot g' \cdot \left[\gamma_T + A\gamma_{AT}\right] \\
& + F' \cdot \left[\delta_T + A\delta_{AT}\right] \cdot g' \cdot \left[\gamma_A + T\gamma_{AT}\right] \\
& + F \cdot g'' \cdot \left[\gamma_T + A\gamma_{AT}\right] \cdot \left[\gamma_A + T\gamma_{AT}\right] \\
& + F \cdot g' \cdot \gamma_{AT},
\end{aligned} \tag{20}$$

for $'$ and $''$ indicating a first and second order derivative, respectively, of a function.

In the case where the interaction terms are zero, $\delta_{AT} = \gamma_{AT} = 0$, the expression simplifies to

$$\begin{aligned}
\partial^2\psi / \left(\partial A \partial T\right) = & F'' \cdot \delta_T \cdot \delta_A \cdot g \\
& + F' \cdot \delta_A \cdot g' \cdot \gamma_T \\
& + F' \cdot \delta_T \cdot g' \cdot \gamma_A \\
& + F \cdot g'' \cdot \gamma_T \cdot \gamma_A .
\end{aligned} \tag{21}$$

Although the age-time interaction parameters are zero, Steepening effect could persist, i.e., $\partial^2\psi / \left(\partial A \partial T\right) > 0$, depending on the relative size of the remaining terms in equation (21). Similar arguments can be made for the first part function, F, and the second part function, g, where the second order derivatives are

$$\partial^2 F / \left(\partial A \partial T\right) = F'' \cdot \left[\delta_T + A\delta_{AT}\right] \cdot \left[\delta_A + T\delta_{AT}\right] + F' \cdot \delta_{AT} \tag{22}$$

$$\partial^2 g / \left(\partial A \partial T\right) = g'' \cdot \left[\gamma_T + A\gamma_{AT}\right] \cdot \left[\gamma_A + T\gamma_{AT}\right] + g' \cdot \gamma_{AT} . \tag{23}$$

Our approach for addressing these cases of steepening is to test the average marginal effects of the estimated models with the Delta Method and apply Wald tests of joint significance of the marginal effects as discussed in Section 2.2.

B.2 Robustness: Statistical Tests

Several statistical tests exist to test zero-restrictions on parameters of interest (Engle, 1984). So far, we have utilized the Wald test, to test the three hypotheses of Section 2.2. While a test on the parameter estimates themselves is of interest, much of our research has focused on the marginal association on more interpretable outcomes, i.e., the probability of using hospital care, and the numerical dollar-amount of hospital expenditures, as calculated with the Delta Method. To

assess the robustness of our findings towards this different outcome, we redefine the hypotheses to reflect the marginal association

$$
\begin{aligned}
H_0^{me,1}: &\quad \mathbb{E}\left[\frac{\partial f(H_i,\theta)}{\partial TTD} \quad \frac{\partial f(H_i,\theta)}{\partial T} \quad \frac{\partial f(H_i,\theta)}{\partial A\cdot T}\right] = \begin{bmatrix}0 & 0 & 0\end{bmatrix}, \\
H_0^{me,2}: &\quad \mathbb{E}\left[\frac{\partial f(H_i,\theta)}{\partial A} \quad \frac{\partial f(H_i,\theta)}{\partial T} \quad \frac{\partial f(H_i,\theta)}{\partial A\cdot T}\right] = \begin{bmatrix}0 & 0 & 0\end{bmatrix}, \\
H_0^{me,3}: &\quad \mathbb{E}\left[\frac{\partial f(H_i,\theta)}{\partial TTD}\right] = \begin{bmatrix}0\end{bmatrix},
\end{aligned}
\tag{24}
$$

where the function, f, which can be (i) the joint model, ψ, (ii) the first part, F, or (iii) the second part, g, in equation (1). As each of the marginal associations from the Delta Method are Gaussian under maximum likelihood estimation, a Wald test can also be used to test the hypotheses in equation (7) (Wooldridge, 2010, Chapter 3).

Traditional hypothesis testing, e.g., the Wald test, has a tendency to reject most null-hypotheses in large samples due to small p-value, i.e., the probability of observing data given a hypothesis, $Pr\left(D \mid H_0\right)$. To add robustness on this issue, we employ a second testing framework to the hypotheses in equation (6) which does not share this feature. This approach focuses on the probability of the null hypothesis, H_0, against the alternative, H_A, given the data, D, i.e., $Pr\left(H_0 \mid D\right)$. These p-values may align more closely with a researchers testing objective by evaluation evidence in favor of a null hypothesis. As advocated by Kass and Raftery (1995), such *Bayesian p-values* can be derived from Bayes formula and require Bayes factors and a prior odds ratio for calculation

$$
Pr\left(H_0 \mid D\right) = \frac{\frac{Pr(D|H_0)}{Pr(D|H_A)} \times \frac{Pr(H_0)}{Pr(H_A)}}{1 + \frac{Pr(D|H_0)}{Pr(D|H_A)} \times \frac{Pr(H_0)}{Pr(H_A)}} = \frac{\text{Bayes factor} \times \text{prior odds}}{1 + \text{Bayes factor} \times \text{prior odds}}. \tag{25}
$$

Bayes factors can be interpreted as likelihood ratios, i.e., how good the model fits under the null compared to the alternative. The prior odds signifies the researchers prior beliefs regarding the null hypothesis compared to its alternative, prior odds $= Pr\left(H_0\right) / Pr\left(H_A\right)$. A critique of the Bayesian p-values are their dependence on subjective beliefs about the prior odds. Specifically, the prior odds could be hacked ex-post if a certain p-value is desired. A common practice is, however, to assess the robustness of the Bayesian p-values resulting from various prior odds levels. We derive the derivation of equation (25) and the estimation of the Bayes factor in Appendix B.3.

We apply these two tests to the full sample estimates from Section 4, and report the results in Table A1. The p-values of the Wald-tests on the marginal effects are close to zero and reject all null-hypotheses at conventional significance-levels .[24] The Bayesian p-value tests provides a similar conclusion.[25] with p-values of zero offering no support for the null hypotheses.

[24] We note that H_0^3 has a p-value of 0.005 which is larger than the remaining p-values. An investigation reveals that relatively large marginal effects of time-to-death on the Poisson part of the model is the source of the low test-statistic.

[25] For the Bayesian tests we set a prior belief in the null of 20%. Due to the size of the Bayes factors, the Bayesian p-values remain zero even if we raise our beliefs in the null as much as up to 99%.

Table A1: Hypothesis test results

	Wald Test | Marginal Effect			Bayesian Test | Parameter		
	#Res.	Test Statistic	P-value	#Res.	log Bayes Factor	P-value
H_0^1	3	164,426	0.000	6	-84,883,437	0.000
H_0^2	3	8,081	0.000	6	-2,349,166	0.000
H_0^3	1	8	0.005	2	-84,613,510	0.000

Hypothesis tests and p-values. #Res. refers to the number of parameter restrictions. In the Bayesian test we set a 20% prior belief in the null.

B.3 Testing with Bayes Factor

This section discusses testing of our hypothesis in Section 2.2, but rather than using the classical p-values signifying the probability of observing data, D, given a null hypothesis, H_0, we consider the reverse, i.e., the probability of the null hypothesis given data. To this end, we take point of departure in Kass and Raftery (1995) concerning Bayes Factors.

Considering a null hypothesis, H_0, and its alternative, H_A, we obtain from Bayes's theorem

$$\underbrace{\frac{Pr(H_0 \mid D)}{Pr(H_A \mid D)}}_{\text{posterior odds}} = \underbrace{\frac{Pr(D \mid H_0)}{Pr(D \mid H_A)}}_{\text{Bayes factor}} \underbrace{\frac{Pr(H_0)}{Pr(H_A)}}_{\text{prior odds}} \tag{26}$$

where the Bayes factor, informally, has the interpretation of the likelihood ratio of the two hypotheses and the prior odds ratio signifies the prior relative belief towards the null and its alternative of the analyst. Note the restrictions that $1 = Pr\left(H_0 \mid D\right) + Pr\left(H_A \mid D\right)$ and $1 = Pr\left(H_0\right) + Pr\left(H_A\right)$. Using these relationships and reworking equation (26) we obtain an expression for the *Bayesian p-values*

$$Pr\left(H_0 \mid D\right) = \frac{\text{Bayes factor} \times \text{prior odds}}{1 + \text{Bayes factor} \times \text{prior odds}}. \tag{27}$$

the Bayesian p-values increase with the Bayes factor, and a higher likelihood of data stemming from a model under the null hypothesis increases evidence in favor of the null hypothesis. Conversely, a low Bayes factor implies poor relative evidence in favor of the null, i.e., the alternative fits data better. A common practice is to assess robustness of Bayesian p-values to various prior odds specifications, as the prior odds could otherwise be chosen ex-post to achieve desired results (Kass and Raftery, 1995). Our estimates remain robust to prior beliefs ranging from $Pr(H_0) = 0.01$ to $Pr(H_0) = 0.99$, demonstrating that our conclusions are insensitive to the choice of prior odds.

In large samples, Bayes factor is easily approximated as the exponential of the Schwarz criterion, S, (Kass and Raftery, 1995). Let $\hat{\ell}_{H_0}$ and $\hat{\ell}_{H_A}$ be the maximized log-likelihood functions under the null and its alternative, respectively, and $|.|$ be the length of its argument, then

$$S = \hat{\ell}_{H_0} - \hat{\ell}_{H_A} - \frac{1}{2}\left(|\theta_0| - |\theta_A|\right)\log(N), \tag{28}$$

which we use to calculate the Bayes p-values.

C Online Appendix: Results

C.1 Robustness to Expanded Parametrization

A potential concern with our testing of the coexistence of Red Herring and Steepening effects is that misspecification of the functional form may lead to biased inference. Our Joint Model, specified in equation (5), adopts a parsimonious structure centered on key regressors—age, time-to-death, and an age-time interaction. To assess the robustness of our findings under more flexible specifications, we re-estimate the models in Sections 4, 4.1, and 4.2 using an expanded parameterization. Following established practice in the literature, we augment the Joint Model with several regressors. These include: age squared (Carreras et al., 2018; Howdon and Rice, 2018); a binary indicator being one in the month of death, D, and its interactions with age (Carreras et al., 2018; Hyun et al., 2016) and calendar time (Gregersen, 2014; Kollerup et al., 2022); age interacted with time-to-death (Geue et al., 2014; Karlsson and Klohn, 2014); and time-to-death squared (Wong et al., 2011). This results in the following Expanded Joint Model

$$\text{Expanded Joint Model:} \quad \begin{aligned} X_i\delta^R &= \delta^R_{TTD}TTD_i + \delta^R_{TTD^2}TTD_i^2 + \delta^R_{TTD\cdot A}TTD_iA_i + \delta^R_A A_i + \delta^R_{A^2}A_i^2 \\ &\quad + \delta^R_T T_i + \delta^R_{A\cdot T}A_iT_i + \delta^R_{D\cdot T}D_iT_i + \delta^R_D D_i + \delta^R_{A\cdot D}A_iD_i + \delta^R\check{X}_i^R \\ X_i\gamma^R &= \gamma^R_{TTD}TTD_i + \gamma^R_{TTD^2}TTD_i^2 + \gamma^R_{TTD\cdot A}TTD_iA_i + \gamma^R_A A_i + \gamma^R_{A^2}A_i^2 \\ &\quad + \gamma^R_T T_i + \gamma^R_{A\cdot T}A_iT_i + \gamma^R_{D\cdot T}D_iT_i + \gamma^R_D D_i + \gamma^R_{A\cdot D}A_iD_i + \gamma^R\check{X}_i^R\,. \end{aligned} \tag{29}$$

Specifically, we augment the model with: Age squared (Carreras et al., 2018; Howdon and Rice, 2018); an indicator for the final time-period (month) of death, D, and its interaction with age (Carreras et al., 2018; Hyun et al., 2016); age interacted with time-to-death (Geue et al., 2014; Karlsson and Klohn, 2014); calendar time interacted with a death-indicator (Gregersen, 2014; Kollerup et al., 2022); and time-to-death squared (Wong et al., 2011). Equation (29) presents the expanded parametrization of the Probit and Poisson models

This expanded parametrization modifies the Jacobian and Hessian matrices of the maximum likelihood estimates, which in turn affect the calculation of cluster-robust standard errors used in hypothesis testing. While we incorporate these changes into our estimation procedure, we omit the explicit expressions for brevity.

The null hypotheses for the Wald tests under this expanded model are redefined as follows:

$$\begin{aligned} H_0^{1.R}:&\quad \delta_{TTD} = \gamma_{TTD} = \delta_{TTD^2} = \gamma_{TTD^2} = \delta_{TTD\cdot A} = \gamma_{TTD\cdot A} = \delta_T = \gamma_T \\ &\quad = \delta_{A\cdot T} = \gamma_{A\cdot T} = \delta_{D\cdot T} = \gamma_{D\cdot T} = \delta_D = \gamma_D = \delta_{A\cdot D} = \gamma_{A\cdot D} = 0\,, \\ H_0^{2.R}:&\quad \delta_A = \gamma_A = \delta_{A^2} = \gamma_{A^2} = \delta_T = \gamma_T = \delta_{A\cdot T} = \gamma_{A\cdot T} = 0\,, \\ H_0^{3.R}:&\quad \delta_{TTD} = \gamma_{TTD} = \delta_{TTD^2} = \gamma_{TTD^2} = \delta_{TTD\cdot A} = \gamma_{TTD\cdot A} \\ &\quad = \delta_{D\cdot T} = \gamma_{D\cdot T} = \delta_D = \gamma_D = \delta_{A\cdot D} = \gamma_{A\cdot D} = 0\,. \end{aligned} \tag{30}$$

We treat the month-of-death indicator D and its interactions as capturing Red Herring effects, and therefore constrain these terms to zero in $H_0^{1.R}$ and $H_0^{3.R}$.

Using the hypotheses in equation (30), we perform Wald tests and compute Bayesian p-values

for: (i) the full population (Table A2); (ii) the age- and sex-disaggregated analyses (Table A4); and (iii) the time-to-death and sex analyses (Table A6).

We also reassess the marginal associations from equation (7) using the Delta Method for the full two-part model E_{ψ}, the extensive margin E_F, and the intensive margin E_g. Table A3 reports results for the full population, with Tables A5 and A7 reporting results for the age/sex and time-to-death/sex analyses, respectively.

In conclusion, the results strongly reject the null hypotheses in all cases. This confirms the simultaneous presence of Red Herring and Steepening effects, even under the more flexible functional form. Therefore, our main findings are robust to alternative model specifications.

Table A2: Hypothesis Tests of Hypothesis in Equation (30) under Expanded Joint Model

**Hypothesis**		**Wald Test**		**Bayesian Test**	
	#Res.	Test Statistic	P-value	log Bayes Factor	P-value
H_0^1	16	1,118,767	0.0000000	-328,067,347	0.0000000
H_0^2	8	48,749	0.0000000	-7,831,510	0.0000000
H_0^3	12	814,038	0.0000000	-326,240,933	0.0000000

Note: Hypothesis tests statistics and p-values. #Res. refers to the number of parameter restrictions. In the Bayesian test we set a 50% prior belief in the null.

Table A3: Hypothesis Tests of Hypothesis in Equation (7) under Expanded Joint Model

**Hypothesis**			**Wald Test, E$_F$**		**Wald Test, E$_g$**		**Wald Test, E$_{\psi}$**	
	#Res.	DoF	Test Stat.	P-value	Test Stat.	P-value	Test Stat.	P-value
H_0^1	3	9,999,952	346,768.317	0.0000000	3,039,579.914	0.0000000	118,084.354	0.0000000
H_0^2	3	9,999,952	92,528.466	0.0000000	29,031.123	0.0000000	27,188.324	0.0000000
H_0^3	1	9,999,952	21,394.488	0.0000000	2,959,613.833	0.0000000	30,890.132	0.0000000

Note: Wald test of marginal association by age and sex with null hypotheses in equation (7) based on the time-to-death- and sex-specific estimates in the Expanded Joint Model. #Res. refers to the number of parameter restrictions and DoF is the degrees of freedom.

Table A4: Hypothesis Tests of Age and Sex Specific Estimates under Expanded Joint Model

	Hypothesis		Wald Test		Bayes P-value	
Ages		#Res.	Test Stat.	P-value	Test Stat.	P-value
			**Female**			
[30;40)	H_0^1	16	625,110	0.0000000	-4,432,753	0.0000000
	H_0^2	8	267,949	0.0000000	-109,286	0.0000000
	H_0^3	12	208,661	0.0000000	-4,408,259	0.0000000
[40;50)	H_0^1	16	910,055	0.0000000	-13,806,931	0.0000000
	H_0^2	8	265,047	0.0000000	-22,810	0.0000000
	H_0^3	12	577,317	0.0000000	-13,767,837	0.0000000
[50;60)	H_0^1	16	3,288,241	0.0000000	-30,680,584	0.0000000
	H_0^2	8	715,628	0.0000000	-86,115	0.0000000
	H_0^3	12	1,494,231	0.0000000	-30,564,493	0.0000000
[60;70)	H_0^1	16	2,443,508	0.0000000	-47,107,619	0.0000000
	H_0^2	8	165,068	0.0000000	-78,510	0.0000000
	H_0^3	12	2,102,277	0.0000000	-46,859,294	0.0000000
[70;80)	H_0^1	16	14,111,603	0.0000000	-39,080,544	0.0000000
	H_0^2	8	411,176	0.0000000	-48,459	0.0000000
	H_0^3	12	7,341,043	0.0000000	-38,848,315	0.0000000
[80;90)	H_0^1	16	13,295,886	0.0000000	-21,849,346	0.0000000
	H_0^2	8	589,433	0.0000000	-167,940	0.0000000
	H_0^3	12	12,244,362	0.0000000	-821,545,574	0.0000000
90+	H_0^1	16	15,558,576	0.0000000	-4,137,558	0.0000000
	H_0^2	8	629,804	0.0000000	-79,414	0.0000000
	H_0^3	12	11,091,372	0.0000000	-4,017,525	0.0000000
			**Male**			
[30;40)	H_0^1	16	451,512	0.0000000	-3,126,854	0.0000000
	H_0^2	8	101,273	0.0000000	-130,180	0.0000000
	H_0^3	12	180,004	0.0000000	-3,015,781	0.0000000
[40;50)	H_0^1	16	1,213,452	0.0000000	-12,138,141	0.0000000
	H_0^2	8	246,400	0.0000000	-133,759	0.0000000
	H_0^3	12	592,620	0.0000000	-12,032,795	0.0000000
[50;60)	H_0^1	16	5,654,526	0.0000000	-33,033,256	0.0000000
	H_0^2	8	912,401	0.0000000	-70,631	0.0000000
	H_0^3	12	3,493,114	0.0000000	-32,890,146	0.0000000
[60;70)	H_0^1	16	6,400,946	0.0000000	-56,041,409	0.0000000
	H_0^2	8	802,795	0.0000000	-42,454	0.0000000
	H_0^3	12	3,895,920	0.0000000	-55,899,972	0.0000000
[70;80)	H_0^1	16	16,587,405	0.0000000	-50,661,974	0.0000000
	H_0^2	8	160,869	0.0000000	-113,685	0.0000000
	H_0^3	12	9,874,884	0.0000000	-50,227,724	0.0000000
[80;90)	H_0^1	16	17,166,450	0.0000000	-23,349,059	0.0000000
	H_0^2	8	123,795	0.0000000	-42,295	0.0000000
	H_0^3	12	11,668,831	0.0000000	-23,278,599	0.0000000
90+	H_0^1	16	17,657,174	0.0000000	-2,832,435	0.0000000
	H_0^2	8	1,420,596	0.0000000	-40,389	0.0000000
	H_0^3	12	14,435,009	0.0000000	-2,773,288	0.0000000

Note: Wald test from equation (30) and Bayesian p-values from Section B.2 of parameterestimates, $\hat{\theta}$ by age and sex from the Expanded Joint Model. #Res. refers to the number of parameter restrictions.

Table A5: Hypothesis Tests of Hypothesis in equation (7) by Age and Sex under Expanded Joint Model

	Hypothesis			Wald Test, E_F		Wald Test, E_g		Wald Test, E_ψ	
Ages		#Res.	DoF	Test Stat.	P-value	Test Stat.	P-value	Test Stat.	P-value
**Female**									
[30;40)	H_0^1	3	1,036,228	2,118.141	0.0000000	41,288.498	0.0000000	143,823.488	0.0000000
	H_0^2	3	1,036,228	223.948	0.0000000	1,473.026	0.0000000	54,711.307	0.0000000
	H_0^3	1	1,036,228	1,582.922	0.0000000	14,197.605	0.0000000	14,704.945	0.0000000
[40;50)	H_0^1	3	1,107,303	6,399.278	0.0000000	53,320.083	0.0000000	42,751.564	0.0000000
	H_0^2	3	1,107,303	465.923	0.0000000	291.910	0.0000000	4,962.143	0.0000000
	H_0^3	1	1,107,303	1,779.493	0.0000000	42,919.724	0.0000000	29,976.391	0.0000000
[50;60)	H_0^1	3	1,041,255	2,408.184	0.0000000	50,888.055	0.0000000	60,977.516	0.0000000
	H_0^2	3	1,041,255	351.239	0.0000000	252.543	0.0000000	17,721.526	0.0000000
	H_0^3	1	1,041,255	2,185.619	0.0000000	44,447.489	0.0000000	37,109.120	0.0000000
[60;70)	H_0^1	3	912,012	3,924.966	0.0000000	170,682.218	0.0000000	37,085.658	0.0000000
	H_0^2	3	912,012	529.292	0.0000000	965.505	0.0000000	9,799.301	0.0000000
	H_0^3	1	912,012	3,314.338	0.0000000	167,819.359	0.0000000	18,652.898	0.0000000
[70;80)	H_0^1	3	610,708	5,276.992	0.0000000	3,614,664.421	0.0000000	97,711.373	0.0000000
	H_0^2	3	610,708	1,309.773	0.0000000	640.359	0.0000000	1,524.370	0.0000000
	H_0^3	1	610,708	4,971.440	0.0000000	3,102,102.022	0.0000000	34,390.849	0.0000000
[80;90)	H_0^1	3	337,229	3,503.570	0.0000000	1,771,828.371	0.0000000	207,744.482	0.0000000
	H_0^2	3	337,229	1,289.984	0.0000000	1,333.324	0.0000000	10,034.419	0.0000000
	H_0^3	1	337,229	3,364.055	0.0000000	1,302,267.971	0.0000000	181,120.534	0.0000000
90+	H_0^1	3	80,360	11,537.804	0.0000000	461,392.766	0.0000000	114,792.760	0.0000000
	H_0^2	3	80,360	18,759.534	0.0000000	14,507.675	0.0000000	4,221.451	0.0000000
	H_0^3	1	80,360	6,754.282	0.0000000	453,783.059	0.0000000	114,317.975	0.0000000
**Male**									
[30;40)	H_0^1	3	1,056,500	1,014.451	0.0000000	51,861.849	0.0000000	31,201.542	0.0000000
	H_0^2	3	1,056,500	441.090	0.0000000	1,912.917	0.0000000	9,598.894	0.0000000
	H_0^3	1	1,056,500	776.931	0.0000000	35,483.666	0.0000000	16,525.549	0.0000000
[40;50)	H_0^1	3	1,133,032	4,488.343	0.0000000	98,795.724	0.0000000	17,911.900	0.0000000
	H_0^2	3	1,133,032	344.971	0.0000000	746.480	0.0000000	9,918.820	0.0000000
	H_0^3	1	1,133,032	400.352	0.0000000	39,391.042	0.0000000	5,800.959	0.0000000
[50;60)	H_0^1	3	1,049,851	3,838.470	0.0000000	479,845.930	0.0000000	38,440.381	0.0000000
	H_0^2	3	1,049,851	359.506	0.0000000	165.977	0.0000000	10,657.866	0.0000000
	H_0^3	1	1,049,851	1,857.579	0.0000000	347,911.177	0.0000000	18,816.454	0.0000000
[60;70)	H_0^1	3	883,124	4,600.196	0.0000000	4,899,210.744	0.0000000	23,364.738	0.0000000
	H_0^2	3	883,124	961.886	0.0000000	364.596	0.0000000	9,452.976	0.0000000
	H_0^3	1	883,124	4,561.055	0.0000000	1,974,072.205	0.0000000	20,091.659	0.0000000
[70;80)	H_0^1	3	519,976	20,743.832	0.0000000	1,111,247.113	0.0000000	202,675.625	0.0000000
	H_0^2	3	519,976	1,416.525	0.0000000	2,377.588	0.0000000	22,721.047	0.0000000
	H_0^3	1	519,976	6,995.211	0.0000000	845,818.193	0.0000000	168,789.781	0.0000000
[80;90)	H_0^1	3	204,547	34,629.140	0.0000000	1,134,279.542	0.0000000	145,606.976	0.0000000
	H_0^2	3	204,547	3,459.459	0.0000000	4,326.261	0.0000000	2,487.632	0.0000000
	H_0^3	1	204,547	20,518.342	0.0000000	987,192.789	0.0000000	145,276.026	0.0000000
90+	H_0^1	3	27,231	76,094.181	0.0000000	3,981,435.865	0.0000000	442,225.455	0.0000000
	H_0^2	3	27,231	29,048.400	0.0000000	15,787.851	0.0000000	53,467.864	0.0000000
	H_0^3	1	27,231	57,916.018	0.0000000	3,165,851.992	0.0000000	365,881.426	0.0000000

Note: Wald test of marginal association by age and sex with null hypotheses in equation (7) based on the age- and sex-specific estimates in the Expanded Joint Model. #Res. refers to the number of parameter restrictions and DoF is the degrees of freedom.

Table A6: Hypothesis Tests of Age and Time-to-death Specific Estimates under Expanded Joint Model

	Hypothesis		Wald Test		Bayes P-value	
Age Group		#Res.	Test Statistic	P-value	Test Statistic	P-value
			**Female**			
TTD ∈ [0;1.5)	H_0^1	16	66,199,153	0.0000000	-60,404,639	0.0000000
	H_0^2	8	2,215,737	0.0000000	-1,781,561	0.0000000
	H_0^3	12	60,404,639	0.0000000	-1,781,561	0.0000000
TTD ∈ [1.5;4)	H_0^1	12	1,781,561	0.0000000	-901,071	0.0000000
	H_0^2	8	896,212	0.0000000	-505,229	0.0000000
	H_0^3	6	2,329,817	0.0000000	-708,541	0.0000000
TTD > 4	H_0^1	4	22,766	0.0000000	-469,675	0.0000000
	H_0^2	8	99,150	0.0000000	-10,159,673	0.0000000
	H_0^3	NA	NA	NA	NA	NA
			**Male**			
TTD ∈ [0;1.5)	H_0^1	16	111,276,343	0.0000000	-35,286,791	0.0000000
	H_0^2	8	4,168,894	0.0000000	-2,185,768	0.0000000
	H_0^3	12	95,300,994	0.0000000	-34,559,923	0.0000000
TTD ∈ [1.5;4)	H_0^1	12	5,913,072	0.0000000	-1,219,566	0.0000000
	H_0^2	8	2,180,218	0.0000000	-758,601	0.0000000
	H_0^3	6	2,976,652	0.0000000	-1,077,573	0.0000000
TTD > 4	H_0^1	4	11,139	0.0000000	-487,458	0.0000000
	H_0^2	8	189,505	0.0000000	-8,384,418	0.0000000
	H_0^3	NA	NA	NA	NA	NA

Note: Wald test from equation (30) and Bayesian p-values from Section B.2 of parameterestimates, $\hat{\theta}$ by time-to-death and sex. Tests are based based on the time-to-death- and sex-specific estimates of the Expanded Joint Model. #Res. refers to the number of parameter restrictions and DoF is the degrees of freedom.

Table A7: Hypothesis Tests of Hypothesis in Equation (7) by Time-to-death and Sex under Expanded Joint Model

	Hypothesis			Wald Test, E_F		Wald Test, E_g		Wald Test, E_ψ	
Group		#Res.	DoF	Test Stat.	P-value	Test Stat.	P-value	Test Stat.	P-value
**Female**									
TTD ∈[0;1.5)	H_0^1	3	115,212	10,462,566.678	0.0000000	1,321.115	0.0000000	97,032.573	0.0000000
	H_0^2	3	115,212	15,875,212.220	0.0000000	974.639	0.0000000	20,788.132	0.0000000
	H_0^3	1	115,212	1,108,896.386	0.0000000	866.128	0.0000000	91,030.012	0.0000000
TTD ∈[1.5;4)	H_0^1	3	189,120	4,287,850.570	0.0000000	370.818	0.0000000	30,652.950	0.0000000
	H_0^2	3	189,120	4,896,409.767	0.0000000	587.887	0.0000000	84,068.219	0.0000000
	H_0^3	1	189,120	101,223.585	0.0000000	194.668	0.0000000	590.985	0.0000000
TTD > 4	H_0^1	3	4,820,971	25,397.142	0.0000000	6.843	0.0326709	80.133	0.0000000
	H_0^2	3	4,820,971	60,029.854	0.0000000	20.859	0.0003377	12,892.384	0.0000000
	H_0^3	NA	NA	NA	NA	NA	NA	NA	NA
**Male**									
TTD ∈[0;1.5)	H_0^1	3	110,495	8,742,001.315	0.0000000	1,395.730	0.0000000	39,927.922	0.0000000
	H_0^2	3	110,495	22,091,912.522	0.0000000	461.846	0.0000000	68,867.218	0.0000000
	H_0^3	1	110,495	367,506.988	0.0000000	1,100.961	0.0000000	29,835.457	0.0000000
TTD ∈[1.5;4)	H_0^1	3	185,613	6,020,325.074	0.0000000	566.711	0.0000000	34,796.059	0.0000000
	H_0^2	3	185,613	4,332,540.643	0.0000000	511.055	0.0000000	35,323.126	0.0000000
	H_0^3	1	185,613	117,799.965	0.0000000	406.020	0.0000000	466.633	0.0000000
TTD > 4	H_0^1	3	4,578,361	125,356.597	0.0000000	9.031	0.0109355	418.503	0.0000000
	H_0^2	3	4,578,361	187,259.492	0.0000000	23.679	0.0000926	8,390.307	0.0000000
	H_0^3	NA	NA	NA	NA	NA	NA	NA	NA

Note: Wald test of marginal association by age and sex with null hypotheses in equation (7) based on the time-to-death- and sex-specific estimates of the Expanded Joint Model. #Res. refers to the number of parameter restrictions and DoF is the degrees of freedom.

C.2 Full Parameter Estimates

Table A8 presents the complete regression results corresponding to Section 4. Unlike Table 1, this table includes additional explanatory variables: the intercept, a male dummy, month dummies, and an indicator for whether time-to-death exceeds four years. Additionally, it reports the marginal effect on the probability of hospital expenditure utilization (E_F) and the extensive margin conditional on expenditure use (E_g).

Table A8: GLM model estimates

Model:	Naïve					Red Herring					Steepening					Joint Model				
	Probit	Poisson	E_F	E_g	E_ψ	Probit	Poisson	E_F	E_g	E_ψ	Probit	Poisson	E_F	E_g	E_ψ	Probit	Poisson	E_F	E_g	E_ψ
Intercept	-1.545	5.864	-0.444	5,313.659	773.987	-1.434	6.486	-0.410	5,877.241	945.912	-1.556	5.873	-0.445	5,321.162	774.038	-1.430	6.518	-0.406	5,905.710	955.257
	(0.569)	(0.008)	(0.001)	(4.170)	(527.275)	(0.541)	(0.005)	(0.004)	(4.499)	(540.010)	(0.585)	(0.010)	(0.004)	(5.278)	(529.684)	(0.555)	(0.005)	(0.008)	(4.173)	(542.393)
	[0.007]	[0.000]	[0.000]	[0.000]	[0.142]	[0.008]	[0.000]	[0.000]	[0.000]	[0.080]	[0.008]	[0.000]	[0.000]	[0.000]	[0.144]	[0.010]	[0.000]	[0.000]	[0.000]	[0.078]
Male	-0.131	0.127	-0.038	115.303	-7.691	-0.137	0.073	-0.039	66.231	-17.873	-0.133	0.128	-0.038	116.132	-7.942	-0.139	0.073	-0.040	66.316	-18.250
	(0.035)	(0.003)	(0.004)	(3.021)	(8.840)	(0.036)	(0.003)	(0.004)	(2.458)	(3.731)	(0.035)	(0.003)	(0.004)	(3.053)	(8.809)	(0.037)	(0.003)	(0.004)	(2.472)	(3.628)
	[0.000]	[0.000]	[0.000]	[0.000]	[0.384]	[0.000]	[0.000]	[0.000]	[0.000]	[0.000]	[0.000]	[0.000]	[0.000]	[0.000]	[0.367]	[0.000]	[0.000]	[0.000]	[0.000]	[0.000]
Age	0.014	0.017	0.004	15.465	6.944	0.012	0.003	0.003	3.002	3.430	0.011	0.018	0.004	15.577	6.882	0.008	0.003	0.003	3.045	3.324
	(0.004)	(0.000)	(0.000)	(0.083)	(1.890)	(0.003)	(0.000)	(0.000)	(0.026)	(0.517)	(0.003)	(0.000)	(0.000)	(0.083)	(1.896)	(0.002)	(0.000)	(0.000)	(0.027)	(0.504)
	[0.000]	[0.000]	[0.000]	[0.000]	[0.000]	[0.000]	[0.000]	[0.000]	[0.000]	[0.000]	[0.000]	[0.000]	[0.000]	[0.000]	[0.000]	[0.000]	[0.000]	[0.000]	[0.000]	[0.000]
TTD	-	-	-	-	-	-0.101	-0.470	-0.029	-425.949	-117.003	-	-	-	-	-	-0.103	-0.470	-0.029	-425.715	-117.407
	(-)	(-)	(-)	(-)	(-)	(0.019)	(0.002)	(0.005)	(2.469)	(42.194)	(-)	(-)	(-)	(-)	(-)	(0.020)	(0.002)	(0.005)	(2.447)	(41.915)
	[-]	[-]	[-]	[-]	[-]	[0.000]	[0.000]	[0.000]	[0.000]	[0.006]	[-]	[-]	[-]	[-]	[-]	[0.000]	[0.000]	[0.000]	[0.000]	[0.005]
Time	-	-	-	-	-	-	-	-	-	-	0.005	-0.002	0.007	-8.448	4.416	0.004	-0.004	0.007	-1.722	5.715
	(-)	(-)	(-)	(-)	(-)	(-)	(-)	(-)	(-)	(-)	(0.003)	(0.000)	(0.001)	(0.212)	(0.255)	(0.003)	(0.000)	(0.001)	(0.151)	(0.266)
	[-]	[-]	[-]	[-]	[-]	[-]	[-]	[-]	[-]	[-]	[0.119]	[0.000]	[0.000]	[0.000]	[0.000]	[0.221]	[0.000]	[0.000]	[0.000]	[0.000]
Age·Time	-	-	-	-	-	-	-	-	-	-	0.033	-0.011	0.011	7.028	11.435	0.037	0.003	0.010	1.354	9.147
	(-)	(-)	(-)	(-)	(-)	(-)	(-)	(-)	(-)	(-)	(0.006)	(0.001)	(0.001)	(0.163)	(1.659)	(0.006)	(0.000)	(0.001)	(0.145)	(0.785)
	[-]	[-]	[-]	[-]	[-]	[-]	[-]	[-]	[-]	[-]	[0.000]	[0.000]	[0.000]	[0.000]	[0.000]	[0.000]	[0.000]	[0.000]	[0.000]	[0.000]
TTD>4	-	-	-	-	-	0.593	1.912	0.169	1,732.858	519.551	-	-	-	-	-	0.619	1.911	0.176	1,731.149	524.220
	(-)	(-)	(-)	(-)	(-)	(0.121)	(0.006)	(0.028)	(7.499)	(176.522)	(-)	(-)	(-)	(-)	(-)	(0.128)	(0.006)	(0.028)	(7.392)	(175.611)
	[-]	[-]	[-]	[-]	[-]	[0.000]	[0.000]	[0.000]	[0.000]	[0.003]	[-]	[-]	[-]	[-]	[-]	[0.000]	[0.000]	[0.000]	[0.000]	[0.003]
Feb	0.004	-0.182	0.001	-165.054	-35.050	0.005	-0.178	0.001	-161.524	-34.227	0.002	-0.181	0.001	-164.063	-35.369	0.002	-0.178	0.001	-161.372	-34.714
	(0.001)	(0.006)	(0.000)	(4.868)	(15.439)	(0.001)	(0.005)	(0.000)	(4.478)	(13.816)	(0.001)	(0.006)	(0.000)	(4.866)	(15.367)	(0.001)	(0.005)	(0.000)	(4.476)	(13.749)
	[0.001]	[0.000]	[0.000]	[0.000]	[0.023]	[0.001]	[0.000]	[0.000]	[0.000]	[0.013]	[0.006]	[0.000]	[0.000]	[0.000]	[0.021]	[0.004]	[0.000]	[0.000]	[0.000]	[0.012]
Mar	0.032	-0.107	0.009	-97.192	-13.186	0.033	-0.105	0.009	-95.208	-13.112	0.028	-0.106	0.008	-95.656	-13.832	0.029	-0.105	0.008	-94.906	-14.018
	(0.009)	(0.006)	(0.001)	(5.748)	(8.619)	(0.009)	(0.006)	(0.001)	(5.766)	(7.713)	(0.008)	(0.006)	(0.001)	(5.726)	(8.531)	(0.008)	(0.006)	(0.001)	(5.765)	(7.699)
	[0.000]	[0.000]	[0.000]	[0.000]	[0.126]	[0.000]	[0.000]	[0.000]	[0.000]	[0.089]	[0.000]	[0.000]	[0.000]	[0.000]	[0.105]	[0.000]	[0.000]	[0.000]	[0.000]	[0.069]
Apr	0.023	-0.200	0.007	-181.627	-34.008	0.023	-0.196	0.007	-177.252	-33.251	0.017	-0.198	0.005	-179.357	-34.983	0.017	-0.195	0.005	-176.847	-34.604
	(0.006)	(0.008)	(0.001)	(7.336)	(16.499)	(0.007)	(0.008)	(0.001)	(7.336)	(14.654)	(0.005)	(0.008)	(0.000)	(7.305)	(16.383)	(0.005)	(0.008)	(0.000)	(7.331)	(14.625)
	[0.000]	[0.000]	[0.000]	[0.000]	[0.039]	[0.000]	[0.000]	[0.000]	[0.000]	[0.023]	[0.001]	[0.000]	[0.000]	[0.000]	[0.033]	[0.001]	[0.000]	[0.000]	[0.000]	[0.018]
May	0.041	-0.166	0.012	-150.510	-22.571	0.042	-0.160	0.012	-145.127	-21.854	0.034	-0.163	0.010	-147.697	-23.858	0.034	-0.160	0.010	-144.585	-23.607
	(0.012)	(0.005)	(0.001)	(4.833)	(13.817)	(0.012)	(0.005)	(0.001)	(4.467)	(12.424)	(0.010)	(0.005)	(0.001)	(4.833)	(13.662)	(0.010)	(0.005)	(0.001)	(4.450)	(12.427)
	[0.000]	[0.000]	[0.000]	[0.000]	[0.102]	[0.000]	[0.000]	[0.000]	[0.000]	[0.079]	[0.000]	[0.000]	[0.000]	[0.000]	[0.081]	[0.000]	[0.000]	[0.000]	[0.000]	[0.057]
Jun	0.048	-0.180	0.014	-162.966	-23.496	0.049	-0.174	0.014	-157.459	-22.825	0.039	-0.176	0.011	-159.221	-25.098	0.039	-0.173	0.011	-156.730	-25.048
	(0.013)	(0.004)	(0.001)	(3.434)	(15.242)	(0.014)	(0.004)	(0.001)	(3.439)	(13.718)	(0.011)	(0.004)	(0.001)	(3.410)	(15.046)	(0.011)	(0.004)	(0.001)	(3.413)	(13.735)
	[0.000]	[0.000]	[0.000]	[0.000]	[0.123]	[0.000]	[0.000]	[0.000]	[0.000]	[0.096]	[0.000]	[0.000]	[0.000]	[0.000]	[0.095]	[0.000]	[0.000]	[0.000]	[0.000]	[0.068]
Jul	0.022	-0.241	0.006	-218.463	-42.226	0.023	-0.242	0.006	-218.872	-42.496	0.010	-0.236	0.003	-214.210	-44.259	0.011	-0.241	0.003	-218.012	-45.201
	(0.006)	(0.006)	(0.001)	(5.040)	(20.565)	(0.007)	(0.005)	(0.001)	(4.444)	(18.945)	(0.003)	(0.006)	(0.000)	(5.079)	(20.360)	(0.003)	(0.005)	(0.000)	(4.458)	(18.955)
	[0.001]	[0.000]	[0.000]	[0.000]	[0.040]	[0.001]	[0.000]	[0.000]	[0.000]	[0.025]	[0.001]	[0.000]	[0.000]	[0.000]	[0.030]	[0.002]	[0.000]	[0.000]	[0.000]	[0.017]
Aug	0.055	-0.203	0.016	-183.896	-26.378	0.056	-0.201	0.016	-182.185	-26.635	0.041	-0.197	0.012	-178.674	-28.722	0.042	-0.200	0.012	-181.174	-29.839
	(0.016)	(0.006)	(0.001)	(5.132)	(16.714)	(0.016)	(0.005)	(0.001)	(4.724)	(15.215)	(0.012)	(0.006)	(0.001)	(5.174)	(16.454)	(0.012)	(0.005)	(0.001)	(4.733)	(15.274)
	[0.000]	[0.000]	[0.000]	[0.000]	[0.115]	[0.000]	[0.000]	[0.000]	[0.000]	[0.080]	[0.001]	[0.000]	[0.000]	[0.000]	[0.081]	[0.001]	[0.000]	[0.000]	[0.000]	[0.051]
Sep	0.074	-0.165	0.021	-149.856	-14.102	0.075	-0.160	0.022	-145.079	-13.900	0.059	-0.159	0.017	-144.145	-16.754	0.059	-0.159	0.017	-143.963	-17.494
	(0.020)	(0.006)	(0.002)	(5.266)	(12.688)	(0.020)	(0.006)	(0.002)	(5.170)	(11.273)	(0.016)	(0.006)	(0.002)	(5.317)	(12.412)	(0.016)	(0.006)	(0.002)	(5.185)	(11.368)
	[0.000]	[0.000]	[0.000]	[0.000]	[0.266]	[0.000]	[0.000]	[0.000]	[0.000]	[0.218]	[0.000]	[0.000]	[0.000]	[0.000]	[0.177]	[0.000]	[0.000]	[0.000]	[0.000]	[0.124]
Okt	0.074	-0.150	0.021	-136.130	-11.118	0.075	-0.148	0.021	-133.917	-11.489	0.057	-0.143	0.016	-129.609	-14.108	0.057	-0.146	0.016	-132.638	-15.548
	(0.020)	(0.005)	(0.002)	(4.012)	(11.549)	(0.020)	(0.005)	(0.002)	(4.023)	(10.472)	(0.015)	(0.005)	(0.002)	(4.069)	(11.241)	(0.016)	(0.005)	(0.002)	(4.038)	(10.600)
	[0.000]	[0.000]	[0.000]	[0.000]	[0.336]	[0.000]	[0.000]	[0.000]	[0.000]	[0.273]	[0.000]	[0.000]	[0.000]	[0.000]	[0.209]	[0.000]	[0.000]	[0.000]	[0.000]	[0.142]
Nov	0.080	-0.136	0.023	-123.210	-6.928	0.080	-0.135	0.023	-121.971	-7.633	0.060	-0.128	0.017	-115.928	-10.205	0.061	-0.133	0.017	-120.505	-12.097
	(0.021)	(0.009)	(0.002)	(7.705)	(9.389)	(0.022)	(0.008)	(0.002)	(7.111)	(8.642)	(0.016)	(0.009)	(0.002)	(7.800)	(9.059)	(0.016)	(0.008)	(0.002)	(7.163)	(8.798)
	[0.000]	[0.000]	[0.000]	[0.000]	[0.461]	[0.000]	[0.000]	[0.000]	[0.000]	[0.377]	[0.000]	[0.000]	[0.000]	[0.000]	[0.260]	[0.000]	[0.000]	[0.000]	[0.000]	[0.169]
Dec	0.041	-0.182	0.012	-164.713	-25.630	0.042	-0.181	0.012	-163.762	-25.843	0.020	-0.173	0.006	-156.697	-29.284	0.020	-0.179	0.006	-162.167	-30.776
	(0.011)	(0.003)	(0.001)	(2.935)	(15.092)	(0.012)	(0.003)	(0.001)	(2.783)	(13.760)	(0.006)	(0.003)	(0.001)	(2.987)	(14.735)	(0.006)	(0.003)	(0.001)	(2.800)	(13.920)
	[0.000]	[0.000]	[0.000]	[0.000]	[0.089]	[0.000]	[0.000]	[0.000]	[0.000]	[0.060]	[0.000]	[0.000]	[0.000]	[0.000]	[0.047]	[0.000]	[0.000]	[0.000]	[0.000]	[0.027]
DoF			9,999,972					9,999,968					9,999,968					9,999,964		
Log Lik.			-3,196,833,760					-2,906,760,856					-3,194,966,130					-2,906,648,274		

Note: TTD is short for time-to-death. Standard errors in parenthesis. P-values in squared brackets. Models are estimated with 10,000,000 observations and 2,191,490 in the Poisson part. E_F denote to the marginal probability of using hospital expenditures, E_g is the marginal amount used conditional on using any hospital expenditures while E_ψ refers to the marginal effects of the full two-part model. Age·Time is divided by 100 in the Probit and Poisson regressions but not in E_F, E_g, or E_ψ. Amounts in 2020 dollars.

C.3 Robustness by Sex

We examine whether the Red Herring and Steepening hypotheses differ by sex. Figures 1.(a) and 1.(b) illustrate minor variations in hospital expenditures across sexes. To investigate this further, Table A9 presents the full parameter estimates from the joint model in equation (5), including results from both the Probit and Poisson regressions. It also reports the marginal associations for (i) the probability of using hospital expenditures (E_F), (ii) the conditional amount spent (E_g), and (iii) the overall two-part model (E_ψ). Additionally, the table includes estimates for the intercept, month dummies, and an indicator for whether time-to-death exceeds four years.

Overall, the parameter estimates for males and females in Table A9 are largely consistent with the main population estimates in Table 1. Age remains positively associated with the probability of using hospital expenditures, as indicated by Probit estimates of 0.004 for females (column 1)

and 0.015 for males (column 6). Regarding time-to-death, being one year further from death reduces hospital expenditures, conditional on usage, with parameter estimates of -0.454 for females (column 2) and -0.484 for males (column 7). For the Steepening Hypothesis, the age-time interaction term is positive in the Probit model for both sexes, suggesting a Steepening effect. However, while males also exhibit a positive and significant estimate in the Poisson model, the parameter for females is insignificant. This suggests that steepening is evident on both margins for males but is absent on the extensive margin for females.

To formally assess whether our main findings hold across sexes, we replicate the three hypothesis tests from equation (6) using sex-disaggregated data (Table A10). The results confirm the main conclusions in Table 2: all three hypotheses are rejected for both sexes at the conventional 5% significance level. As in the main results, the Wald test for the marginal effects under hypothesis H_0^3 exhibits the highest p-value (2.1% for males), largely due to the high variance in time-to-death estimates.

A key question in health economics is whether population aging affects males and females differently, particularly given sex-specific differences in mortality rates. If changes in mortality rates vary by sex, hospital expenditure growth could also differ. To test whether age, time-to-death, and their interaction have equal associations for both sexes, we conduct a Welch test Welch on the parameter estimates in Table A9. The results, presented in Table A11, yield p-values below 5% for all marginal associations and key regressors. This indicates that we cannot reject the null hypothesis that the marginal effects are identical across sexes. Thus, at a population level, the association between population aging and hospital expenditures appears to be similar for males and females.

Table A10: Hypothesis tests of hypothesis in equation (6) by sex

	**Hypothesis**		**Wald Test | Parameter**		**Bayesian Test | Parameter**		**Wald Test | Marginal Effect**		
Sex		#Res.	Test Statistic	P-value	log Bayes Factor	P-value	#Res.	Test Statistic	P-value
Female	H_0^1	6	1,776,411	0.0000000	-37,683,552	0.0000000	3	8,352	0.0000000
	H_0^2	6	497,093	0.0000000	-1,311,301	0.0000000	3	5,690	0.0000000
	H_0^3	2	169,884	0.0000000	-37,464,562	0.0000000	1	6	0.0115288
Male	H_0^1	6	3,515,548	0.0000000	-47,334,375	0.0000000	3	3,810	0.0000000
	H_0^2	6	522,947	0.0000000	-1,024,901	0.0000000	3	1,328	0.0000000
	H_0^3	2	2,280,650	0.0000000	-47,261,128	0.0000000	1	5	0.0206969

Note: Hypothesis tests statistics and p-values. #Res. refers to the number of parameter restrictions. In the Bayesian test we set a 50% prior belief in the null.

Table A11: P-values of Welch Test of Similar Estimates for Females and Males

	E_F	E_g	E_ψ
Age	0.007	0.822	0.097
TTD	0.798	0.423	0.934
Age·Time	0.724	0.125	0.422

Note: Welch test of similar parameter estimates based on estimated in Table A9 for the full population of females and males, respectively. E_F denotes to the marginal probability of using hospital expenditures, E_g is the marginal amount used conditional on using any hospital expenditures while E_ψ refers to the marginal effects of the full two-part model.

Table A9: Estimates of Joint Model by Sex

	Female					Male				
	Probit	Poisson	E_F	E_g	E_ψ	Probit	Poisson	E_F	E_g	E_ψ
Intercept	-1.220	6.475	-0.372	5,473.67	1,021.79	-1.881	6.648	-0.488	6,542.20	858.62
	(0.281)	(0.164)	(0.011)	(293.255)	(340.653)	(0.290)	(0.074)	(0.051)	(147.030)	(371.777)
	[0.000]	[0.000]	[0.000]	[0.000]	[0.003]	[0.000]	[0.000]	[0.000]	[0.000]	[0.021]
Age	0.004	0.003	0.002	2.80	2.24	0.014	0.003	0.005	3.26	4.75
	(0.000)	(0.002)	(0.000)	(1.291)	(0.760)	(0.000)	(0.001)	(0.001)	(1.560)	(1.307)
	[0.000]	[0.032]	[0.000]	[0.030]	[0.003]	[0.000]	[0.023]	[0.000]	[0.037]	[0.000]
TTD	-0.092	-0.454	-0.028	-383.79	-114.45	-0.117	-0.484	-0.030	-476.58	-120.14
	(0.006)	(0.082)	(0.005)	(58.775)	(45.303)	(0.007)	(0.085)	(0.006)	(99.734)	(51.931)
	[0.000]	[0.000]	[0.000]	[0.000]	[0.012]	[0.000]	[0.000]	[0.000]	[0.000]	[0.021]
Age · Time	0.025	-0.001	0.010	0.30	8.10	0.049	0.009	0.011	2.67	10.36
	(0.002)	(0.001)	(0.002)	(0.453)	(1.405)	(0.004)	(0.004)	(0.002)	(1.480)	(2.442)
	[0.000]	[0.328]	[0.000]	[0.514]	[0.000]	[0.000]	[0.045]	[0.000]	[0.071]	[0.000]
Time	0.011	-0.002	0.008	-2.50	5.79	-0.005	-0.006	0.006	-0.68	5.47
	(0.000)	(0.001)	(0.002)	(0.916)	(0.628)	(0.001)	(0.003)	(0.001)	(0.318)	(1.093)
	[0.000]	[0.114]	[0.000]	[0.006]	[0.000]	[0.000]	[0.036]	[0.000]	[0.034]	[0.000]
$\mathbb{1}\{TTD > 4\}$	0.536	1.913	0.163	1,617.17	518.36	0.697	1.908	0.181	1,878.03	527.35
	(0.032)	(0.474)	(0.033)	(354.790)	(222.148)	(0.041)	(0.433)	(0.036)	(489.042)	(232.996)
	[0.000]	[0.000]	[0.000]	[0.000]	[0.020]	[0.000]	[0.000]	[0.000]	[0.000]	[0.024]
Feb	0.006	-0.158	0.002	-133.63	-30.63	-0.002	-0.199	-0.001	-196.20	-39.00
	(0.002)	(0.083)	(0.000)	(66.651)	(25.910)	(0.001)	(0.089)	(0.000)	(94.335)	(29.669)
	[0.000]	[0.058]	[0.000]	[0.045]	[0.237]	[0.164]	[0.025]	[0.037]	[0.038]	[0.189]
Mar	0.032	-0.082	0.010	-69.45	-8.97	0.025	-0.129	0.006	-126.82	-19.35
	(0.003)	(0.033)	(0.002)	(26.352)	(10.581)	(0.000)	(0.060)	(0.002)	(63.748)	(18.527)
	[0.000]	[0.014]	[0.000]	[0.008]	[0.397]	[0.000]	[0.033]	[0.000]	[0.047]	[0.296]
Apr	0.015	-0.170	0.005	-143.47	-30.78	0.020	-0.222	0.005	-218.96	-38.59
	(0.001)	(0.083)	(0.001)	(66.521)	(26.078)	(0.001)	(0.096)	(0.001)	(102.020)	(31.640)
	[0.000]	[0.042]	[0.000]	[0.031]	[0.238]	[0.000]	[0.021]	[0.000]	[0.032]	[0.223]
May	0.032	-0.132	0.010	-111.48	-19.05	0.037	-0.189	0.010	-186.44	-28.32
	(0.002)	(0.056)	(0.002)	(43.804)	(17.621)	(0.000)	(0.082)	(0.003)	(86.591)	(25.766)
	[0.000]	[0.018]	[0.000]	[0.011]	[0.280]	[0.000]	[0.020]	[0.000]	[0.031]	[0.272]
Jun	0.041	-0.154	0.012	-130.15	-21.43	0.038	-0.193	0.010	-190.23	-28.83
	(0.002)	(0.064)	(0.003)	(50.043)	(20.171)	(0.001)	(0.074)	(0.002)	(79.408)	(24.683)
	[0.000]	[0.015]	[0.000]	[0.009]	[0.288]	[0.000]	[0.009]	[0.000]	[0.017]	[0.243]
Jul	0.009	-0.215	0.003	-181.58	-41.52	0.013	-0.268	0.003	-264.18	-49.04
	(0.001)	(0.102)	(0.001)	(80.826)	(32.434)	(0.001)	(0.116)	(0.001)	(122.737)	(38.579)
	[0.000]	[0.035]	[0.000]	[0.025]	[0.201]	[0.000]	[0.020]	[0.001]	[0.031]	[0.204]
Aug	0.040	-0.182	0.012	-153.76	-27.22	0.043	-0.219	0.011	-215.83	-32.63
	(0.003)	(0.087)	(0.002)	(69.560)	(26.710)	(0.001)	(0.093)	(0.003)	(98.697)	(29.688)
	[0.000]	[0.037]	[0.000]	[0.027]	[0.308]	[0.000]	[0.018]	[0.000]	[0.029]	[0.272]
Sep	0.065	-0.124	0.020	-104.45	-9.42	0.053	-0.197	0.014	-194.12	-26.05
	(0.004)	(0.054)	(0.004)	(42.971)	(15.889)	(0.001)	(0.078)	(0.003)	(82.794)	(24.748)
	[0.000]	[0.023]	[0.000]	[0.015]	[0.553]	[0.000]	[0.011]	[0.000]	[0.019]	[0.292]
Okt	0.061	-0.116	0.019	-98.47	-8.94	0.053	-0.179	0.014	-175.91	-22.50
	(0.003)	(0.060)	(0.004)	(47.981)	(16.593)	(0.002)	(0.083)	(0.003)	(87.933)	(25.035)
	[0.000]	[0.052]	[0.000]	[0.040]	[0.590]	[0.000]	[0.032]	[0.000]	[0.045]	[0.369]
Nov	0.065	-0.112	0.020	-94.71	-6.96	0.056	-0.155	0.014	-153.00	-17.42
	(0.004)	(0.044)	(0.004)	(34.247)	(12.928)	(0.001)	(0.075)	(0.003)	(78.911)	(21.564)
	[0.000]	[0.010]	[0.000]	[0.006]	[0.590]	[0.000]	[0.038]	[0.000]	[0.053]	[0.419]
Dec	0.017	-0.132	0.005	-111.73	-22.78	0.024	-0.230	0.006	-226.50	-39.16
	(0.002)	(0.055)	(0.001)	(43.589)	(18.329)	(0.000)	(0.110)	(0.002)	(115.990)	(34.608)
	[0.000]	[0.017]	[0.000]	[0.010]	[0.214]	[0.000]	[0.037]	[0.000]	[0.051]	[0.258]
N	5,125,417	1,231,906	5,125,417	1,231,906	5,125,417	4,874,583	959,584	4,874,583	959,584	4,874,583
log Lik.			-1,470,477,613					-1,435,806,140		

E_F denote to the marginal probability of using hospital expenditures, E_g is the marginal amount used conditional on using any hospital expenditures while E_ψ refers to the marginal effects of the full two-part model. Standard errors in parenthesis. P-values in squared brackets. TTD is short for time-to-death. Age·Time is divided by 100 in the Probit and Poisson regressions but not in E_F, Eg, $or E_\psi$. Amounts in 2020 dollars.

C.4 Robustness by Age and Sex

Table A12 show the marginal associations by sex and age-groups for the full two-part model, E_ψ, at the extensive margin, E_F, and the intensive margin, E_g.

Table A13 shows the tests on marginal associations from Section B.2 for each age-sex-group. Table A14 performs the Wald test of Section 2.2 and the Bayesian p-value calculations from Section B.2. All tests reject the null hypotheses providing evidence in favor of the joint model as the alternative hypothesis including both time-to-death and Steepening effects.

Table A15 show Welch tests of equal means parameter estimates between two age-sex-groups for the three marginal effects, E_F, E_g, and E_ψ, respectively. The parameter estimates are as reported in Table A12. Lower diagonal elements of the tables hold the tests for the time-to-death parameter while the upper diagonal elements report the Welch test for the age-time interaction. Small p-values indicated that the null hypothesis of equal parameter estimates is strongly rejected, indicating that each group has a unique effect. Adjacent age-groups is more often not rejected. Table A16 perform the similar Welch tests but compares the parameter estimates between sexes for each age group. For most parameter estimates, females and males differ significantly, suggesting that population aging impacts genders in distinct ways.

Table A12: Marginal Association by Age Groups

Age Group	[30;40)		[40;50)		[50;60)		[60;70)		[70;80)		[80;90)		90+	
Sex	F	M	F	M	F	M	F	M	F	M	F	M	F	M
**Panel A: Full Model, E_ψ**														
Age	-5.34	1.71	1.92	3.72	2.79	5.76	4.98	9.23	3.15	-0.76	-6.33	-17.29	-18.02	-26.66
	(0.560)	(0.296)	(0.215)	(0.621)	(0.386)	(0.895)	(0.407)	(1.046)	(0.443)	(0.378)	(0.264)	(1.355)	(0.587)	(0.808)
	[0.000]	[0.000]	[0.000]	[0.000]	[0.000]	[0.000]	[0.000]	[0.000]	[0.000]	[0.044]	[0.000]	[0.000]	[0.000]	[0.000]
	{-10.56}	{7.08}	{7.84}	{7.85}	{8.78}	{9.10}	{10.49}	{10.81}	{10.88}	{14.26}	{-22.05}	{-29.85}	{-43.74}	{-73.03}
TTD	-92.48	-34.00	-91.99	-61.06	-124.18	-117.10	-163.51	-193.40	-217.17	-278.30	-215.68	-305.32	-176.62	-304.04
	(8.377)	(4.461)	(8.985)	(9.203)	(13.674)	(19.229)	(22.332)	(32.393)	(32.398)	(39.701)	(30.377)	(32.730)	(15.762)	(13.984)
	[0.000]	[0.000]	[0.000]	[0.000]	[0.000]	[0.000]	[0.000]	[0.000]	[0.000]	[0.000]	[0.000]	[0.000]	[0.000]	[0.000]
	{-11.29}	{-9.48}	{-10.05}	{-9.22}	{-11.02}	{-10.37}	{-12.59}	{-12.20}	{-15.48}	{-17.44}	{-20.07}	{-27.79}	{-40.44}	{-70.48}
Age·Time	-1.92	2.55	5.49	5.88	8.23	10.11	11.37	17.90	12.80	11.54	5.77	1.23	-7.65	-8.56
	(0.704)	(0.070)	(0.351)	(0.607)	(0.624)	(1.166)	(0.706)	(1.494)	(1.161)	(0.489)	(1.002)	(0.460)	(0.164)	(0.495)
	[0.006]	[0.000]	[0.000]	[0.000]	[0.000]	[0.000]	[0.000]	[0.000]	[0.000]	[0.000]	[0.000]	[0.007]	[0.000]	[0.000]
	{3.25}	{8.14}	{8.72}	{8.16}	{9.79}	{9.29}	{11.20}	{11.18}	{14.49}	{16.63}	{16.50}	{25.39}	{-51.07}	{-9.11}
**Panel B: Extensive Margin, Pr(H>0), E_F**														
Age	-0.006	0.002	0.002	0.003	0.003	0.006	0.005	0.009	0.002	0.005	-0.008	-0.008	-0.011	-0.012
	(0.001)	(0.000)	(0.000)	(0.000)	(0.000)	(0.001)	(0.000)	(0.001)	(0.000)	(0.000)	(0.000)	(0.000)	(0.000)	(0.000)
	[0.000]	[0.000]	[0.000]	[0.000]	[0.000]	[0.000]	[0.000]	[0.000]	[0.000]	[0.000]	[0.000]	[0.000]	[0.000]	[0.000]
	{-7.29}	{4.39}	{4.41}	{8.27}	{4.81}	{3.61}	{12.80}	{5.56}	{6.47}	{-13.86}	{4.59}	{-13.37}	{5.71}	{-46.21}
TTD	-0.052	-0.017	-0.060	-0.032	-0.065	-0.049	-0.064	-0.054	-0.050	-0.051	-0.030	-0.037	-0.017	-0.030
	(0.005)	(0.002)	(0.006)	(0.003)	(0.006)	(0.005)	(0.005)	(0.004)	(0.003)	(0.003)	(0.001)	(0.001)	(0.000)	(0.000)
	[0.000]	[0.000]	[0.000]	[0.000]	[0.000]	[0.000]	[0.000]	[0.000]	[0.000]	[0.000]	[0.000]	[0.000]	[0.000]	[0.000]
	{-30.87}	{-34.73}	{-60.15}	{-75.71}	{-47.11}	{-18.23}	{-15.52}	{-11.78}	{-11.25}	{-10.93}	{-11.51}	{-13.40}	{-17.87}	{-32.38}
Age·Time	0.000	0.005	0.008	0.007	0.011	0.011	0.014	0.017	0.013	0.017	0.005	0.006	-0.004	0.000
	(0.000)	(0.001)	(0.001)	(0.001)	(0.001)	(0.001)	(0.001)	(0.002)	(0.001)	(0.001)	(0.000)	(0.000)	(0.000)	(0.000)
	[0.001]	[0.000]	[0.000]	[0.000]	[0.000]	[0.000]	[0.000]	[0.000]	[0.000]	[0.000]	[0.000]	[0.000]	[0.000]	[0.000]
	{-5.15}	{-1.02}	{2.85}	{9.81}	{3.61}	{3.79}	{6.89}	{4.87}	{2.71}	{-11.14}	{-0.64}	{-10.01}	{-7.54}	{-28.24}
**Panel C: Intensive Margin, H| H>0, E_g**														
Age	-8.24	5.66	2.28	11.58	4.10	6.42	3.36	3.78	3.25	-16.81	10.65	-17.80	7.43	-28.00
	(1.130)	(1.290)	(0.517)	(1.402)	(0.853)	(1.779)	(0.262)	(0.679)	(0.502)	(1.213)	(2.320)	(1.332)	(1.300)	(0.584)
	[0.000]	[0.000]	[0.000]	[0.000]	[0.000]	[0.000]	[0.000]	[0.000]	[0.000]	[0.000]	[0.000]	[0.000]	[0.000]	[0.000]
	{-9.53}	{5.78}	{8.92}	{5.98}	{7.22}	{6.43}	{12.24}	{8.82}	{7.11}	{-2.01}	{-24.02}	{-12.76}	{-30.68}	{-32.98}
TTD	-292.01	-228.19	-303.68	-295.32	-353.21	-422.43	-410.47	-540.81	-519.21	-618.17	-562.93	-672.05	-623.33	-812.45
	(9.461)	(6.570)	(5.048)	(3.901)	(7.498)	(23.169)	(26.442)	(45.908)	(46.169)	(56.547)	(48.918)	(50.171)	(34.884)	(25.090)
	[0.000]	[0.000]	[0.000]	[0.000]	[0.000]	[0.000]	[0.000]	[0.000]	[0.000]	[0.000]	[0.000]	[0.000]	[0.000]	[0.000]
	{-11.04}	{-7.62}	{-10.24}	{-6.63}	{-9.08}	{-6.09}	{-7.32}	{-5.97}	{-6.70}	{-7.01}	{-7.10}	{-9.33}	{-11.21}	{-21.74}
Age·Time	-9.85	-0.53	2.43	8.83	4.60	5.16	1.75	6.04	1.59	-19.06	-0.88	-17.34	-7.96	-26.41
	(1.912)	(0.517)	(0.852)	(0.900)	(1.274)	(1.361)	(0.253)	(1.240)	(0.586)	(1.711)	(1.383)	(1.733)	(1.055)	(0.935)
	[0.000]	[0.310]	[0.004]	[0.000]	[0.000]	[0.000]	[0.000]	[0.000]	[0.007]	[0.000]	[0.524]	[0.000]	[0.000]	[0.000]
	{-2.73}	{36.35}	{15.65}	{9.68}	{13.19}	{8.67}	{16.11}	{11.98}	{11.02}	{23.57}	{5.75}	{2.68}	{-46.73}	{-17.31}
Controls	Yes	Yes	Yes	Yes	Yes	Yes	Yes	Yes	Yes	Yes	Yes	Yes	Yes	Yes
Num. Obs.	1,036,274	1,056,546	1,107,349	1,133,078	1,041,301	1,049,897	912,058	883,170	610,754	520,022	337,275	204,593	80,406	27,277
Num. Obs. E_g	210,671	112,176	200,262	150,483	242,061	191,218	254,788	231,560	198,025	186,890	106,966	78,561	19,133	8,696

Note: Dependent variable is monthly hospital expenditures in 2020 USD, estimated separately by age-sex group. E_F reports the average marginal effect on the probability of any hospital expenditure (extensive margin); E_g reports the average marginal effect on expenditures conditional on positive use (intensive margin); E_ψ reports the average marginal effect on expected monthly hospital expenditures from the full two-part model. Standard errors in round parentheses ( ); p-values in square brackets [ ]; Z-scores in curly brackets { }. TTD is short for time-to-death. Controls refer to an intercept, month dummies, time, and $\check{X}^*$. DoF is shorthand for degrees of freedom. Amounts in 2020 dollars.

Table A13: Hypothesis tests of hypothesis in equation (7) by Age and Sex

Ages	Hypothesis	#Res.	DoF	Wald Test, E_F Test Stat.	Wald Test, E_F P-value	Wald Test, E_g Test Stat.	Wald Test, E_g P-value	Wald Test, E_ψ Test Stat.	Wald Test, E_ψ P-value
**Female**									
[30;40)	H_0^1	3	1,036,240	537.940	0.0000000	14,310.151	0.0000000	75,930.582	0.0000000
	H_0^2	3	1,036,240	168.989	0.0000000	1,052.755	0.0000000	75,080.338	0.0000000
	H_0^3	1	1,036,240	127.536	0.0000000	775.019	0.0000000	121.870	0.0000000
[40;50)	H_0^1	3	1,107,315	1,039.280	0.0000000	10,483.417	0.0000000	7,213.309	0.0000000
	H_0^2	3	1,107,315	271.779	0.0000000	116.103	0.0000000	7,539.340	0.0000000
	H_0^3	1	1,107,315	101.100	0.0000000	977.815	0.0000000	104.820	0.0000000
[50;60)	H_0^1	3	1,041,267	981.961	0.0000000	53,678.173	0.0000000	11,510.002	0.0000000
	H_0^2	3	1,041,267	331.410	0.0000000	218.302	0.0000000	12,932.345	0.0000000
	H_0^3	1	1,041,267	121.549	0.0000000	25,143.413	0.0000000	82.469	0.0000000
[60;70)	H_0^1	3	912,024	1,144.813	0.0000000	613.369	0.0000000	6,793.449	0.0000000
	H_0^2	3	912,024	427.169	0.0000000	275.364	0.0000000	1,443.997	0.0000000
	H_0^3	1	912,024	158.383	0.0000000	601.445	0.0000000	53.610	0.0000000
[70;80)	H_0^1	3	610,720	923.526	0.0000000	865.385	0.0000000	4,270.132	0.0000000
	H_0^2	3	610,720	1,184.193	0.0000000	512.779	0.0000000	1,506.373	0.0000000
	H_0^3	1	610,720	239.697	0.0000000	179.026	0.0000000	44.934	0.0000000
[80;90)	H_0^1	3	337,241	1,700.001	0.0000000	7,189.324	0.0000000	11,773.575	0.0000000
	H_0^2	3	337,241	1,238.208	0.0000000	2,511.840	0.0000000	11,074.921	0.0000000
	H_0^3	1	337,241	402.685	0.0000000	145.778	0.0000000	50.413	0.0000000
90+	H_0^1	3	80,372	7,839.253	0.0000000	8,566.295	0.0000000	26,915.946	0.0000000
	H_0^2	3	80,372	8,980.677	0.0000000	7,323.404	0.0000000	7,321.818	0.0000000
	H_0^3	1	80,372	1,635.790	0.0000000	327.514	0.0000000	125.564	0.0000000
**Male**									
[30;40)	H_0^1	3	1,056,512	271.730	0.0000000	20,210.133	0.0000000	11,154.165	0.0000000
	H_0^2	3	1,056,512	208.034	0.0000000	302.235	0.0000000	7,035.333	0.0000000
	H_0^3	1	1,056,512	89.836	0.0000000	767.329	0.0000000	58.104	0.0000000
[40;50)	H_0^1	3	1,133,044	436.138	0.0000000	73,924.226	0.0000000	4,776.713	0.0000000
	H_0^2	3	1,133,044	228.542	0.0000000	197.512	0.0000000	7,619.332	0.0000000
	H_0^3	1	1,133,044	84.943	0.0000000	17,298.578	0.0000000	44.020	0.0000000
[50;60)	H_0^1	3	1,049,863	882.230	0.0000000	9,801.600	0.0000000	10,711.800	0.0000000
	H_0^2	3	1,049,863	354.013	0.0000000	35.313	0.0000001	9,169.842	0.0000000
	H_0^3	1	1,049,863	107.597	0.0000000	1,117.930	0.0000000	37.088	0.0000000
[60;70)	H_0^1	3	883,136	1,302.186	0.0000000	18,368.798	0.0000000	5,226.744	0.0000000
	H_0^2	3	883,136	412.629	0.0000000	113.877	0.0000000	5,576.746	0.0000000
	H_0^3	1	883,136	148.848	0.0000000	246.752	0.0000000	35.645	0.0000000
[70;80)	H_0^1	3	519,988	1,678.999	0.0000000	1,184.672	0.0000000	38,271.921	0.0000000
	H_0^2	3	519,988	1,886.165	0.0000000	549.226	0.0000000	1,001.603	0.0000000
	H_0^3	1	519,988	304.198	0.0000000	159.484	0.0000000	49.138	0.0000000
[80;90)	H_0^1	3	204,559	1,835.428	0.0000000	1,165.655	0.0000000	15,055.872	0.0000000
	H_0^2	3	204,559	5,092.486	0.0000000	1,115.840	0.0000000	1,827.501	0.0000000
	H_0^3	1	204,559	772.172	0.0000000	207.242	0.0000000	87.015	0.0000000
90+	H_0^1	3	27,243	11,264.858	0.0000000	8,205.288	0.0000000	97,796.000	0.0000000
	H_0^2	3	27,243	9,837.948	0.0000000	10,810.903	0.0000000	41,467.054	0.0000000
	H_0^3	1	27,243	4,967.937	0.0000000	1,117.005	0.0000000	472.675	0.0000000

Note: Wald test of marginal association by age and sex with null hypotheses in equation (7) based on the age- and sex-specific estimates in Table A12. #Res. refers to the number of parameter restrictions and DoF is the degrees of freedom.

Table A14: Hypothesis Tests of Age and Sex Specific Estimates

	Hypothesis		Wald Test		Bayes P-value	
Ages		#Res.	Test Stat.	P-value	Test Stat.	P-value
			**Female**			
[30;40)	H_0^1	6	227,188	0.0000000	-550,367	0.0000000
	H_0^2	6	199,989	0.0000000	-108,886	0.0000000
	H_0^3	2	17,225	0.0000000	-534,808	0.0000000
[40;50)	H_0^1	6	302,189	0.0000000	-1,681,246	0.0000000
	H_0^2	6	77,010	0.0000000	-10,187	0.0000000
	H_0^3	2	189,637	0.0000000	-1,678,097	0.0000000
[50;60)	H_0^1	6	2,503,715	0.0000000	-4,799,431	0.0000000
	H_0^2	6	251,567	0.0000000	-44,148	0.0000000
	H_0^3	2	183,711	0.0000000	-4,781,440	0.0000000
[60;70)	H_0^1	6	1,125,613	0.0000000	-8,629,363	0.0000000
	H_0^2	6	18,949	0.0000000	-23,793	0.0000000
	H_0^3	2	350,321	0.0000000	-8,611,648	0.0000000
[70;80)	H_0^1	6	3,207,222	0.0000000	-11,795,648	0.0000000
	H_0^2	6	84,520	0.0000000	-16,466	0.0000000
	H_0^3	2	1,004,722	0.0000000	-11,766,660	0.0000000
[80;90)	H_0^1	6	1,415,500	0.0000000	-9,053,124	0.0000000
	H_0^2	6	533,393	0.0000000	-135,431	0.0000000
	H_0^3	2	711,582	0.0000000	-8,870,034	0.0000000
90+	H_0^1	6	1,954,533	0.0000000	-2,002,936	0.0000000
	H_0^2	6	144,568	0.0000000	-64,413	0.0000000
	H_0^3	2	203,080	0.0000000	-1,907,422	0.0000000
			**Male**			
[30;40)	H_0^1	6	120,456	0.0000000	-427,175	0.0000000
	H_0^2	6	94,158	0.0000000	-98,405	0.0000000
	H_0^3	2	32,477	0.0000000	-349,514	0.0000000
[40;50)	H_0^1	6	685,141	0.0000000	-1,513,077	0.0000000
	H_0^2	6	171,119	0.0000000	-133,759	0.0000000
	H_0^3	2	81,655	0.0000000	-1,489,476	0.0000000
[50;60)	H_0^1	6	1,857,623	0.0000000	-5,531,109	0.0000000
	H_0^2	6	515,335	0.0000000	-44,705	0.0000000
	H_0^3	2	1,322,665	0.0000000	-5,519,590	0.0000000
[60;70)	H_0^1	6	1,415,140	0.0000000	-13,112,830	0.0000000
	H_0^2	6	412,511	0.0000000	-28,891	0.0000000
	H_0^3	2	487,213	0.0000000	-13,108,051	0.0000000
[70;80)	H_0^1	6	7,700,791	0.0000000	-16,484,131	0.0000000
	H_0^2	6	204,314	0.0000000	-213,487	0.0000000
	H_0^3	2	2,836,682	0.0000000	-16,401,831	0.0000000
[80;90)	H_0^1	6	2,021,666	0.0000000	-9,949,476	0.0000000
	H_0^2	6	117,942	0.0000000	-94,051	0.0000000
	H_0^3	2	462,470	0.0000000	-9,917,107	0.0000000
90+	H_0^1	6	6,159,660	0.0000000	-1,567,170	0.0000000
	H_0^2	6	1,518,007	0.0000000	-43,961	0.0000000
	H_0^3	2	1,668,405	0.0000000	-1,516,911	0.0000000

Note: Wald test from Section 2.2 and Bayesian p-values from Section B.2 of parameterestimates, $\hat{\theta}$ by age and sex. Tests are based based on the age- and sex-specific estimates in Table A12. #Res. refers to the number of parameter restrictions and DoF is the degrees of freedom.

Table A15: P-values of Equal Means Welch Tests for Time-to-Death (lower diagonal) and Age-Time Interaction (upper diagonal) by Age and Sex Groups

		A·T, Female							A·T, Male						
		[30;40)	[40;50)	[50;60)	[60;70)	[70;80)	[80;90)	90+	[30;40)	[40;50)	[50;60)	[60;70)	[70;80)	[80;90)	90+
		**Panel A: Extensive Margin, Pr(H>0), E_F**													
	[30;40)		0.000	0.000	0.000	0.000	0.000	0.000		0.028	0.000	0.000	0.000	0.009	0.000
	[40;50)	0.306		0.048	0.000	0.001	0.000	0.000	0.000		0.003	0.000	0.000	0.494	0.000
	[50;60)	0.080	0.518		0.067	0.278	0.000	0.000	0.000	0.003		0.003	0.000	0.000	0.000
TTD	[60;70)	0.090	0.618	0.844		0.310	0.000	0.000	0.000	0.000	0.452		0.900	0.000	0.000
	[70;80)	0.764	0.166	0.028	0.027		0.000	0.000	0.000	0.000	0.729	0.578		0.000	0.000
	[80;90)	0.000	0.000	0.000	0.000	0.000		0.000	0.000	0.161	0.015	0.000	0.000		0.000
	90+	0.000	0.000	0.000	0.000	0.000	0.000		0.000	0.467	0.000	0.000	0.000	0.000	
		**Panel B: Intensive Margin, H| H>0, E_g**													
	[30;40)		0.000	0.000	0.000	0.000	0.000	0.388		0.000	0.000	0.000	0.000	0.000	0.000
	[40;50)	0.277		0.156	0.443	0.417	0.042	0.000	0.000		0.025	0.069	0.000	0.000	0.000
	[50;60)	0.000	0.000		0.028	0.032	0.004	0.000	0.000	0.000		0.634	0.000	0.000	0.000
TTD	[60;70)	0.000	0.000	0.037		0.806	0.062	0.000	0.000	0.000	0.021		0.000	0.000	0.000
	[70;80)	0.000	0.000	0.000	0.041		0.100	0.000	0.000	0.000	0.001	0.288		0.483	0.000
	[80;90)	0.000	0.000	0.000	0.006	0.516		0.000	0.000	0.000	0.000	0.054	0.476		0.000
	90+	0.000	0.000	0.000	0.000	0.072	0.315		0.000	0.000	0.000	0.000	0.002	0.012	
		**Panel C: Full Model, E_ψ**													
	[30;40)		0.000	0.000	0.000	0.000	0.000	0.000		0.000	0.000	0.000	0.000	0.004	0.000
	[40;50)	0.968		0.000	0.000	0.000	0.797	0.000	0.008		0.001	0.000	0.000	0.000	0.000
	[50;60)	0.048	0.049		0.001	0.001	0.037	0.000	0.000	0.009		0.000	0.259	0.000	0.000
TTD	[60;70)	0.003	0.003	0.133		0.293	0.000	0.000	0.000	0.000	0.043		0.000	0.000	0.000
	[70;80)	0.000	0.000	0.008	0.173		0.000	0.000	0.000	0.000	0.000	0.098		0.000	0.000
	[80;90)	0.000	0.000	0.006	0.166	0.973		0.000	0.000	0.000	0.000	0.015	0.599		0.000
	90+	0.000	0.000	0.012	0.632	0.260	0.254		0.000	0.000	0.000	0.002	0.541	0.971	

Note: Welch test of equal parameter estimates by age and sex groups for estimates in Table A12. P-values for time-to-death on lower diagonal elements and the age-time interaction are reported in upper diagonal elements.

Table A16: P-values of Equal Means Welch Tests for Females and Males by Age Group

	[30;40)	[40;50)	[50;60)	[60;70)	[70;80)	[80;90)	90+
**Panel A: Extensive Margin, Pr(H>0), E_F**							
Age	0.000	0.124	0.000	0.000	0.000	0.290	0.505
TTD	0.000	0.000	0.037	0.165	0.828	0.000	0.000
Age·Time	0.000	0.311	0.901	0.144	0.002	0.000	0.000
**Panel B: Intensive Margin, H| H>0, E_g**							
Age	0.000	0.000	0.239	0.562	0.000	0.000	0.000
TTD	0.000	0.190	0.004	0.014	0.175	0.119	0.000
Age·Time	0.000	0.000	0.765	0.001	0.000	0.000	0.000
**Panel C: Full Model, E_ψ**							
Age	0.000	0.006	0.002	0.000	0.000	0.000	0.000
TTD	0.000	0.016	0.764	0.448	0.233	0.045	0.000
Age·Time	0.000	0.585	0.154	0.000	0.317	0.000	0.082

Note: Welch test of equal parameter estimates for females and males by age groups for estimates in Table A12.

C.5 Robustness by Time-to-death and Sex

Table A17 marginal associations by sex and time-to-death groups for the full two-part model, E_ψ, at the extensive margin, E_F, and the intensive margin, E_g.

Table A19 presents the results of Wald tests for the hypotheses outlined in Section B.2. Table A20 performs the Wald test of Section 2.2 and the Bayesian p-value calculations from Section B.2. When considering individuals with $TTD < 4$, we exclude the zero-restrictions on time-to-death in the hypotheses. Across all cases, we reject the hypotheses, providing strong evidence in favor of the alternative hypothesis, i.e., the joint model and the coexistence of the Red Herring and Steepening Hypotheses.

Table A18 examines whether the marginal effects differ across time-to-death groups for each sex using Welch tests. A p-value of zero indicates a rejection of the null hypothesis, confirming that the effects are significantly different between groups. However, for some comparisons, p-values exceed conventional significance levels of 5%, suggesting that differences may not be statistically meaningful. Notably, for males, the Steepening effect $(A \cdot T)$ on the extensive margin does not differ significantly between the $[0, 1.5)$ and 4+ time-to-death groups, as indicated by a high p-value of 69.1%. This implies that we cannot reject the null hypothesis that these groups exhibit identical Steepening effects. Table A21 perform the similar Welch tests but compares the parameter estimates between sexes for each age group. For most parameter estimates, females and males differ significantly, suggesting that population aging impacts them in distinct ways.

Table A18: P-values of Equal Means Welch Tests for Time-to-Death (lower diagonal) and Age-Time Interaction (upper diagonal) by Time-to-death and Sex Groups

		A·T, Female			A·T, Male		
		[0;1.5)	[1.5;4)	4+	[0;1.5)	[1.5;4)	4+
		**Panel A: Extensive Margin, Pr(H>0), E$_F$**					
TTD	[0; 1.5)		0.000	0.000		0.000	0.691
	[1.5; 4)	0.000		0.071	0.000		0.090
		**Panel B: Intensive Margin, H| H>0, E$_g$**					
TTD	[0; 1.5)		0.398	0.000		0.000	0.000
	[1.5; 4)	0.000		0.000	0.000		0.000
		**Panel C: Full Model, E$_\psi$**					
TTD	[0; 1.5)		0.000	0.000		0.000	0.000
	[1.5; 4)	0.000		0.000	0.000		0.000

Note: Welch test of equal parameter estimates by age and sex groups for estimates in Table A17. P-values for time-to-death on lower diagonal elements and the age-time interaction are reported in upper diagonal elements.

Table A17: Marginal Association by Time-to-Death Groups

	TTD∈[0;1.5)		TTD∈[1.5;4)		TTD>4	
Sex	F	M	F	M	F	M
**Panel A: Full Model, E$_\psi$**						
Age	-59.40	-36.57	-14.93	-7.68	2.87	4.97
	(4.623)	(2.682)	(1.313)	(1.000)	(1.014)	(1.490)
	[0.000]	[0.000]	[0.000]	[0.000]	[0.005]	[0.001]
	{-38.23}	{-39.87}	{-28.54}	{29.44}	{4.69}	{4.59}
TTD	-2,254.46	-2,928.96	-113.99	-146.07	-	-
	(192.133)	(241.446)	(13.018)	(15.171)	(-)	(-)
	[0.000]	[0.000]	[0.000]	[0.000]	[-]	[-]
	{-38.48}	{-43.76}	{-27.03}	{-29.70}	{-}	{-}
Age· Time	-5.84	40.06	3.88	14.74	6.92	8.11
	(0.735)	(2.953)	(0.062)	(0.376)	(0.505)	(1.221)
	[0.000]	[0.000]	[0.000]	[0.000]	[0.000]	[0.000]
	{5.72}	{44.10}	{28.79}	{30.57}	{4.86}	{4.74}
**Panel B: Extensive Margin, Pr(H>0), E$_F$**						
Age	-0.010	-0.002	-0.006	0.001	0.002	0.005
	(0.000)	(0.000)	(0.000)	(0.000)	(0.001)	(0.001)
	[0.000]	[0.000]	[0.000]	[0.000]	[0.000]	[0.000]
	{-15.30}	{-18.61}	{-13.30}	{-12.79}	{2.72}	{2.50}
TTD	-0.137	-0.144	-0.017	-0.022	-	-
	(0.004)	(0.003)	(0.001)	(0.001)	(-)	(-)
	[0.000]	[0.000]	[0.000]	[0.000]	[-]	[-]
	{-15.24}	{-15.48}	{-11.86}	{-13.47}	{-}	{-}
Age·Time	0.000	0.010	0.006	0.014	0.010	0.011
	(0.000)	(0.000)	(0.000)	(0.000)	(0.002)	(0.002)
	[0.000]	[0.000]	[0.000]	[0.000]	[0.000]	[0.000]
	{-12.27}	{11.20}	{-17.33}	{-9.11}	{1.72}	{1.78}
**Panel C: Intensive Margin, H| H>0, E$_g$**						
Age	-61.94	-59.16	-17.70	-22.73	5.64	8.00
	(4.050)	(3.179)	(1.331)	(1.777)	(2.071)	(3.208)
	[0.000]	[0.000]	[0.000]	[0.000]	[0.006]	[0.013]
	{-12.85}	{-13.63}	{-11.38}	{-7.68}	{2.83}	{3.33}
TTD	-4,070.09	-4,693.04	-234.39	-269.15	-	-
	(267.076)	(303.080)	(19.763)	(19.981)	(-)	(-)
	[0.000]	[0.000]	[0.000]	[0.000]	[-]	[-]
	{-11.73}	{-12.13}	{-8.76}	{-9.63}	{-}	{-}
Age·Time	-13.09	14.73	-14.22	-17.47	0.50	1.52
	(1.066)	(1.316)	(0.821)	(1.918)	(0.290)	(0.856)
	[0.000]	[0.000]	[0.000]	[0.000]	[0.085]	[0.076]
	{-7.95}	{13.57}	{62.22}	{39.18}	{13.68}	{6.64}
Controls	Yes	Yes	Yes	Yes	Yes	Yes
Num. Obs.	115,256	110,539	189,158	185,651	4,821,003	4,578,393
Num. Obs. E_g	52,070	57,501	71,025	77,266	1,108,811	824,817

Note: Dependent variable is monthly hospital expenditures in 2020 USD, estimated separately by time-to-death and sex group. E$_F$ reports the average marginal effect on the probability of any hospital expenditure (extensive margin); E$_g$ reports the average marginal effect on expenditures conditional on positive use (intensive margin); E$_\psi$ reports the average marginal effect on expected monthly hospital expenditures from the full two-part model. Standard errors in round parentheses ( ); p-values in square brackets [ ]; Z-scores in curly brackets { }. TTD is short for time-to-death. Controls include an intercept, month dummies, time, and $\check{X}^*$. Amounts in 2020 dollars.

Table A19: Hypothesis Tests of Hypothesis in Equation (7) by Time-to-death and Sex

Group		Hypothesis		Wald Test, E_F		Wald Test, E_g		Wald Test, E_ψ	
		#Res.	DoF	Test Stat.	P-value	Test Stat.	P-value	Test Stat.	P-value
**Female**									
TTD ∈[0;1.5)	H_0^1	3	115,224	13,833.874	0.0000000	271.134	0.0000000	3,451.547	0.0000000
	H_0^2	3	115,224	15,568.198	0.0000000	286.944	0.0000000	5,107.139	0.0000000
	H_0^3	1	115,224	1,481.035	0.0000000	250.342	0.0000000	137.683	0.0000000
TTD ∈[1.5;4)	H_0^1	3	189,126	3,033.856	0.0000000	1,577.919	0.0000000	9,539.253	0.0000000
	H_0^2	3	189,126	2,393.684	0.0000000	662.802	0.0000000	7,036.283	0.0000000
	H_0^3	1	189,126	730.559	0.0000000	145.214	0.0000000	76.673	0.0000000
TTD > 4	H_0^1	2	4,820,973	45.680	0.0000000	64.677	0.0000000	15,459.218	0.0000000
	H_0^2	3	4,820,973	736.895	0.0000000	347.148	0.0000000	31,191.826	0.0000000
	H_0^3	NA	NA	NA	NA	NA	NA	NA	NA
**Male**									
TTD ∈[0;1.5)	H_0^1	3	110,507	5,989.685	0.0000000	6,017.486	0.0000000	2,358.600	0.0000000
	H_0^2	3	110,507	6,088.672	0.0000000	5,653.367	0.0000000	657.069	0.0000000
	H_0^3	1	110,507	1,914.655	0.0000000	250.019	0.0000000	147.159	0.0000000
TTD ∈[1.5;4)	H_0^1	3	185,619	2,159.883	0.0000000	714.859	0.0000000	8,355.548	0.0000000
	H_0^2	3	185,619	2,851.027	0.0000000	1,116.048	0.0000000	37,239.445	0.0000000
	H_0^3	1	185,619	882.331	0.0000000	178.660	0.0000000	92.700	0.0000000
TTD > 4	H_0^1	2	4,578,363	80.817	0.0000000	7.477	0.0581444	7,705.467	0.0000000
	H_0^2	3	4,578,363	3,548.025	0.0000000	89.428	0.0000000	7,932.998	0.0000000
	H_0^3	NA	NA	NA	NA	NA	NA	NA	NA

Note: Wald test of marginal association by age and sex with null hypotheses in equation (7) based on the time-to-death- and sex-specific estimates in Table A17. #Res. refers to the number of parameter restrictions and DoF is the degrees of freedom.

Table A20: Hypothesis Tests of Age and Time-to-death Specific Estimates

Age Group		Hypothesis	Wald Test		Bayes P-value	
		#Res.	Test Statistic	P-value	Test Statistic	P-value
**Female**						
TTD ∈[0;1.5)	H_0^1	6	1,127,764	0.0000000	-21,430,154	0.0000000
	H_0^2	6	6,063,297	0.0000000	-5,292,636	0.0000000
	H_0^3	2	218,270	0.0000000	-21,091,715	0.0000000
TTD ∈[1.5;4)	H_0^1	6	3,416,159	0.0000000	-843,593	0.0000000
	H_0^2	6	11,924,339	0.0000000	-1,326,430	0.0000000
	H_0^3	2	389,942	0.0000000	-668,344	0.0000000
TTD > 4	H_0^1	4	118,728	0.0000000	-489,283	0.0000000
	H_0^2	6	5,280,376	0.0000000	-6,563,522	0.0000000
	H_0^3	NA	NA	NA	NA	NA
**Male**						
TTD ∈[0;1.5)	H_0^1	6	2,225,400	0.0000000	-29,634,778	0.0000000
	H_0^2	6	7,516,677	0.0000000	-4,833,063	0.0000000
	H_0^3	2	12,963	0.0000000	-28,818,441	0.0000000
TTD ∈[1.5;4)	H_0^1	6	1,208,504	0.0000000	-1,109,253	0.0000000
	H_0^2	6	4,394,037	0.0000000	-1,959,967	0.0000000
	H_0^3	2	145,172	0.0000000	-938,478	0.0000000
TTD > 4	H_0^1	4	210,291	0.0000000	-489,257	0.0000000
	H_0^2	6	5,648,288	0.0000000	-7,416,481	0.0000000
	H_0^3	NA	NA	NA	NA	NA

Note: Wald test from Section 2.2 and Bayesian p-values from Section B.2 of parameterestimates, $\hat{\theta}$ by time-to-death and sex. Tests are based based on the time-to-death- and sex-specific estimates in Table A17. #Res. refers to the number of parameter restrictions and DoF is the degrees of freedom.

Table A21: P-values of Equal Means Welch Tests for Females and Males by Time-to-death Group

	[0;1.5)	[1.5;4)	4+
**Panel A: Extensive Margin, Pr(H>0), E_F**			
Age	0.000	0.124	0.000
TTD	0.000	0.000	0.037
Age·Time	0.000	0.311	0.901
**Panel B: Intensive Margin, H| H>0, E_g**			
Age	0.000	0.000	0.239
TTD	0.000	0.190	0.004
Age·Time	0.000	0.000	0.765
**Panel C: Full Model, E_ψ**			
Age	0.000	0.006	0.002
TTD	0.000	0.016	0.764
Age·Time	0.000	0.585	0.154

Note: Welch test of equal parameter estimates for females and males by age groups for estimates in Table A17.

C.6 Robustness: Decomposition

Table A22 shows the decomposition of hospital expenditure changes for years 2005 to 2015. Results are close to the main estimates for years 2002 and 2017 in Table 5.

Table A22: Decomposition of Hospital Care Expenditure Differences between 2005 and 2015

	Total	Pop. Change		Healthc. Change		Naïve		TTD Error		Steepening		TTD Steep.	
	$mio.	$mio.	%	$mio.	%	$mio.	%	$mio.	%	$mio.	%	$mio.	%
All	2359.9	541.2	22.9	1818.7	77.1	1027.1	43.5	-485.9	-20.6	1332.8	56.5	485.9	20.6
Female	1131.9	155.8	13.8	976.1	86.2	373.5	33.0	-217.7	-19.2	758.4	67.0	217.7	19.2
Male	1228.0	411.7	33.5	816.3	66.5	680.8	55.4	-269.2	-21.9	547.2	44.6	269.2	21.9

References

Carreras, M., Ibern, P., and Inoriza, J. M. (2018). Ageing and healthcare expenditures: Exploring the role of individual health status. *Health Economics*, 27(5):865–876. A4

Engle, R. F. (1984). Wald, likelihood ratio, and Lagrange multiplier tests in econometrics. *Handbook of Econometrics*, 2:775–826. A1

Geue, C., Briggs, A., Lewsey, J., and Lorgelly, P. (2014). Population ageing and healthcare expenditure projections: New evidence from a time to death approach. *The European Journal of Health Economics*, 15(8):885–896. A4

Gregersen, F. A. (2014). The impact of ageing on health care expenditures: A study of steepening. *The European Journal of Health Economics*, 15(9):979–989. A1, A4

Howdon, D. and Rice, N. (2018). Health care expenditures, age, proximity to death and morbidity: Implications for an ageing population. *Journal of Health Economics*, 57:60–74. A4

Hyun, K.-R., Kang, S., and Lee, S. (2016). Population aging and healthcare expenditure in Korea. *Health Economics*, 25(10):1239–1251. A4

Karlsson, M. and Klohn, F. (2014). Testing the red herring hypothesis on an aggregated level: ageing, time-to-death and care costs for older people in sweden. *The European Journal of Health Economics*, 15(5):533–551. A4

Kass, R. E. and Raftery, A. E. (1995). Bayes factors. *Journal of the American Statistical Association*, 90(430):773–795. A2, A3

Kollerup, A., Kjellberg, J., and Ibsen, R. (2022). Ageing and health care expenditures: The importance of age per se, steepening of the individual-level expenditure curve, and the role of morbidity. *The European Journal of Health Economics*, pages 1–29. A4

Wong, A., van Baal, P. H., Boshuizen, H. C., and Polder, J. J. (2011). Exploring the influence of proximity to death on disease-specific hospital expenditures: A carpaccio of red herrings. *Health Economics*, 20(4):379–400. A4

Wooldridge, J. M. (2010). *Econometric Analysis of Cross Section and Panel Data.* MIT press. A2
